# Acidic fluids in the Earth’s lower crust

**DOI:** 10.1038/s41598-021-00719-3

**Published:** 2021-10-27

**Authors:** Vinod O. Samuel, M. Santosh, Yirang Jang, Sanghoon Kwon

**Affiliations:** 1 Department of Earth System Sciences, Yonsei University, Seoul, 03722 Republic of Korea; 2 School of Earth Sciences and Resources, China University of Geosciences Beijing, 29 Xueyuan Road, Beijing, 100083 China; 3 Department of Earth Sciences, University of Adelaide, Adelaide, SA Australia; 4 Department of Earth and Environmental Science, Chonnam National University, Gwangju, 61186 Republic of Korea

**Keywords:** Precambrian geology, Petrology

## Abstract

Fluid flux through Earth’s surface and its interior causes geochemical cycling of elements in the Earth. Quantification of such process needs accurate knowledge about the composition and properties of the fluids. Knowledge about the fluids in Earth’s interior is scarce due to limitations in both experimental methods and thermodynamic modeling in high/ultrahigh pressure–temperature conditions. In this study, we present halogen (Cl, F) measurements in apatite grains from the mafic (metagabbro), and felsic (two-pyroxene granulite, charnockite, hornblende-biotite gneiss) rocks preserved in the Nilgiri Block, southern India. Previous experiments show that it is difficult to incorporate Cl in apatite compared to F at high pressure and temperature conditions. Based on regional trends in Cl and F content in apatite (with highest Cl content 2.95 wt%), we suggest the presence of acidic C–O–H fluids in the lower crust (~20–40 km deep) during the high-grade metamorphism of these rocks. These fluids are capable of causing extreme chemical alterations of minerals, especially refractory ones. They also have significant potential for mass transfer, causing extensive geochemical variations on a regional scale and altering the chemical and isotope records of rocks formed in the early Earth. Our findings have important relevance in understanding speciation triggered by acidic fluids in the lower crust, as well as the role of fluids in deep Earth processes.

## Introduction

Earth as a system witnesses constant exchange of elements through its different reservoirs, such as atmosphere–hydrosphere–solid Earth^1–3^. Understanding geochemical cycles is crucial in solving the uncertainties in evolution models of the solid Earth and its environment^2–4^ with implications on the habitability of the planet. Fluids play a crucial role in the geochemical cycle of the Earth’s interior^1^. However, our knowledge on the nature and composition of these fluids including redox potential and acid–base mechanisms at high/ultrahigh pressure (*P*)-temperature (*T*) conditions in the lithosphere is limited^5,6^. The only fluid remnants observed so far in deep crustal rock inclusions are either pure CO_2_ of variable density or high salinity brines, either miscible or immiscible^7^. Recent thermodynamic^5^ and experimental^6^ models propose a mildly alkaline nature of deep fluids, derived from subduction. It needs to be evaluated whether these fluids are unique or represent the possibility of unknown extreme conditions^8–11^.

**Figure 1:**
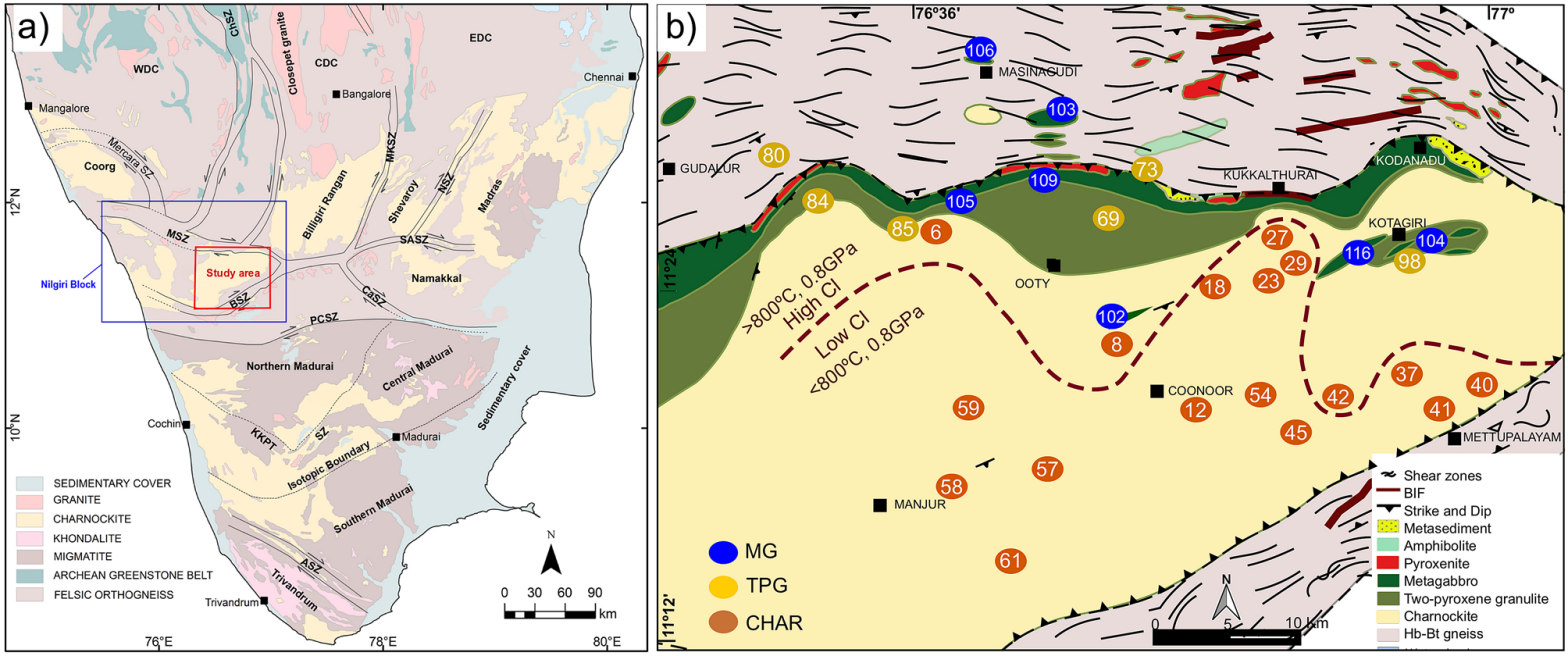
Regional geology and sample locations. (**a**) Regional geology and tectonic framework of southern India (after Ref.^37^); Blue box outlines the Nilgiri Block. An isotopic boundary divides the Madurai Block into Archean and Mesoproterozoic based on Sm–Nd whole rock and U–Pb ages^38^. (**b**) Geological map of the Nilgiri massif. Sample locations annotated by sample numbers are plotted on the map. The numbers are the same as those used in Ref.^20^, and are also listed in the Extended Data Table 1. Brown-dashed line separates areas of relatively high (> 800 °C) and low (< 800 °C) peak-metamorphic temperatures. *CHAR* charnockite samples, *TPG* two-pyroxene granulite samples and *MG* metagabbro samples. (**a**) Is reproduced by permission from Elsevier (see acknowledgements). (**b**) Is created using QGIS 2.6.1 (https://www.qgis.org/en/site/); (**a**) and (**b**) are modified using Inkscape version 0.92.2 (https://inkscape.org).

Here we report fluid-assisted recrystallization (during metamorphism) processes that occurred in deep crustal rocks as old as 2.7–2.5 Ga^12–14^ from the Nilgiri Block, southern India (Fig. 1), a major high-grade crustal block located to the south of the Archean Dharwar Craton in Peninsula India^15,16^ (Fig. 1a). Extensive mineralogical, textural, geochemical, geochronological and thermodynamic *P*–*T*–H_2_O activity (a_H2O_) calculations of equilibrium mineral assemblages show that these mafic and felsic rocks were formed due to differentiation of underplated mafic magma in the magma chamber during arc magmatic processes (*ca.* 2.7 Ga), and have then undergone anhydrous granulite facies metamorphism (*ca.* 2.5 Ga) at depths of ~20 to 40 km^14,17–20^. Fluid inclusions in the granulites from this terrane, and related mineral textural and thermodynamic data suggest that fluids played an important role during the transformation of mineral assemblages in the magmatic protolith to the recrystallized high-grade metamorphic rock^20–24^. In addition, fluid inclusions and textural studies in granulites worldwide suggest that CO_2_ and highly saline fluids/brines might represent the major fluid compositions^25–29^. These data, along with textural and experimental results suggest the possibility of fluid-assisted metamorphism in the presence of H_2_O–CO_2_-saline fluids/brines^30–36^.

Apatite is a common accessory mineral in the lower crustal rocks, and is widely used to trace fluid compositions^8,32,39–44^. Apatite undergoes halogen-(F,Cl)-exchange reactions with fluids such that its final composition reflects the chemical nature of the associated fluids^8,41–44^. In previous natural examples of high Cl bearing apatite (3–6 wt% Cl) reported in metamorphosed rocks, their high Cl contents were incorporated during contact metamorphism on a local scale in the presence of fluids enriched in Cl (F is minor/absent) released from the shallower sources, such as sediment deposits or country rocks^40,42^. Further, it is difficult to incorporate Cl and easier to incorporate F in apatite at high *P–T* conditions from a fluid containing both F and Cl^8,44^. However, high *P*–*T* experimental investigations suggest that acidic H_2_O–HCI or H_2_O–CO_2_ fluids, even with modest Cl and F concentrations, results in high Cl composition in apatite at high *P*–*T* conditions^8^. In this contribution, we provide first evidence for the presence of acidic fluids in the lower crust (~20–40 km) based on the trends in Cl and F content in naturally-occurred apatite grains (with a high Cl value of 2.95 wt%) under high-grade granulite facies metamorphic rocks (viz., metagabbro, two-pyroxene granulite and charnockite) preserved in the Nilgiri Block, southern India.

### Geological setting and apatite composition

**Figure 2:**
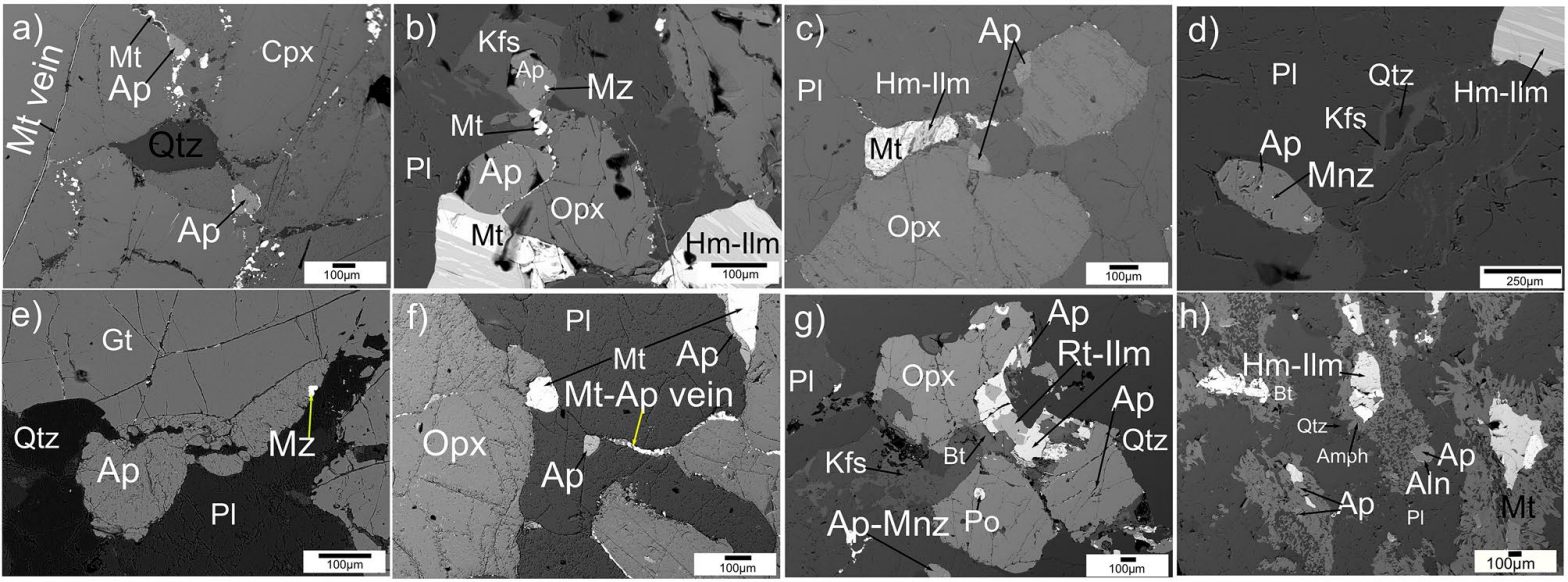
High contrast back-scattered secondary electron (BSE) imaging of phosphate-silicate-oxide-sulfide mineral textures using an Electron Probe Micro Analyzer (EPMA). In metagabbro and two-pyroxene granulite, apatite occurs in equilibrium with (**a**) clinopyroxene-magnetite-quartz, (**b**) orthopyroxene-hemo-ilmentite-Mt-feldspars, where the apatite grain is surrounded by K-feldspar veins, (**c**) orthopyroxene-hemo-ilmentite-Mt-feldspars, (**d**) hemo-ilmenite-plagioclase-K-feldspar-quartz; (**e**) garnet-plagioclase-quartz, (**f**) orthopyroxene-plagioclase-magnetite, and (**g**) in charnockite, apatite exists in equilibrium with orthopyroxene-rutile-ilmenite-biotite-feldspar-quartz; (**h**) in Hb-Bt gneiss, apatite is associated with hemo-ilmenite-magnetite-biotite-hornblende-plagioclase-quartz. *Qtz* quartz, *Plg *plagioclase, *Kfs* K-feldspar, *Opx* orthopyroxene, *Cpx* clinopyroxene, *Gt* garnet, *Amph* amphibole, *Bt* biotite, *Hm-Ilm* hematite–ilmenite exsolutions. *Mt* magnetite, *Ilm-Rt* ilmenite-rutile assemblage, *Po* pyrrhotite, *Ap* apatite, *Aln* alanite, and *Mnz* monazite. This figure is created using Inkscape version 0.92.2 (https://inkscape.org).

The Nilgiri Block mainly preserves granulite-facies metagabbro exposures in the north, two-pyroxene granulite at the central domain, charnockite in the south, and amphibolite facies hornblende (Hb)-biotite (Bt) gneiss in the west^20^ (Fig. 1a,b). Detailed field geological settings, metamorphic history, and major mineral compositions are provided in a previous study^20^, and are summarized here in Extended Data Table 1. Metagabbro samples were equilibrated at *P*–*T* conditions of ~ 1 GPa—~ 850°C, and the two-pyroxene granulite at ~ 0.8 GPa— ~ 800 °C, whereas the charnockite samples show a regional variation in pressure and temperature of ~800–750 °C and ~0.8–0.7 GPa, respectively^20^. Samples of this study (Fig. 1b; Extended Data Table 1) were selected based on the extensive regional scale petrological study^20^. The apatite grains in these rocks are euhedral to subhedral, and show no obvious preferred mineral or textural association. They occur (1) as inclusions in major minerals, (2) along their boundaries, (3) within the quartz-feldspar matrix, (4) near K-feldspar micro-veins, and (5) in association with oxide-sulphide minerals and their veins (Fig. 2). Textural features suggest that apatite coexists in equilibrium with the other minerals that were formed during peak metamorphism (Fig. 2). The apatite grains show no compositional zoning. Similar compositional homogeneity is also observed for major minerals in these samples^20^. Grain boundaries of garnet, pyroxene and plagioclase are intact (Fig. 2). Previous geochronological studies indicate that these rocks were not subject to younger thermal disturbance or alteration following peak metamorphism at ~ 2.5 Ga^13,17,19^.

An electron probe micro analyzer (EPMA) was used to measure the elemental oxides and halogen composition of the apatite in the rock samples. Analytical conditions for the EPMA measurements are provided in “Methods”. Compositions of 462 apatite grains were measured from the 34 samples selected for this study (Extended Data Table 2–6). Apatite in all four rock types studied here mainly consisted of Ca, P, F, Cl, OH with slight enrichment in Fe (0.1–0.8 wt%). Highest Cl content of 2.95 wt% (sample no. 105 on Fig. 1b, Extended Data Table 3), 0.52 wt%, (sample no. 84 on Fig. 1b, Extended Data Table 4) 1.27 wt% (sample no. 42 on Fig. 1b, Extended Data Table 5) and 0.11 wt% (sample NIL27-14, Extended Data Table 6), were obtained from the metagabbro, two-pyroxene granulite, charnockite, and Hb-Bt gneiss samples, respectively. In the highest Cl bearing metagabbro sample (no. 105 on Fig. 1b), out of total 23 grains, ten grains have mean Cl content of 2.75 wt% and thirteen grains have mean Cl content of 1.41 wt%. Another high Cl bearing metagabbro sample (no. 106 on Fig. 1b) has total seven grains with mean Cl content of 1.03 wt%. The mean Cl content variation in apatite (5–30 apatite grains from each sample) was found to be 0.1 (n = 6, no. 102 on Fig. 1b)–2.75 (n = 10, no. 105 on Fig. 1b) wt% in metagabbro, 0.1 (n = 16, no. 85 on Fig. 1b)–0.2 (n = 2, no. 84 on Fig. 1b) wt% in two-pyroxene granulite, 0–0.49 (n = 13, no. 42 on Fig. 1b) wt% in charnockite, and 0–0.08 (n = 4, NIL27-14) wt% in Hb-Bt gneiss (Extended Data Table 2). There is no preferred textural association with respect to these variations (Fig. 2).

### Characteristics of acidic metamorphic conditions

**Figure 3:**
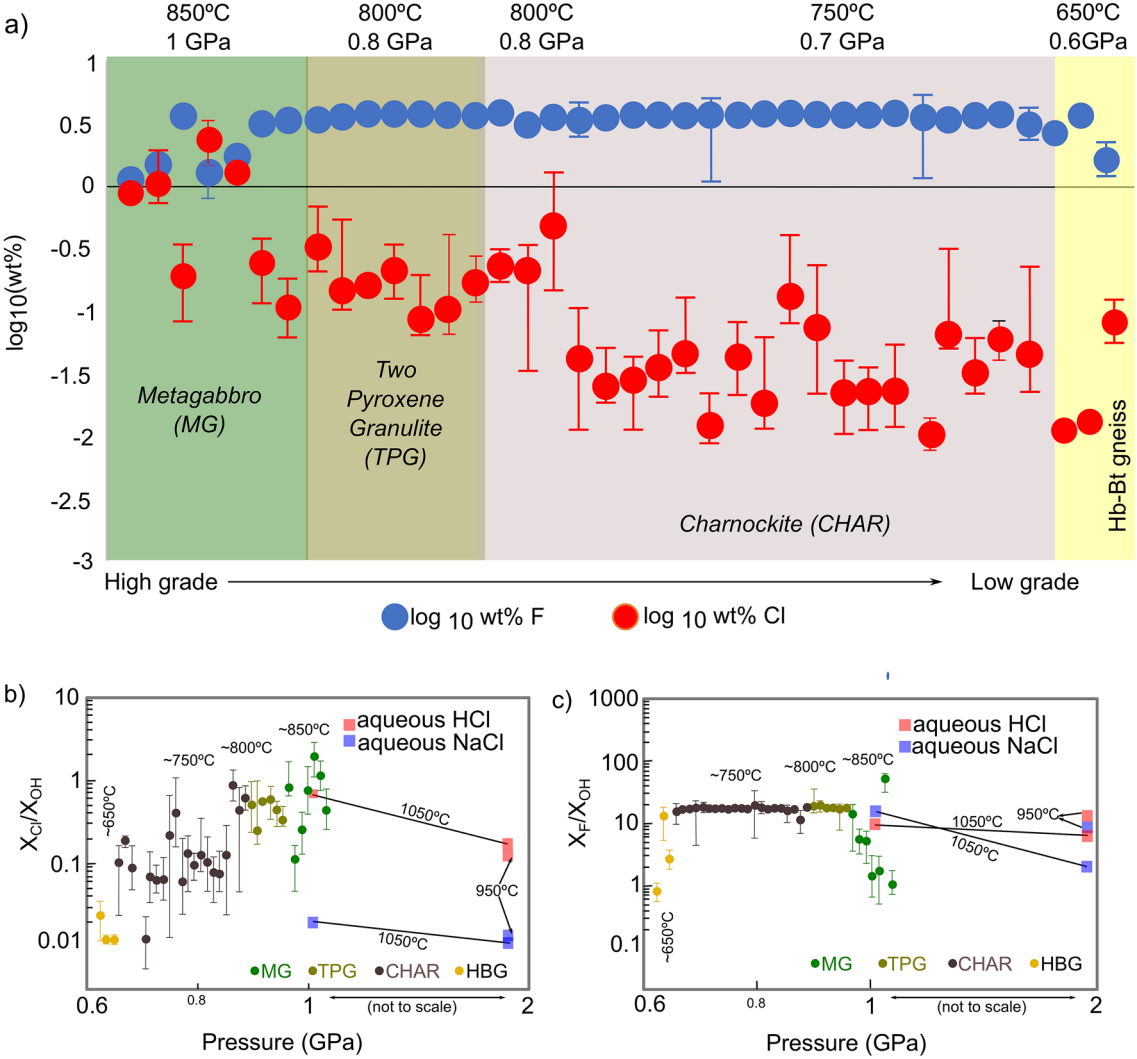
Regional distribution of Cl, F in apatite, and comparison of apatite composition with previous experimental results. Plots representing (**a**) regional distribution of log_10_ Cl wt % and log_10_ F wt% in apatite. Each data point represents the average Cl and F wt% in apatite grains in each sample (vertical bar-lowest and highest Cl or F content in apatite grains in each sample); (**b**) Mean X_Cl_/X_OH_ values with vertical bar representing lowest and highest values in each sample. This data is compared with X_Cl_/X_OH_ apatite data from experiments (the experimental data is normalized to 1 wt% Cl in the fluid) involving apatite-aqueous HCl and apatite-aqueous NaCl from Ref.^8^; (**c**) Mean X_F_/X_OH_ values with vertical bar representing lowest and highest values in each sample. The data are compared with X_F_/X_OH_ apatite data from experiments (the experimental data is normalized to 0.1 wt% F in the fluid) involving apatite-aqueous HCl and apatite-aqueous NaCl from Ref.^8^. *CHAR* charnockite samples, *TPG* two-pyroxene granulite samples, *MG* metagabbro samples, *HBG* hornblende-biotite gneiss samples. This figure is created using MS Excel version 16.16.27 (https://www.microsoft.com/en-us/microsoft-365/excel) and modified using Inkscape version 0.92.2 (https://inkscape.org).

Observed Cl content in the apatite shows a decreasing trend with respect to the metamorphic grade (regional variation trend; Fig. 3a). Among these, three charnockite samples, which formed at a higher *P*–*T* conditions^20^ (sample no. 6, 8, 42 on Fig. 1b) and have high Cl content, are shown separately from those bearing low Cl content (sample no. 12–61 except 42 on Fig. 1b). F content in the apatite, on the other hand, was found to be low in metagabbro, although it displays a constant value of ~ 3 wt% in two-pyroxene granulite, charnockite, and Hb-Bt gneiss (Fig. 3a).

Partitioning of F and Cl from the co-existing fluids into apatite has been demonstrated to be highly sensitive to the fluid composition, and the prevailing *P*–*T* conditions^8,41–44^. In the Nilgiri samples, the metagabbro, two-pyroxene granulite and three high-grade high-Cl-bearing charnockites show X_Cl_/X_OH_ in the range of 0.01–2.5 and X_F_/X_OH_ in the range of 1–20. Based on an earlier experimental investigation^8^, in a piston-cylinder apparatus that equilibrated natural fluorapatite with aqueous HCI, NaCl, NaOH, Na_2_CO_3_ and CO_2_–H_2_O mixtures at 1–2.0 GPa and 950–1050 °C, such high X_Cl_/X_OH_ values, in Cl- and F-bearing apatite, indicate apatite equilibrating in acidic fluids (e.g., HCl with ~ 15 wt% Cl, or ~ 8 wt% Cl in CO_2_–H_2_O fluids—considering the linear relationship observed in experiments^8^). At these *P*–*T* conditions, neutral pH is ~ 4 because of the increase in dissociation constant^9^ to ~ 10^–8^, and therefore the acidic nature of the fluid co-existing with apatite is relative to this neutral pH. Abundance of hemo-ilmenite-magnetite-pyrite in the metagabbro and two-pyroxene granulite samples show that these rocks are relatively oxidized^20^, and also could be considered as supportive evidence for the acidic environment during peak metamorphism. Apatite equilibration in aqueous NaCl solutions with ~ 50 wt% Cl at 1GPa (water activity: 0.4 at 1 GPa^35,36^) can only result in X_Cl_/X_OH_ in the range of 0.5–0.6 (considering the linear relationship observed in experiments with aqueous NaCl fluids at 1050 °C and 2 GPa^8^). Additionally, rock-buffered experimental results^44^ of subduction zone lithologies also confirm the experimental results^8^ that suggest the acidic nature of evolving fluids (containing both Cl and F) in  that incorporating high Cl content in apatite cannot be excluded. Such studies strongly suggest that it is difficult to incorporate high Cl content in apatite equilibrating with a fluid containing both F and Cl under normal or high pH conditions at high pressure and high temperature. Therefore, it is reasonable to infer the presence of an acidic environment, due to interaction of fluids containing high-Cl with rocks, during the metamorphism of these rock samples. Thus, comparing our results with observations from an experimental study^8^ (using both aqueous HCl and aqueous NaCl) shows that X_Cl or F_/X_OH_ values in most of our samples formed in an acidic environment (Fig. 3b,c).

According to high *P*–*T* experiments (950–1050 °C and 1–2 GPa)^8^, if we assume constant Cl, F content in the acidic fluid, the X_Cl_/X_OH_ of the apatite will rise with temperature at constant pressure, but drop with pressure at constant temperature. In our samples, X_Cl_/X_OH_ of apatite increases with pressure and temperature (Fig. 3b). In acidic fluids containing F, an increase in temperature has been demonstrated to significantly decrease X_F_/X_OH_ content in apatite^8^ (Fig. 3c). In our samples X_F_/X_OH_ decrease with pressure and temperature from two-pyroxene granulite to metagabbro. In contrast, X_F_/X_OH_ in charnockite samples show a constant trend, and amphibolite facies Hb-Bt gneiss samples show a decreasing trend.

An apparent increase in the equilibrium constant (K), [considering ideal conditions^39^, (K_Cl/OH_ = (f_HCI_ × a_OH-ap_)/(f_H2O_ × a_Cl-ap_)) and (K_F/OH_ = (f_HF_ × a_OH-ap_)/(f_H2O_ × a_F-ap_)), for the exchange reactions Ca_5_(PO_4_)_3_Cl + H_2_O = Ca_5_(PO_4_)_3_OH + HCl and Ca_5_(PO_4_)_3_F + H_2_O = Ca_5_(PO_4_)_3_OH + HF respectively] with respect to increasing pressure and temperature can be expected at the granulite facies conditions based on data available from previous low *P*–*T* studies^30,31^ (extrapolating the values of K at low *P*–*T* conditions (0.1–0.4 GPa, 400–700 °C)^41,42^ to granulite facies conditions). We also need to bear in mind the low f_H2O_ nature of these fluids in producing anhydrous minerals in these rocks. Assuming these conditions, we estimated the variation of fugacity of the fluid components (f_HCl_/f_H2O_ and f_HF_/f_H2O_). Assuming a constant f_HCl or HF_/f_H2O,_ as the *P*–*T* conditions increase, we expect a decreasing trend in X_Cl/F_/X_OH_. Contrary to expectations, X_Cl_/X_OH_ increase in our samples, and X_F_/X_OH_ remains constant from charnockite to two-pyroxene granulite samples and decrease from two-pyroxene granulite to metagabbro samples. This might be due to extremely high f_HCl_/f_H2O_ in metagabbro, two-pyroxene granulite and the three high-Cl-bearing charnockite samples formed at high-grade conditions. However, in the remaining charnockite samples and amphibolite facies Hb-Bt gneiss samples, f_HCl_/f_H2O_ shows a gradual decrease, with respect to decrease in *P*–*T*. The value of f_HF_/f_H2O_ seems to be extremely low in metagabbro, and gradually increase from two-pyroxene granulite to charnockite samples. In summary, the comparison of our observations with both high and low *P*–*T* experimental data suggest that a change in *P*, *T* or the equilibrium constant (based on available data from previous studies) are not the only cause for the observed regional trend.

Similar trends with Cl (relatively low) and F content in apatite have been observed along the Krishnagiri to Salem traverse in the Shevaroy Hills, adjoining the Nilgiri Block^32^. In this case, the two-pyroxene granulite (named as clinopyroxene-orthopyroxene zone) has the highest Cl content (~ 0.5 wt%) in apatite. Relatively lesser Cl content has been detected in charnockite and amphibolite facies regions (Hb-Bt gneiss).

High *P*–*T* acidic conditions (observed in metagabbro, two-pyroxene granulite and the three high-grade charnockite samples) favor partitioning of more F into fluids relative to Cl during fluid-mineral interactions^32^. Relationship between X_Cl_/X_OH_ in apatite and wt% Cl in fluids observed in experiments also suggests that a change in fluid composition has a significant effect on the Cl content in apatite^8^. A gradual lowering of Cl in apatite below 800 °C and 0.8 GPa can be seen in our data (Fig. 3). Thus, possibly below 800 °C and 0.8 GPa, the acidic fluid is enriched in F compared to Cl and its composition may gradually shift from high Cl to high F. Another possibility is that Cl is shielded by metal complexes formed during speciation below 800 °C and 0.8 GPa.

### Nature of fluids and their implications

Abundant CO_2_ fluid inclusions in minerals are reported from the major rock types in the Nilgiri and Shevaroy high-grade terranes^20–23^. No salt composition is detected in these inclusions. Comparing these results with our observations and previous experimental results^8,43^ suggests that Cl and F bearing C–O–H fluids could have been more prevalent agents of metamorphism in the lower crust than aqueous NaCl (saline fluids/brine). Moreover, textures like apatite surrounded by K-feldspar veins as well as oxidation textures indicate a convergence of mineralogical and textural features with an acidic environment created by fluids. However, the capacity of C–O–H fluids to cause metamorphic recrystallization was considered low due to the non-polar nature of CO_2,_ and relatively less solubility of minerals in them (compared to saline fluids at low salt concentration)^35,36^. Recent thermodynamic calculations at 900 °C and 5.0 GPa show a pH drop during irreversible C–O–H fluid-rock reactions, suggesting that at lower pH, C–O–H fluids could be a good agent of metamorphic recrystallization^45^. Therefore, we propose that fluid–rock reactions in the granulite facies conditions might have reduced the pH of C–O–H-Cl–F fluids, causing high Cl content in apatite, and formation of extensive regional anhydrous granulites.

The source of these high—Cl, F—bearing fluids at a depth of ~20–40 km is currently unknown, although it is possible to speculate different possible scenarios. Cathodoluminescence (CL) images and U–Pb data of zircon grains from both mafic and felsic rock types, together with field and textural evidence of mineral assemblages in this terrane clearly show that they were extensively recrystallized during metamorphism after their magmatic crystallization^12–14,17–20^. Since these rocks were not thermally perturbed after their peak granulite facies metamorphism, any possibility of contact metamorphism in the presence of fluids released from shallower sources^40,42^, such as sediment deposits or country rocks, can be excluded. Also, no shallow sedimentary contacts are reported in the vicinity of these rocks. Another possible source is the residual Cl and F enriched fluids left after the partial melting of protoliths at high-grade conditions^35^. However, there is no evidence for formation of crustal-scale migmatite during a partial melting of these outcrops^20^. Further, the charnockite or two-pyroxene granulite might not represent a residual phase after partial melting because they are enriched in incompatible elements and LREE^14^. In addition, they cannot be considered as a melt phase from partial melting of the mafic granulite/metagabbro, because of their concurrent magmatic and metamorphic ages^14,18,19^. If the two-pyroxene granulite and charnockite were partial melts of metagabbro, then the metamorphic age of metagabbro should have been similar to the magmatic (core) age of charnockite and two pyroxene granulite. Previous geochemical studies using compatible elements shows that, these rocks (both felsic and mafic) could have formed by differentiation of underplated mafic magma produced in an arc magmatic setting^14,20^. Solid state recrystallization of these rocks during metamorphism could have been assisted by fluids infiltrating from deep or within. Three likely scenarios can be considered for the origin of fluids during metamorphism. The first possibility is that the fluids were auto-generated in the rocks during heating and dehydration reactions within the rock. Second possibility is that they were mantle derived^46,47^. A third scenario is that the fluids might have originated from crystallizing mafic magma stalled beneath the lower crust (e.g. basaltic underplating) that assisted the metamorphic process during collision with the Dharwar Craton at ca. 2.45–2.5 Ga. The solubility of Cl and F in mafic magma is markedly high^48^. Therefore, during the initial stages of underplated magma crystallization, relatively higher Cl and F may be expected to preferentially partition into the fluids relative to the solids^48^. Such acidic fluids (C–O–H–Cl–F or H–Cl–F) infiltrating into the crustal column above could participate in metamorphism. The Cl and F content in such underplated mafic magma could be mantle-derived or due to mixing of Cl-rich fluids released from subducting lithologies such as oceanic crust or subducted sediments in the mantle wedge during formation of mafic magma^49–51^. Irrespective of the uncertainty in their sources, fluid-rock interactions at granulite facies conditions could result in an acidic environment (drop in pH) as is evident from the results reported in this study.

### Future directions

Acidic fluids have high potential in causing mass transfer of important elements used for geochemical modeling of petrogenesis of rocks, and also of economically important elements regionally^8–11^. Further experimental and theoretical estimations are needed to establish the link between the fugacity of HCl/HF in minerals with that of evolving fluid pH in a rock-buffered system. If such data could be generated then it would be possible to deduce a solution model for OH–F–Cl apatite or to estimate f_HF_ and f_HCl_ to compute the speciation of C–O–H solvent at granulite facies conditions. Improved understanding about the speciation properties and role of acidic fluids in lower crustal processes, such as subduction, arc magmatism, formation of economic mineral deposits, etc., can be sought through further field, analytical, experimental, and thermodynamic studies. This would allow us to improve the uncertainties in modelling the long-term geochemical evolution of Earth’s lithosphere and its environment.

## Methods

Electron microprobe (EPMA) analyses of apatite grains given in Extended Data Tables 2–6 were conducted on a 5 channel—JEOL JXA-8100 Superprobe, Electron Probe Micro Analyzer (EPMA), housed at the Department of Earth System Sciences, Yonsei University, Seoul, South Korea. Analytical conditions are accelerating voltage of 20 kV; beam current of 15 nA; counting time of 10 s for F, Cl, Ca, Na, Al, Si, P, Mn and Fe and 50 s for trace elements; an electron beam spot size of 15 μm^52,53^. F and Cl were kept first in the analysis list due to their volatile nature^52,53^. F X-ray excitation tends to increase with time during EMPA, therefore elongated apatite grains were chosen for analysis^52,53^ (Fig. 2). This has been done based on the assumption that grains are oriented parallel to [0001] and would minimize the increase in F X-ray excitation with time. Standards (F-apatite, Cl-apatite, and REE-Y-PO_4_) supplied by Prof. Daniel E. Harlov, GFZ Potsdam, Germany were used for calibration of F, Cl, Ca, P, and REE’s. During standardization and analysis, TAP crystal (L value ~ 199.2 mm) is used to diffract F X-rays and PET crystal is used for Cl (L value ~ 151.8 mm). Standardization and analysis (including interference correction) for all other elements were done based on Ref.^52^. Natural and synthetic silicates and oxides supplied by JEOL and ASTIMEX Standards Ltd., Canada, were used for calibration of Na, Al, Si, Mn and Fe. Relative errors in EMPA are estimated to be < 1% at the > 10 wt% level, 5–10% at the 1–10 wt% level, 10–20% at the 0.2 to 1 wt % level, and 20–40% at the < 0.1 wt% level^26^. Detection limits were ~ 500 ppm for (Y + REE) and ~ 100 ppm for the remaining elements. The data were reduced using the ZAF correction procedures supplied by JEOL. For most samples, we have chosen an average of between 5 and 35 grains. Mineral recalculation (Extended Data Table 2–6) applied the method used in Ref.^32^. Mole fractions of OH in apatite are calculated assuming the halogen site is filled with F, Cl, and OH (X_OH_Ap = 1 − X_F_Ap − X_Cl_Ap).

## Supplementary Information

Supplementary Information.
