# Supplementary material for: Acidic fluids in the Earth’s lower crust
Source: Sci Rep. 2021 Oct 27;11:21146. doi: 10.1038/s41598-021-00719-3 (PMC8551242; doi:10.1038/s41598-021-00719-3)
Supplement: Supplementary file 1 — Supplementary Information. [file 41598_2021_719_MOESM1_ESM.pdf]

Extended Data Table 1: Summary of sample locations, rock types and mineralogy

Serial No.	No. on map	Sample Label	Rock Type	Latitude	Longitude	Qtz	Plg	Kfs	Opx	Cpx	Gt	Amph	Bt	Hm-Ilm	Ilm	Mt	Ilm-Rt	Po	Py	Po-Cp-Py	Py-Mt	Oxide rims Opx, Cpx, Hb	Sulphide Vein	Graphite
1	6	NIL25-5	Charnockite	11.4627	76.6107	X	X	X	X		X		X		X					X				
2	8	NIL25-11	Charnockite	11.3889	76.7299	X	X	X	X		X		X				X			X				
3	12	NIL25-17	Charnockite	11.3590	76.7829	X	X	X	X		X		X		X		X			X				
4	18	NIL31-3	Charnockite	11.4108	76.7769	X	X	X	X		X		X		X		X	X		X			X	
5	23	NIL31-12	Charnockite	11.4162	76.8194	X	X	X	X		X		X		X		X			X			X	
6	27	NIL31-16	Charnockite	11.4327	76.8204	X	X	X	X		X		X					X						X
7	29	NIL31-18	Charnockite	11.4231	76.8251	X	X	X	X		X		X				X	X						
8	37	NIL2-8	Charnockite	11.3643	76.9063	X	X	X	X		X		X		X		X	X		X				
9	40	NIL2-11	Charnockite	11.3599	76.9523	X	X	X	X		X		X		X			X		X				
10	41	NIL2-12	Charnockite	11.3565	76.9437	X	X	X	X		X									X				
11	42	NIL2-18	Charnockite	11.3434	76.8498	X	X	X	X		X		X		X		X	X					X	
12	45	NIL2-21	Charnockite	11.3357	76.8172	X	X	X	X		X		X					X		X				
13	54	NIL3-9	Charnockite	11.3594	76.8073	X	X	X	X		X		X		X		X			X				X
14	57	NIL3-16	Charnockite	11.2931	76.6693	X	X	X	X		X		X		X		X			X +Ilm				X
15		NIL 3-17	Charnockite	11.2825	76.6556																			
16	58	NIL3-19	Charnockite	11.2908	76.6465	X	X	X	X		X		X		X		X			X+ Ilm			X	
17	59	NIL3-20	Charnockite	11.3185	76.6427	X	X	X	X		X		X		X					X				
18	61	NIL26-12-24	Charnockite	11.2495	76.6651	X	X	X	X		X		X		X		X			X				
19	62	NIL30-15	Charnockite	11.4843	76.9085	X	X	X	X		X		X					X						
20	69	NIL24-6	Two pyroxene granulite	11.4614	76.7501	X	X	X	X	X		X		X		X								
21	73	NIL24-10	Two pyroxene granulite	11.4824	76.7700	X	X	X	X	X		X		X+Mt		X		X			X	X	X	
22	80	NIL29-1	Two pyroxene granulite	11.4948	76.5143	X	X	X	X	X		X		X		X						X		
23	84	NIL29-8	Two pyroxene granulite	11.4816	76.5475	X	X	X	X	X		X	X	X	X	X				X				
24	85	NIL29-10	Two pyroxene granulite	11.4623	76.6012	X	X	X	X	X	X			X +Mt	X	X		X			X	X		
25	98	NIL2-6	Two pyroxene granulite	11.3805	76.8980				X	X				X		X		X			X	X		
26	102	NIL10-84	Mafic granulite/ Metagabbro	11.3900	76.7200	Few grains	X	X	X	X	X	X		X		X					X	X		
27	103	NIL23-5	Mafic granulite/ Metagabbro	11.5306	76.7030	Few grains	X	X	X	X	X	X		X		X					X	X		
28	104	NIL30-11	Mafic granulite/ Metagabbro	11.4466	76.9241	Few grains	X	X	X	X	X	X		X		X					X	X		
29	105	NIL25-4	Mafic granulite/ Metagabbro	11.4480	76.6230	Few grains	X	X		X	X	X		X							X			
30	106	NIL16-1	Mafic granulite/ Metagabbro	11.5700	76.6500	Few grains	X	X		X	X	X		X		X					X			
31	109	NIL23-10	Mafic granulite/ Metagabbro	11.4969	76.6893	Few grains	X	X	X	X	X	X		X		X								
32	132	NIL27-1	Hb-Bt gneiss	11.6571	76.1921	X	X	X				X	X											
33	133	NIL27-4	Hb-Bt gneiss	11.6378	76.0943	X	X	X				X	X		X	X								
34	138	NIL27-14	Hb-Bt gneiss	11.6415	75.9714	X	X	X				X	X											

Qtz-Quartz; Plg- Plagioclase; Kfs- K-feldspar; Opx- Orthopyroxene; Cpx-Clinopyroxene; Gt-Garnet; Amph-Amphibole; Bt-Biotite;
Hm-ilm- Hematite-ilmenite association; Ilm- Ilmenite; Mt-Magnetite; Rt-Rutile; Po-Pyrrhotite; Py-Pyrite; Cp-Chalcopyrite; Ox-Oxidation; Hb-Hornblende

Extended Data Table 2: Mean apatite F and Cl compositions from all samples

No.	Rock type	No. on map	No. of grains analyzed	Avg. F (wt%)	*low (wt%)*	*high (wt%)*	Avg. Cl (wt%)	*low (wt%)*	*high (wt%)*	Avg. X_F/X_{OH}	*low*	*high*	Avg. X_{Cl}/X_{OH}	*low*	*high*
1	MG	*106*	*7*	1.57	*1.37*	*1.88*	1.03	*0.73*	*1.41*	1.05	*0.76*	*1.82*	0.37	*0.22*	*0.73*
2	MG	*103*	*4*	3.75	*3.49*	*3.92*	0.16	*0.08*	*0.31*	51.54	*33.09*	*61.95*	1.01	*0.67*	*1.56*
3a	MG	*105*	*10*	1.35	*0.78*	*1.80*	2.75	*2.63*	*2.95*	1.78	*0.53*	*3.05*	1.85	*0.95*	*2.46*
3b	MG	*105*	*13*	1.65	*1.21*	*2.20*	1.41	*1.07*	*2.04*	1.45	*0.65*	*2.97*	0.67	*0.35*	*1.30*
4	MG	109	11	3.07	*2.56*	*3.21*	0.24	*0.11*	*0.35*	5.32	*2.43*	*7.51*	0.22	*0.11*	*0.36*
5	MG	102	6	3.25	*2.84*	*3.42*	0.10	*0.06*	*0.16*	5.55	*3.37*	*7.92*	0.09	*0.04*	*0.14*
6	MG	104	13	3.46	*3.08*	*3.79*	0.33	*0.20*	*0.65*	13.38	*3.65*	*20.23*	0.75	*0.21*	*1.62*
7	TPG	*80*	*16*	3.63	*3.47*	*3.78*	0.15	*0.10*	*0.52*	17.20	*16.45*	*17.90*	0.32	*0.24*	*0.41*
8	TPG	*73*	*5*	3.66	*3.22*	*3.78*	0.16	*0.15*	*0.18*	17.48	*8.04*	*19.93*	0.40	*0.24*	*0.49*
9	TPG	*84*	*2*	3.67	*3.61*	*3.74*	0.21	*0.12*	*0.29*	17.42	*17.10*	*17.74*	0.52	*0.31*	*0.74*
10	TPG	98	1	3.52	*3.52*	*3.52*	0.18	*0.18*	*0.18*	18.53	*18.53*	*18.53*	0.49	*0.49*	*0.49*
11	TPG	85	16	3.64	*3.48*	*3.78*	0.09	*0.06*	*0.35*	17.27	*16.48*	*17.92*	0.22	*0.15*	*0.90*
12	TPG	69	20	3.62	*3.20*	*3.86*	0.17	*0.13*	*0.26*	18.72	*16.40*	*36.03*	0.45	*0.21*	*0.87*
13	CHAR	6	9	3.70	*3.51*	*3.85*	0.23	*0.16*	*0.32*	17.52	*16.65*	*18.27*	0.58	*0.40*	*0.80*
14	CHAR	8	7	3.13	*2.98*	*3.27*	0.20	*0.03*	*0.32*	10.73	*7.06*	*15.48*	0.39	*0.04*	*0.77*
15a	CHAR	42	13	3.60	*3.32*	*3.80*	0.49	*0.14*	*1.27*	16.46	*15.74*	*17.43*	0.78	*0.52*	*1.19*
15b	CHAR	42	12	3.48	*2.23*	*3.71*	0.04	*0.01*	*0.10*	16.04	*10.56*	*17.58*	0.10	*0.02*	*0.25*
16	CHAR	27	15	3.67	*3.48*	*3.79*	0.03	*0.02*	*0.05*	17.38	*16.48*	*17.98*	0.06	*0.04*	*0.12*
17	CHAR	29	14	3.67	*3.45*	*3.82*	0.03	*0.01*	*0.04*	17.41	*16.35*	*18.10*	0.07	*0.03*	*0.10*
18	CHAR	18	9	3.62	*3.51*	*3.72*	0.04	*0.02*	*0.07*	17.14	*16.40*	*17.64*	0.09	*0.04*	*0.18*
19	CHAR	23	9	3.62	*2.95*	*3.99*	0.04	*0.03*	*0.12*	17.17	*16.29*	*19.27*	0.11	*0.07*	*0.32*
20	CHAR	59	29	3.62	*3.15*	*3.88*	0.01	*0.00*	*0.02*	18.76	*5.86*	*34.99*	0.08	*0.00*	*0.07*
21	CHAR	12	14	3.65	*3.44*	*3.79*	0.04	*0.02*	*0.08*	17.32	*16.30*	*17.99*	0.11	*0.04*	*0.19*
22	CHAR	54	12	3.71	*3.45*	*3.83*	0.02	*0.01*	*0.06*	17.74	*16.34*	*20.11*	0.05	*0.02*	*0.17*
23a	CHAR	57	6	3.73	*3.58*	*3.93*	0.14	*0.08*	*0.40*	17.69	*16.95*	*18.64*	0.35	*0.15*	*1.01*
23b	CHAR	57	9	3.56	*3.50*	*3.65*	0.08	*0.02*	*0.23*	16.89	*16.61*	*17.31*	0.19	*0.04*	*0.59*
24	CHAR	3–17	9	3.50	*3.41*	*3.60*	0.02	*0.01*	*0.04*	16.60	*17.07*	*16.17*	0.06	*0.03*	*0.10*
25	CHAR	58	18	3.63	*3.32*	*3.86*	0.01	*0.01*	*0.03*	17.38	*15.75*	*18.30*	0.06	*0.03*	*0.08*
26	CHAR	62	14	3.58	*3.38*	*3.73*	0.02	*0.01*	*0.05*	16.95	*16.00*	*17.68*	0.06	*0.02*	*0.12*
27	CHAR	37	16	3.92	*3.42*	*4.20*	0.00	*0.00*	*0.01*	18.58	*19.89*	*16.21*	0.01	*0.00*	*0.02*
28	CHAR	45	35	3.57	*2.98*	*4.51*	0.06	*0.00*	*0.30*	17.43	*4.72*	*21.40*	0.04	*0.00*	*0.48*
29	CHAR	61	10	3.62	*3.28*	*3.79*	0.03	*0.02*	*0.06*	17.14	*15.57*	*17.99*	0.08	*0.04*	*0.14*
30	CHAR	40	18	3.70	*3.63*	*3.78*	0.06	*0.04*	*0.07*	16.81	*16.34*	*17.27*	0.16	*0.14*	*0.17*
31	CHAR	41	35	3.31	*2.03*	*3.77*	0.05	*0.02*	*0.23*	14.88	*9.62*	*17.78*	0.09	*0.02*	*0.10*
32	HBG	27–1	2	2.69	*2.49*	*2.90*	0.01	*0.01*	*0.01*	2.70	*3.43*	*1.96*	0.01	*0.01*	*0.01*
33	HBG	27–4	5	3.58	*3.23*	*3.70*	0.00	*0.00*	*0.01*	12.83	*5.29*	*18.12*	0.00	*0.00*	*0.01*
34	HBG	27–14	4	1.78	*1.32*	*2.07*	0.08	*0.06*	*0.11*	0.87	*0.52*	*1.09*	0.02	*0.01*	*0.03*

***MG-metagabbro; TPG-two pyroxene granulite; CHAR-charnockite; HBG-hornblende-biotite gneiss**

Extended Data Table 3: Apatite compositions (wt%) in metagabbro samples.

Page 1								
Metagabbro sample	1							*1*
No. on Map	106							*106*
No of grains analysed	1	2	3	4	5	6	7	*7*
Sample no	NIL16-1	NIL16-1	NIL16-1	NIL16-1	NIL16-1	NIL16-1	NIL16-1	NIL16-1
								Mean
P_2O_5	40.06	40.64	41.49	41.79	41.71	40.13	39.39	*40.74*
SiO_2	0.09	0.17	0.05	0.16	0.17	0.18	0.21	*0.15*
Al_2O_3	0.07	0.07	0.25	0.02	0.03	0.08	0.05	*0.08*
SO_2	0.18	0.26	0.05	0.31	0.17	0.08	0.31	*0.19*
Y_2O_3	0.00	0.00	0.00	0.00	0.00	0.00	0.00	*0.00*
La_2O_3	0.02	0.07	0.00	0.00	0.06	0.01	0.00	*0.02*
Ce_2O_3	0.11	0.06	0.00	0.11	0.08	0.04	0.09	*0.07*
Pr_2O_3	0.01	0.00	0.00	0.00	0.07	0.07	0.04	*0.03*
Nd_2O_3	0.01	0.07	0.02	0.08	0.02	0.07	0.12	*0.06*
Sm_2O_3	0.00	0.00	0.00	0.00	0.00	0.00	0.00	*0.00*
Gd_2O_3	0.00	0.00	0.00	0.00	0.00	0.00	0.00	*0.00*
Dy_2O_3	0.00	0.00	0.00	0.00	0.00	0.00	0.00	*0.00*
CaO	54.04	54.48	55.55	55.87	55.62	54.41	54.61	*54.94*
FeO	0.23	0.54	0.49	0.93	0.62	0.12	0.77	*0.53*
MnO	0.04	0.00	0.01	0.06	0.00	0.01	0.00	*0.02*
SrO	0.00	0.00	0.00	0.00	0.00	0.00	0.00	*0.00*
Na_2O	0.11	0.05	0.00	0.09	0.05	0.00	0.11	*0.06*
F	1.88	1.40	1.42	1.37	1.71	1.44	1.80	*1.57*
Cl	1.41	1.17	1.10	1.10	0.91	0.76	0.73	*1.03*
H_2O	0.49	0.79	0.82	0.85	0.74	0.87	0.70	*0.75*
(F+Cl+OH)	3.78	3.36	3.34	3.32	3.36	3.07	3.23	*3.35*
sum	98.74	99.76	101.24	102.74	101.94	98.28	98.93	*100.23*
O=(F+Cl)	1.11	0.85	0.85	0.83	0.93	0.78	0.92	*0.89*
total	97.63	98.91	100.40	101.91	101.01	97.50	98.00	*99.34*
(Y+REE)	0.14	0.20	0.02	0.19	0.22	0.19	0.25	*0.17*
P	2.95	2.95	2.96	2.94	2.96	2.95	2.89	*2.94*
Si	0.01	0.01	0.00	0.01	0.01	0.02	0.02	*0.01*
Al	0.01	0.01	0.02	0.00	0.00	0.01	0.01	*0.01*
S	0.02	0.03	0.01	0.04	0.02	0.01	0.04	*0.02*
Y	0.00	0.00	0.00	0.00	0.00	0.00	0.00	*0.00*
La	0.00	0.00	0.00	0.00	0.00	0.00	0.00	*0.00*
Ce	0.00	0.00	0.00	0.00	0.00	0.00	0.00	*0.00*
Pr	0.00	0.00	0.00	0.00	0.00	0.00	0.00	*0.00*
Nd	0.00	0.00	0.00	0.00	0.00	0.00	0.00	*0.00*
Sm	0.00	0.00	0.00	0.00	0.00	0.00	0.00	*0.00*
Gd	0.00	0.00	0.00	0.00	0.00	0.00	0.00	*0.00*
Dy	0.00	0.00	0.00	0.00	0.00	0.00	0.00	*0.00*
Ca	5.03	5.00	5.01	4.98	4.99	5.05	5.08	*5.02*
Fe	0.03	0.08	0.07	0.13	0.09	0.02	0.11	*0.08*
Mn	0.01	0.00	0.00	0.01	0.00	0.00	0.00	*0.00*
Sr	0.00	0.00	0.00	0.00	0.00	0.00	0.00	*0.00*
Na	0.02	0.01	0.00	0.01	0.01	0.00	0.02	*0.01*
F	0.52	0.38	0.38	0.36	0.45	0.39	0.49	*0.43*
Cl	0.21	0.17	0.16	0.16	0.13	0.11	0.11	*0.15*
OH	0.28	0.45	0.46	0.47	0.41	0.50	0.41	*0.43*
Total	1.01	1.00	0.99	0.99	0.99	1.01	1.01	*1.00*
XF/XOH	1.82	0.84	0.83	0.76	1.10	0.78	1.22	*1.05*
XCl/XOH	0.73	0.38	0.34	0.33	0.32	0.22	0.27	*0.37*

Page 2					
Metagabbro sample	2				*2*
No. on Map	103				*103*
No of grains analysed	1	2	3	4	*4*
Sample no	NIL23-5	NIL23-5	NIL23-5	NIL23-5	NIL23-5
					Mean
P_2O_5	41.55	40.62	42.38	42.01	*41.64*
SiO_2	0.23	0.22	0.09	0.18	*0.18*
Al_2O_3	0.00	0.00	0.00	0.02	*0.01*
SO_2	0.01	0.13	0.03	0.03	*0.05*
Y_2O_3	0.00	0.02	0.00	0.00	*0.00*
La_2O_3	0.07	0.14	0.02	0.06	*0.07*
Ce_2O_3	0.21	0.32	0.14	0.25	*0.23*
Pr_2O_3	0.11	0.04	0.00	0.00	*0.04*
Nd_2O_3	0.08	0.02	0.13	0.09	*0.08*
Sm_2O_3	0.00	0.00	0.00	0.00	*0.00*
Gd_2O_3	0.00	0.00	0.00	0.00	*0.00*
Dy_2O_3	0.00	0.00	0.00	0.00	*0.00*
CaO	55.23	54.95	55.50	55.74	*55.36*
FeO	0.33	0.63	0.31	0.60	*0.47*
MnO	0.00	0.06	0.00	0.07	*0.03*
SrO	0.00	0.00	0.02	0.00	*0.01*
Na_2O	0.00	0.01	0.00	0.00	*0.00*
F	3.49	3.82	3.77	3.92	*3.75*
Cl	0.31	0.17	0.09	0.08	*0.16*
H_2O	0.05		0.03	0.03	*0.01*
(F+Cl+OH)	3.85	3.99	3.89	4.03	*3.94*
sum	101.68	101.15	102.52	103.08	*102.10*
O=(F+Cl)	1.54	1.65	1.61	1.67	*1.62*
total	100.14	99.50	100.91	101.41	*100.49*
(Y+REE)	0.48	0.54	0.29	0.41	*0.43*
P	2.97	2.93	2.99	2.96	*2.96*
Si	0.02	0.02	0.01	0.01	*0.02*
Al	0.00	0.00	0.00	0.00	*0.00*
S	0.00	0.02	0.00	0.00	*3.01*
Y	0.00	0.00	0.00	0.00	*0.00*
La	0.00	0.00	0.00	0.00	*0.00*
Ce	0.01	0.01	0.00	0.01	*0.01*
Pr	0.00	0.00	0.00	0.00	*0.00*
Nd	0.00	0.00	0.00	0.00	*0.00*
Sm	0.00	0.00	0.00	0.00	*0.00*
Gd	0.00	0.00	0.00	0.00	*0.00*
Dy	0.00	0.00	0.00	0.00	*0.00*
Ca	4.99	5.01	4.96	4.97	*4.98*
Fe	0.05	0.09	0.04	0.08	*0.07*
Mn	0.00	0.01	0.00	0.01	*0.00*
Sr	0.00	0.00	0.00	0.00	*0.00*
Na	0.00	0.00	0.00	0.00	*0.00*
F	0.93	1.03	0.99	1.03	*1.00*
Cl	0.04	0.02	0.01	0.01	*0.02*
OH	0.03	0.00	0.02	0.02	*1.01*
Total	1.00	1.05	1.02	1.06	*1.03*
XF/XOH	33.09		59.58	61.95	*51.54*
XCl/XOH	1.56		0.78	0.67	*1.01*

Page 3										
Metagabbro sample	3									*3*
No. on Map	105									*105*
No of grains analysed	1	2	3	4	5	6	7	8	9	10	*10*
Sample no	NIL25-4	NIL25-4	NIL25-4	NIL25-4	NIL25-4	NIL25-4	NIL25-4	NIL25-4	NIL25-4	NIL25-4	NIL25-4
											Mean
P_2O_5	39.84	41.27	41.94	40.47	40.57	41.16	40.76	40.97	40.62	41.01	*40.86*
SiO_2	0.12	0.27	0.01	0.15	0.15	0.02	0.07	0.05	0.07	0.02	*0.09*
Al_2O_3	0.00	0.02	0.07	0.02	0.05	0.00	0.01	0.00	0.00	0.07	*0.02*
SO_2	0.03	0.00	0.02	0.02	0.01	0.00	0.00	0.03	0.00	0.01	*0.01*
Y_2O_3	0.00	0.02	0.01	0.00	0.02	0.02	0.00	0.00	0.00	0.00	*0.01*
La_2O_3	0.10	0.00	0.05	0.01	0.00	0.03	0.00	0.03	0.03	0.01	*0.03*
Ce_2O_3	0.14	0.10	0.10	0.16	0.05	0.05	0.07	0.03	0.01	0.02	*0.07*
Pr_2O_3	0.00	0.00	0.00	0.02	0.00	0.03	0.00	0.00	0.00	0.00	*0.01*
Nd_2O_3	0.10	0.05	0.03	0.04	0.00	0.02	0.00	0.03	0.00	0.00	*0.03*
Sm_2O_3	0.00	0.00	0.02	0.00	0.00	0.03	0.00	0.00	0.00	0.00	*0.01*
Gd_2O_3	0.00	0.00	0.00	0.00	0.00	0.00	0.00	0.00	0.00	0.00	*0.00*
Dy_2O_3	0.00	0.00	0.00	0.00	0.00	0.00	0.03	0.00	0.00	0.00	*0.00*
CaO	53.70	54.71	55.05	53.91	54.34	54.79	54.67	54.52	54.51	54.89	*54.51*
FeO	0.65	0.52	0.67	0.44	0.49	0.33	0.33	0.31	0.26	0.15	*0.41*
MnO	0.10	0.03	0.01	0.09	0.02	0.03	0.07	0.01	0.00	0.03	*0.04*
SrO	0.00	0.00	0.00	0.00	0.00	0.00	0.00	0.00	0.00	0.00	*0.00*
Na_2O	0.00	0.00	0.00	0.00	0.00	0.02	0.04	0.00	0.00	0.00	*0.01*
F	1.35	0.85	1.21	1.52	1.50	1.44	0.78	1.33	1.80	1.67	*1.35*
Cl	2.95	2.86	2.84	2.83	2.82	2.67	2.63	2.63	2.67	2.64	*2.75*
H_2O	0.33	0.63	0.48	0.29	0.31	0.39	0.71	0.45	0.28	0.31	*0.42*
(F+Cl+OH)	4.63	4.35	4.52	4.64	4.63	4.50	4.12	4.41	4.75	4.62	*4.52*
sum	99.41	101.33	102.49	99.99	100.33	101.03	100.16	100.39	100.24	100.83	*100.62*
O=(F+Cl)	1.23	1.00	1.15	1.28	1.27	1.21	0.92	1.15	1.36	1.30	*1.19*
total	98.18	100.33	101.35	98.71	99.06	99.82	99.24	99.23	98.88	99.53	*99.43*
(Y+REE)	0.34	0.17	0.21	0.04	0.07	0.17	0.10	0.09	0.04	0.03	*0.13*
P	2.95	2.97	2.99	2.97	2.96	2.98	2.97	2.98	2.96	2.97	*2.97*
Si	0.01	0.02	0.00	0.01	0.01	0.00	0.01	0.00	0.01	0.00	*0.01*
Al	0.00	0.00	0.01	0.00	0.01	0.00	0.00	0.00	0.00	0.01	*0.00*
S	0.00	0.00	0.00	0.00	0.00	0.00	0.00	0.00	0.00	0.00	*0.00*
Y	0.00	0.00	0.00	0.00	0.00	0.00	0.00	0.00	0.00	0.00	*0.00*
La	0.00	0.00	0.00	0.00	0.00	0.00	0.00	0.00	0.00	0.00	*0.00*
Ce	0.00	0.00	0.00	0.01	0.00	0.00	0.00	0.00	0.00	0.00	*0.00*
Pr	0.00	0.00	0.00	0.00	0.00	0.00	0.00	0.00	0.00	0.00	*0.00*
Nd	0.00	0.00	0.00	0.00	0.00	0.00	0.00	0.00	0.00	0.00	*0.00*
Sm	0.00	0.00	0.00	0.00	0.00	0.00	0.00	0.00	0.00	0.00	*0.00*
Gd	0.00	0.00	0.00	0.00	0.00	0.00	0.00	0.00	0.00	0.00	*0.00*
Dy	0.00	0.00	0.00	0.00	0.00	0.00	0.00	0.00	0.00	0.00	*0.00*
Ca	5.03	4.98	4.96	5.00	5.02	5.02	5.03	5.01	5.04	5.03	*5.01*
Fe	0.09	0.07	0.09	0.06	0.07	0.05	0.05	0.04	0.04	0.02	*0.06*
Mn	0.02	0.00	0.00	0.01	0.00	0.00	0.01	0.00	0.00	0.00	*0.01*
Sr	0.00	0.00	0.00	0.00	0.00	0.00	0.00	0.00	0.00	0.00	*0.00*
Na	0.00	0.00	0.00	0.00	0.00	0.00	0.01	0.00	0.00	0.00	*0.00*
F	0.37	0.23	0.32	0.42	0.41	0.39	0.21	0.36	0.49	0.45	*0.37*
Cl	0.44	0.41	0.41	0.42	0.41	0.39	0.38	0.38	0.39	0.38	*0.40*
OH	0.19	0.36	0.27	0.17	0.18	0.22	0.40	0.26	0.16	0.18	*0.24*
Total	1.00	1.00	0.99	1.00	1.00	1.00	1.00	1.00	1.04	1.01	*1.00*
XF/XOH	1.97	0.64	1.20	2.47	2.29	1.74	0.53	1.41	3.05	2.55	*1.78*
XCl/XOH	2.31	1.15	1.52	2.46	2.30	1.73	0.95	1.49	2.42	2.16	*1.85*

Page 4													
Metagabbro sample	4												*4*
No. on Map	105												*105*
No of grains analysed	1	2	3	4	5	6	7	8	9	10	11	12	13	*13*
Sample no	NIL25-4	NIL25-4	NIL25-4	NIL25-4	NIL25-4	NIL25-4	NIL25-4	NIL25-4	NIL25-4	NIL25-4	NIL25-4	NIL25-4	NIL25-4	NIL25-4
													Mean
P_2O_5	41.64	40.89	41.08	41.21	41.95	41.21	40.64	41.79	41.79	41.78	40.97	41.09	40.62	*41.28*
SiO_2	0.01	0.02	0.08	0.10	0.07	0.00	0.18	0.00	0.06	0.04	0.00	0.16	0.07	*0.06*
Al_2O_3	0.01	0.00	0.04	0.03	0.00	0.00	0.11	0.00	0.02	0.02	0.00	0.00	0.05	*0.02*
SO_2	0.00	0.01	0.03	0.04	0.02	0.01	0.03	0.00	0.05	0.00	0.01	0.05	0.00	*0.02*
Y_2O_3	0.00	0.00	0.00	0.02	0.00	0.01	0.01	0.02	0.00	0.04	0.01	0.05	0.01	*0.01*
La_2O_3	0.00	0.04	0.06	0.00	0.01	0.01	0.02	0.02	0.04	0.03	0.05	0.00	0.01	*0.02*
Ce_2O_3	0.03	0.05	0.09	0.09	0.02	0.03	0.05	0.02	0.06	0.05	0.01	0.03	0.06	*0.04*
Pr_2O_3	0.05	0.07	0.00	0.11	0.05	0.18	0.11	0.09	0.00	0.00	0.04	0.04	0.00	*0.06*
Nd_2O_3	0.01	0.02	0.03	0.05	0.03	0.00	0.02	0.06	0.04	0.03	0.00	0.00	0.00	*0.02*
Sm_2O_3	0.00	0.00	0.00	0.03	0.00	0.00	0.00	0.12	0.04	0.00	0.12	0.02	0.00	*0.02*
Gd_2O_3	0.00	0.00	0.00	0.00	0.00	0.00	0.00	0.00	0.00	0.00	0.00	0.00	0.00	*0.00*
Dy_2O_3	0.00	0.00	0.00	0.00	0.00	0.00	0.00	0.00	0.08	0.00	0.00	0.00	0.10	*0.01*
CaO	55.02	54.54	54.52	54.36	54.28	54.09	54.72	54.33	54.49	54.17	54.55	54.41	54.82	*54.48*
FeO	0.15	0.12	0.43	0.09	0.13	0.55	0.12	0.11	0.50	0.19	0.17	0.15	0.13	*0.22*
MnO	0.00	0.00	0.01	0.01	0.09	0.04	0.04	0.04	0.03	0.02	0.02	0.06	0.03	*0.03*
SrO	0.00	0.01	0.00	0.00	0.00	0.00	0.00	0.00	0.00	0.00	0.00	0.00	0.00	*0.00*
Na_2O	0.00	0.00	0.00	1.10	0.00	0.92	0.13	0.06	0.04	0.05	0.00	0.01	0.05	*0.18*
F	1.74	1.74	1.66	1.85	2.20	1.30	1.43	1.21	1.38	1.34	2.04	1.90	1.60	*1.65*
Cl	2.04	1.91	1.89	1.52	1.47	1.44	1.26	1.20	1.18	1.16	1.16	1.10	1.07	*1.41*
H_2O	0.40	0.43	0.49	0.50	0.35	0.78	0.75	0.88	0.82	0.83	0.48	0.57	0.71	*0.62*
(F+Cl+OH)	4.18	4.08	4.04	3.87	4.02	3.51	3.43	3.30	3.38	3.33	3.68	3.57	3.38	*3.67*
sum	101.10	99.87	100.42	101.09	100.68	100.56	99.61	99.95	100.61	99.76	99.64	99.62	99.31	*100.17*
O=(F+Cl)	1.19	1.16	1.13	1.12	1.26	0.87	0.88	0.78	0.85	0.83	1.12	1.05	0.91	*1.01*
total	99.90	98.71	99.29	99.97	99.42	99.69	98.72	99.17	99.76	98.93	98.52	98.57	98.40	*99.16*
(Y+REE)	0.09	0.19	0.19	0.30	0.12	0.23	0.21	0.33	0.25	0.16	0.23	0.14	0.17	*0.20*
P	2.99	2.98	2.98	2.96	3.01	2.97	2.95	3.01	2.99	3.01	2.98	2.98	2.96	*2.98*
Si	0.00	0.00	0.01	0.01	0.01	0.00	0.02	0.00	0.01	0.00	0.00	0.01	0.01	*0.01*
Al	0.00	0.00	0.00	0.00	0.00	0.00	0.01	0.00	0.00	0.00	0.00	0.00	0.00	*0.00*
S	0.00	0.00	0.00	0.00	0.00	0.00	0.00	0.00	0.01	0.00	0.00	0.01	0.00	*0.00*
Y	0.00	0.00	0.00	0.00	0.00	0.00	0.00	0.00	0.00	0.00	0.00	0.00	0.00	*0.00*
La	0.00	0.00	0.00	0.00	0.00	0.00	0.00	0.00	0.00	0.00	0.00	0.00	0.00	*0.00*
Ce	0.00	0.00	0.00	0.00	0.00	0.00	0.00	0.00	0.00	0.00	0.00	0.00	0.00	*0.00*
Pr	0.00	0.00	0.00	0.00	0.00	0.00	0.00	0.00	0.00	0.00	0.00	0.00	0.00	*0.00*
Nd	0.00	0.00	0.00	0.00	0.00	0.00	0.00	0.00	0.00	0.00	0.00	0.00	0.00	*0.00*
Sm	0.00	0.00	0.00	0.00	0.00	0.00	0.00	0.00	0.00	0.00	0.00	0.00	0.00	*0.00*
Gd	0.00	0.00	0.00	0.00	0.00	0.00	0.00	0.00	0.00	0.00	0.00	0.00	0.00	*0.00*
Dy	0.00	0.00	0.00	0.00	0.00	0.00	0.00	0.00	0.00	0.00	0.00	0.00	0.00	*0.00*
Ca	5.01	5.03	5.00	4.95	4.93	4.94	5.03	4.95	4.94	4.94	5.02	4.99	5.06	*4.98*
Fe	0.02	0.02	0.06	0.01	0.02	0.08	0.02	0.02	0.07	0.03	0.02	0.02	0.02	*0.03*
Mn	0.00	0.00	0.00	0.00	0.01	0.01	0.01	0.01	0.00	0.00	0.00	0.01	0.00	*0.00*
Sr	0.00	0.00	0.00	0.00	0.00	0.00	0.00	0.00	0.00	0.00	0.00	0.00	0.00	*0.00*
Na	0.00	0.00	0.00	0.18	0.00	0.15	0.02	0.01	0.01	0.01	0.00	0.00	0.01	*0.03*
F	0.47	0.47	0.45	0.50	0.59	0.35	0.39	0.33	0.37	0.36	0.56	0.51	0.43	*0.44*
Cl	0.29	0.28	0.27	0.22	0.21	0.21	0.18	0.17	0.17	0.17	0.17	0.16	0.16	*0.20*
OH	0.23	0.25	0.28	0.28	0.20	0.44	0.43	0.50	0.46	0.47	0.28	0.33	0.41	*0.35*
Total	0.99	1.00	1.00	1.00	1.00	1.00	1.00	1.00	1.00	1.00	1.00	1.00	1.00	*1.00*
XF/XOH	2.06	1.91	1.62	1.76	2.97	0.79	0.90	0.65	0.80	0.77	2.01	1.58	1.06	*1.45*
XCl/XOH	1.30	1.12	0.99	0.77	1.06	0.47	0.42	0.35	0.37	0.35	0.61	0.49	0.38	*0.67*

Page 5											
Metagabbro sample	5										*5*
No. on Map	109										*109*
No of grains analysed	1	2	3	4	5	6	7	8	9	10	11	*11*
Sample no	NIL23-10	NIL23-10	NIL23-10	NIL23-10	NIL23-10	NIL23-10	NIL23-10	NIL23-10	NIL23-10	NIL23-10	NIL23-10	NIL23-10
												Mean
P_2O_5	41.11	42.36	40.65	40.96	42.45	41.13	40.92	41.48	42.21	39.96	41.07	*41.30*
SiO_2	0.13	0.24	0.19	0.13	0.09	0.14	0.00	0.05	0.05	0.19	0.17	*0.13*
Al_2O_3	0.03	0.01	0.10	0.03	0.02	0.00	0.00	0.00	0.08	0.00	0.09	*0.03*
SO_2	0.01	0.03	0.04	0.00	0.03	0.03	0.02	0.00	0.01	0.02	0.01	*0.02*
Y_2O_3	0.00	0.01	0.02	0.00	0.00	0.00	0.04	0.00	0.00	0.00	0.00	*0.01*
La_2O_3	0.00	0.00	0.07	0.00	0.00	0.03	0.00	0.01	0.03	0.07	0.03	*0.02*
Ce_2O_3	0.12	0.11	0.06	0.10	0.04	0.10	0.13	0.05	0.01	0.20	0.12	*0.09*
Pr_2O_3	0.00	0.00	0.00	0.06	0.02	0.00	0.03	0.00	0.04	0.14	0.00	*0.03*
Nd_2O_3	0.07	0.14	0.01	0.00	0.06	0.09	0.12	0.00	0.05	0.08	0.08	*0.06*
Sm_2O_3	0.00	0.00	0.00	0.00	0.00	0.00	0.00	0.00	0.00	0.00	0.00	*0.00*
Gd_2O_3	0.00	0.00	0.00	0.00	0.00	0.00	0.03	0.00	0.00	0.00	0.00	*0.00*
Dy_2O_3	0.00	0.00	0.00	0.00	0.00	0.00	0.06	0.00	0.00	0.00	0.00	*0.01*
CaO	54.81	55.14	54.68	54.96	54.59	55.08	55.07	54.87	55.25	54.27	54.35	*54.82*
FeO	0.17	0.33	0.41	0.18	0.17	0.31	0.42	0.13	0.18	0.20	0.17	*0.24*
MnO	0.13	0.00	0.03	0.03	0.04	0.01	0.04	0.00	0.04	0.02	0.01	*0.03*
SrO	0.09	0.03	0.00	0.00	0.01	0.00	0.00	0.04	0.01	0.00	0.00	*0.02*
Na_2O	0.00	0.00	0.00	0.00	0.00	0.00	0.03	0.00	0.00	0.00	0.00	*0.00*
F	3.17	3.13	3.13	3.13	2.96	3.14	2.56	3.21	3.12	3.17	3.03	*3.07*
Cl	0.35	0.30	0.30	0.29	0.28	0.27	0.22	0.21	0.19	0.14	0.11	*0.24*
H_2O	0.25	0.25	0.30	0.30	0.32	0.25	0.50	0.25	0.27	0.20	0.30	*0.29*
(F+Cl+OH)	3.77	3.68	3.73	3.72	3.56	3.66	3.29	3.67	3.58	3.51	3.44	*3.60*
sum	100.44	102.09	99.99	100.17	101.09	100.58	100.19	100.30	101.55	98.65	99.54	*100.42*
O=(F+Cl)	1.41	1.39	1.38	1.38	1.31	1.38	1.13	1.40	1.36	1.37	1.30	*1.35*
total	99.03	100.70	98.60	98.78	99.78	99.20	99.06	98.90	100.19	97.29	98.24	*99.07*
(Y+REE)	0.20	0.26	0.16	0.16	0.12	0.22	0.40	0.06	0.12	0.48	0.23	*0.22*
P	2.96	2.99	2.94	2.96	3.02	2.96	2.96	2.99	3.00	2.94	2.98	*2.97*
Si	0.01	0.02	0.02	0.01	0.01	0.01	0.00	0.00	0.00	0.02	0.01	*0.01*
Al	0.00	0.00	0.01	0.00	0.00	0.00	0.00	0.00	0.01	0.00	0.01	*0.00*
S	0.00	0.00	0.01	0.00	0.00	0.00	0.00	0.00	0.00	0.00	0.00	*0.00*
Y	0.00	0.00	0.00	0.00	0.00	0.00	0.00	0.00	0.00	0.00	0.00	*0.00*
La	0.00	0.00	0.00	0.00	0.00	0.00	0.00	0.00	0.00	0.00	0.00	*0.00*
Ce	0.00	0.00	0.00	0.00	0.00	0.00	0.00	0.00	0.00	0.01	0.00	*0.00*
Pr	0.00	0.00	0.00	0.00	0.00	0.00	0.00	0.00	0.00	0.00	0.00	*0.00*
Nd	0.00	0.00	0.00	0.00	0.00	0.00	0.00	0.00	0.00	0.00	0.00	*0.00*
Sm	0.00	0.00	0.00	0.00	0.00	0.00	0.00	0.00	0.00	0.00	0.00	*0.00*
Gd	0.00	0.00	0.00	0.00	0.00	0.00	0.00	0.00	0.00	0.00	0.00	*0.00*
Dy	0.00	0.00	0.00	0.00	0.00	0.00	0.00	0.00	0.00	0.00	0.00	*0.00*
Ca	5.00	4.93	5.01	5.02	4.91	5.02	5.04	5.00	4.96	5.06	4.99	*4.99*
Fe	0.02	0.05	0.06	0.03	0.02	0.04	0.06	0.02	0.03	0.03	0.02	*0.03*
Mn	0.02	0.00	0.00	0.00	0.01	0.00	0.01	0.00	0.01	0.00	0.00	*0.00*
Sr	0.00	0.00	0.00	0.00	0.00	0.00	0.00	0.00	0.00	0.00	0.00	*0.00*
Na	0.00	0.00	0.00	0.00	0.00	0.00	0.00	0.00	0.00	0.00	0.00	*0.00*
F	0.85	0.83	0.85	0.84	0.79	0.84	0.69	0.86	0.83	0.87	0.82	*0.83*
Cl	0.05	0.04	0.04	0.04	0.04	0.04	0.03	0.03	0.03	0.02	0.02	*0.03*
OH	0.14	0.14	0.17	0.17	0.18	0.14	0.28	0.14	0.15	0.12	0.17	*0.16*
Total	1.05	1.01	1.06	1.06	1.01	1.03	1.01	1.04	1.01	1.01	1.01	*1.02*
XF/XOH	6.01	5.94	4.95	4.95	4.39	5.95	2.43	6.09	5.48	7.51	4.79	*5.32*
XCl/XOH	0.36	0.31	0.25	0.25	0.22	0.28	0.11	0.21	0.18	0.18	0.09	*0.22*

Page 6							
Metagabbro sample	6						*6*
No. on Map	102						*102*
No of grains analysed	1	2	3	4	5	6	*6*
Sample no	NIL10-84	NIL10-84	NIL10-84	NIL10-84	NIL10-84	NIL10-84	NIL10-84
							Mean
P_2O_5	42.03	42.53	41.77	42.77	40.43	42.34	*41.98*
SiO_2	0.03	0.01	0.10	0.04	0.09	0.05	*0.05*
Al_2O_3	0.00	0.00	0.00	0.12	0.00	0.01	*0.02*
SO_2	0.00	0.00	0.00	0.01	0.01	0.02	*0.01*
Y_2O_3	0.00	0.00	0.00	0.00	0.00	0.00	*0.00*
La_2O_3	0.02	0.05	0.00	0.05	0.03	0.00	*0.03*
Ce_2O_3	0.05	0.06	0.01	0.07	0.02	0.03	*0.04*
Pr_2O_3	0.02	0.04	0.00	0.12	0.00	0.02	*0.03*
Nd_2O_3	0.05	0.07	0.04	0.07	0.00	0.00	*0.04*
Sm_2O_3	0.00	0.00	0.00	0.00	0.00	0.00	*0.00*
Gd_2O_3	0.00	0.00	0.00	0.00	0.00	0.00	*0.00*
Dy_2O_3	0.00	0.00	0.00	0.00	0.00	0.00	*0.00*
CaO	55.11	55.68	54.76	54.90	54.37	55.55	*55.06*
FeO	0.06	0.09	0.25	0.15	0.04	0.61	*0.20*
MnO	0.00	0.05	0.00	0.01	0.00	0.00	*0.01*
SrO	0.00	0.00	0.00	0.00	0.00	0.00	*0.00*
Na_2O	0.00	0.00	0.00	0.00	0.00	0.00	*0.00*
F	2.84	3.32	3.30	3.25	3.34	3.42	*3.25*
Cl	0.16	0.14	0.14	0.08	0.06	0.06	*0.10*
H_2O	0.40	0.25	0.25	0.32	0.20	0.35	*0.30*
(F+Cl+OH)	3.40	3.71	3.69	3.65	3.60	3.83	*3.64*
sum	100.78	102.30	100.62	101.97	98.59	102.45	*101.12*
O=(F+Cl)	1.23	1.43	1.42	1.39	1.42	1.45	*1.39*
total	99.54	100.87	99.20	100.58	97.17	101.00	*99.73*
(Y+REE)	0.14	0.23	0.05	0.31	0.05	0.05	*0.14*
P	3.00	3.00	2.99	3.01	2.96	2.98	*2.99*
Si	0.00	0.00	0.01	0.00	0.01	0.00	*0.00*
Al	0.00	0.00	0.00	0.01	0.00	0.00	*0.00*
S	0.00	0.00	0.00	0.00	0.00	0.00	*0.00*
Y	0.00	0.00	0.00	0.00	0.00	0.00	*0.00*
La	0.00	0.00	0.00	0.00	0.00	0.00	*0.00*
Ce	0.00	0.00	0.00	0.00	0.00	0.00	*0.00*
Pr	0.00	0.00	0.00	0.00	0.00	0.00	*0.00*
Nd	0.00	0.00	0.00	0.00	0.00	0.00	*0.00*
Sm	0.00	0.00	0.00	0.00	0.00	0.00	*0.00*
Gd	0.00	0.00	0.00	0.00	0.00	0.00	*0.00*
Dy	0.00	0.00	0.00	0.00	0.00	0.00	*0.00*
Ca	4.98	4.97	4.96	4.89	5.05	4.95	*4.97*
Fe	0.01	0.01	0.04	0.02	0.01	0.08	*0.03*
Mn	0.00	0.01	0.00	0.00	0.00	0.00	*0.00*
Sr	0.00	0.00	0.00	0.00	0.00	0.00	*0.00*
Na	0.00	0.00	0.00	0.00	0.00	0.00	*0.00*
F	0.76	0.87	0.88	0.86	0.91	0.90	*0.86*
Cl	0.02	0.02	0.02	0.01	0.01	0.01	*0.01*
OH	0.23	0.14	0.14	0.18	0.12	0.19	*0.17*
Total	1.01	1.03	1.04	1.04	1.04	1.10	*1.04*
XF/XOH	3.37	6.30	6.26	4.81	7.92	4.63	*5.55*
XCl/XOH	0.10	0.14	0.14	0.06	0.07	0.04	*0.09*

Page 7														
Metagabbro sample	7													*7*
No. on Map	104													*104*
No of grains analysed	1	2	3	4	5	6	7	8	9	10	11	12	13	*13*
Sample no	NIL30-11	NIL30-11	NIL30-11	NIL30-11	NIL30-11	NIL30-11	NIL30-11	NIL30-11	NIL30-11	NIL30-11	NIL30-11	NIL30-11	NIL30-11	NIL30-11
														Mean
P_2O_5	41.90	40.76	42.35	40.77	40.39	41.10	42.52	40.27	42.30	40.65	42.56	41.81	41.84	*41.48*
SiO_2	0.14	0.12	0.10	0.49	0.04	0.47	0.08	0.43	0.18	0.47	0.07	0.05	0.06	*0.21*
Al_2O_3	0.05	0.06	0.01	0.09	0.03	0.03	0.00	0.02	0.00	0.02	0.00	0.07	0.12	*0.04*
SO_2	0.22	0.16	0.04	0.13	0.03	0.16	0.00	0.09	0.07	0.04	0.06	0.03	0.04	*0.08*
Y_2O_3	0.00	0.00	0.00	0.00	0.00	0.00	0.00	0.01	0.00	0.00	0.00	0.00	0.00	*0.00*
La_2O_3	0.05	0.00	0.02	0.04	0.01	0.10	0.02	0.00	0.03	0.03	0.01	0.03	0.05	*0.03*
Ce_2O_3	0.13	0.14	0.15	0.15	0.08	0.19	0.03	0.11	0.11	0.09	0.04	0.07	0.02	*0.10*
Pr_2O_3	0.02	0.01	0.00	0.01	0.04	0.00	0.01	0.01	0.05	0.02	0.01	0.03	0.00	*0.02*
Nd_2O_3	0.02	0.07	0.10	0.07	0.02	0.02	0.03	0.03	0.00	0.08	0.03	0.08	0.00	*0.04*
Sm_2O_3	0.00	0.00	0.00	0.00	0.00	0.04	0.00	0.00	0.00	0.00	0.00	0.00	0.00	*0.00*
Gd_2O_3	0.00	0.00	0.00	0.00	0.00	0.00	0.00	0.02	0.00	0.05	0.00	0.00	0.00	*0.01*
Dy_2O_3	0.00	0.00	0.00	0.00	0.00	0.00	0.00	0.00	0.00	0.00	0.00	0.00	0.00	*0.00*
CaO	55.03	55.16	55.49	54.85	55.24	53.14	55.29	55.21	55.85	55.17	55.02	55.35	55.53	*55.10*
FeO	0.34	0.47	0.45	0.16	0.32	0.12	0.38	0.12	0.14	0.38	0.20	0.12	0.08	*0.25*
MnO	0.00	0.00	0.00	0.00	0.03	0.02	0.00	0.02	0.03	0.01	0.02	0.09	0.10	*0.02*
SrO	0.00	0.00	0.02	0.08	0.06	0.02	0.08	0.01	0.00	0.05	0.00	0.02	0.00	*0.03*
Na_2O	0.00	0.04	0.00	0.02	0.00	0.02	0.00	0.02	0.01	0.02	0.02	0.00	0.00	*0.01*
F	3.17	3.33	3.08	3.65	3.22	3.35	3.70	3.41	3.38	3.67	3.62	3.79	3.56	*3.46*
Cl	0.65	0.64	0.33	0.33	0.33	0.32	0.31	0.30	0.26	0.25	0.21	0.21	0.20	*0.33*
H_2O	0.15	0.10	0.40	0.00	0.15	0.10	0.00	0.08	0.15	0.00	0.10	0.00	0.10	*0.10*
(F+Cl+OH)	3.97	4.07	3.81	3.98	3.70	3.77	4.01	3.79	3.79	3.92	3.93	4.00	3.86	*3.89*
sum	101.86	101.05	102.55	100.83	99.97	99.19	102.45	100.17	102.55	100.98	101.99	101.75	101.71	*101.31*
O=(F+Cl)	1.48	1.55	1.37	1.61	1.43	1.48	1.63	1.51	1.48	1.60	1.57	1.64	1.54	*1.53*
total	100.38	99.50	101.18	99.22	98.55	97.71	100.82	98.66	101.07	99.38	100.41	100.11	100.16	*99.78*
(Y+REE)	0.22	0.22	0.27	0.27	0.15	0.35	0.09	0.18	0.19	0.27	0.09	0.22	0.07	*0.20*
P	2.98	2.94	2.98	2.94	2.94	2.99	3.00	2.92	2.98	2.93	3.01	2.98	2.97	*2.97*
Si	0.01	0.01	0.01	0.04	0.00	0.04	0.01	0.04	0.01	0.04	0.01	0.00	0.00	*0.02*
Al	0.01	0.01	0.00	0.01	0.00	0.00	0.00	0.00	0.00	0.00	0.00	0.01	0.01	*0.00*
S	0.03	0.02	0.01	0.02	0.00	0.02	0.00	0.01	0.01	0.01	0.01	0.00	0.00	*0.01*
Y	0.00	0.00	0.00	0.00	0.00	0.00	0.00	0.00	0.00	0.00	0.00	0.00	0.00	*0.00*
La	0.00	0.00	0.00	0.00	0.00	0.00	0.00	0.00	0.00	0.00	0.00	0.00	0.00	*0.00*
Ce	0.00	0.00	0.00	0.00	0.00	0.01	0.00	0.00	0.00	0.00	0.00	0.00	0.00	*0.00*
Pr	0.00	0.00	0.00	0.00	0.00	0.00	0.00	0.00	0.00	0.00	0.00	0.00	0.00	*0.00*
Nd	0.00	0.00	0.00	0.00	0.00	0.00	0.00	0.00	0.00	0.00	0.00	0.00	0.00	*0.00*
Sm	0.00	0.00	0.00	0.00	0.00	0.00	0.00	0.00	0.00	0.00	0.00	0.00	0.00	*0.00*
Gd	0.00	0.00	0.00	0.00	0.00	0.00	0.00	0.00	0.00	0.00	0.00	0.00	0.00	*0.00*
Dy	0.00	0.00	0.00	0.00	0.00	0.00	0.00	0.00	0.00	0.00	0.00	0.00	0.00	*0.00*
Ca	4.95	5.03	4.94	5.00	5.09	4.89	4.94	5.07	4.98	5.03	4.92	4.99	5.00	*4.99*
Fe	0.05	0.07	0.06	0.02	0.05	0.02	0.05	0.02	0.02	0.05	0.03	0.02	0.01	*0.04*
Mn	0.00	0.00	0.00	0.00	0.00	0.00	0.00	0.00	0.00	0.00	0.00	0.01	0.01	*0.00*
Sr	0.00	0.00	0.00	0.00	0.00	0.00	0.00	0.00	0.00	0.00	0.00	0.00	0.00	*0.00*
Na	0.00	0.01	0.00	0.00	0.00	0.00	0.00	0.00	0.00	0.00	0.00	0.00	0.00	*0.00*
F	0.84	0.90	0.81	0.98	0.88	0.91	0.98	0.93	0.89	0.99	0.96	1.01	0.95	*0.92*
Cl	0.09	0.09	0.05	0.05	0.05	0.05	0.04	0.04	0.04	0.04	0.03	0.03	0.03	*0.05*
OH	0.08	0.06	0.22	0.00	0.09	0.06	0.00	0.05	0.08	0.00	0.06	0.00	0.06	*0.06*
Total	1.02	1.04	1.08	1.03	1.01	1.01	1.02	1.01	1.01	1.02	1.04	1.04	1.03	*1.03*
XF/XOH	10.02	15.79	3.65		10.18	15.87		20.23	10.68		17.16		16.88	*13.38*
XCl/XOH	1.10	1.62	0.21		0.55	0.82		0.95	0.44		0.54		0.51	*0.75*

Extended Data Table 4: Apatite compositions (wt%) in two-pyroxene granulite samples.

Page 1																	
Two pyroxene granulite sample	1																TPG
No. on map	80																80
Sample No	NIL29-1																NIL29-1
Number of grains analysed		1	2	3	4	5	6	7	8	9	11	12	13	14	15	16	16
																	Mean
P_2O_5		41.26	42.03	41.83	41.65	41.92	41.92	41.66	39.80	41.46	41.62	42.31	41.54	42.23	42.33	42.67	41.75
SiO_2		0.28	0.30	0.25	0.27	0.28	0.26	0.25	0.00	0.26	0.26	0.25	0.29	0.24	0.30	0.31	0.25
Al_2O_3		0.00	0.00	0.00	0.00	0.00	0.00	0.00	0.00	0.00	0.00	0.00	0.00	0.00	0.00	0.00	0.00
SO_2		0.23	0.23	0.28	0.22	0.10	0.21	0.33	0.03	0.28	0.27	0.10	0.19	0.24	0.06	0.04	0.19
Y_2O_3		0.04	0.08	0.07	0.03	0.07	0.04	0.04	0.01	0.01	0.03	0.13	0.06	0.06	0.06	0.04	0.05
La_2O_3		0.00	0.05	0.02	0.01	0.03	0.04	0.01	0.03	0.00	0.04	0.00	0.00	0.00	0.00	0.00	0.01
Ce_2O_3		0.07	0.05	0.14	0.22	0.15	0.17	0.04	0.03	0.15	0.03	0.05	0.05	0.06	0.10	0.06	0.09
Pr_2O_3		0.00	0.00	0.05	0.01	0.00	0.03	0.01	0.00	0.04	0.04	0.04	0.04	0.00	0.04	0.00	0.02
Nd_2O_3		0.09	0.06	0.05	0.13	0.11	0.11	0.09	0.06	0.12	0.03	0.04	0.09	0.08	0.06	0.00	0.07
Sm_2O_3		0.00	0.10	0.06	0.01	0.04	0.00	0.05	0.00	0.00	0.07	0.12	0.00	0.06	0.00	0.02	0.04
Gd_2O_3		0.07	0.05	0.01	0.00	0.00	0.00	0.00	0.00	0.00	0.00	0.06	0.06	0.00	0.00	0.05	0.02
Dy_2O_3		0.00	0.02	0.00	0.07	0.01	0.03	0.04	0.01	0.04	0.05	0.02	0.02	0.00	0.04	0.03	0.03
CaO		54.61	55.00	54.98	55.06	55.14	55.09	54.95	53.86	54.49	54.96	55.23	54.60	55.06	55.15	55.15	54.89
FeO		0.18	0.13	0.07	0.08	0.02	0.08	0.09	0.15	0.12	0.18	0.09	0.09	0.14	0.07	0.05	0.10
MnO		0.03	0.02	0.02	0.01	0.01	0.04	0.01	0.02	0.02	0.02	0.01	0.00	0.02	0.01	0.01	0.02
SrO		0.10	0.02	0.00	0.02	0.07	0.02	0.05	0.00	0.00	0.03	0.01	0.00	0.04	0.04	0.04	0.03
Na_2O		0.00	0.00	0.01	0.00	0.00	0.00	0.01	0.00	0.00	0.00	0.00	0.00	0.00	0.00	0.00	0.00
F		3.68	3.68	3.48	3.59	3.78	3.54	3.75	3.66	3.56	3.69	3.50	3.68	3.68	3.47	3.77	3.63
Cl		0.52	0.16	0.16	0.15	0.14	0.14	0.14	0.13	0.12	0.11	0.10	0.10	0.10	0.10	0.10	0.15
H_2O		0.01	0.10	0.10	0.10	0.10	0.10	0.10	0.10	0.10	0.10	0.10	0.10	0.10	0.10	0.10	0.09
(F+Cl+OH)		0.00	0.00	0.00	0.00	0.00	0.00	0.00	0.00	0.00	0.00	0.00	0.00	0.00	0.00	0.00	0.00
sum		101.17	102.07	101.57	101.62	101.98	101.83	101.61	97.89	100.77	101.51	102.15	100.91	102.12	101.94	102.45	101.44
O=(F+Cl)		1.67	1.59	1.50	1.54	1.62	1.52	1.61	1.57	1.53	1.58	1.50	1.57	1.57	1.48	1.61	1.56
total		99.51	100.48	100.07	100.08	100.36	100.31	100.00	96.32	99.25	99.93	100.65	99.34	100.54	100.46	100.84	99.88
(Y+REE)		0.28	0.40	0.40	0.47	0.42	0.42	0.28	0.14	0.36	0.28	0.44	0.32	0.26	0.31	0.21	0.33
P		2.96	2.97	2.97	2.96	2.97	2.97	2.96	2.95	2.97	2.96	2.99	2.97	2.98	2.99	3.00	2.97
Si		0.02	0.03	0.02	0.02	0.02	0.02	0.02	0.00	0.02	0.02	0.02	0.02	0.02	0.03	0.03	0.02
Al		0.00	0.00	0.00	0.00	0.00	0.00	0.00	0.00	0.00	0.00	0.00	0.00	0.00	0.00	0.00	0.00
S		0.03	0.03	0.04	0.03	0.01	0.03	0.04	0.00	0.04	0.03	0.01	0.02	0.03	0.01	0.01	0.02
Y		0.00	0.00	0.00	0.00	0.00	0.00	0.00	0.00	0.00	0.00	0.00	0.00	0.00	0.00	0.00	0.00
La		0.00	0.00	0.00	0.00	0.00	0.00	0.00	0.00	0.00	0.00	0.00	0.00	0.00	0.00	0.00	0.00
Ce		0.00	0.00	0.00	0.01	0.00	0.01	0.00	0.00	0.00	0.00	0.00	0.00	0.00	0.00	0.00	0.00
Pr		0.00	0.00	0.00	0.00	0.00	0.00	0.00	0.00	0.00	0.00	0.00	0.00	0.00	0.00	0.00	0.00
Nd		0.00	0.00	0.00	0.00	0.00	0.00	0.00	0.00	0.00	0.00	0.00	0.00	0.00	0.00	0.00	0.00
Sm		0.00	0.00	0.00	0.00	0.00	0.00	0.00	0.00	0.00	0.00	0.00	0.00	0.00	0.00	0.00	0.00
Gd		0.00	0.00	0.00	0.00	0.00	0.00	0.00	0.00	0.00	0.00	0.00	0.00	0.00	0.00	0.00	0.00
Dy		0.00	0.00	0.00	0.00	0.00	0.00	0.00	0.00	0.00	0.00	0.00	0.00	0.00	0.00	0.00	0.00
Ca		4.95	4.92	4.94	4.96	4.94	4.95	4.94	5.05	4.94	4.95	4.94	4.94	4.92	4.94	4.90	4.95
Fe		0.03	0.02	0.01	0.01	0.00	0.01	0.01	0.02	0.02	0.03	0.01	0.01	0.02	0.01	0.01	0.01
Mn		0.00	0.00	0.00	0.00	0.00	0.01	0.00	0.00	0.00	0.00	0.00	0.00	0.00	0.00	0.00	0.00
Sr		0.00	0.00	0.00	0.00	0.00	0.00	0.00	0.00	0.00	0.00	0.00	0.00	0.00	0.00	0.00	0.00
Na		0.00	0.00	0.00	0.00	0.00	0.00	0.00	0.00	0.00	0.00	0.00	0.00	0.00	0.00	0.00	0.00
F		0.98	0.97	0.92	0.95	1.00	0.94	1.00	1.01	0.95	0.98	0.92	0.98	0.97	0.92	0.99	0.97
Cl		0.07	0.02	0.02	0.02	0.02	0.02	0.02	0.02	0.02	0.02	0.01	0.01	0.01	0.01	0.01	0.02
OH		0.01	0.06	0.06	0.06	0.06	0.06	0.06	0.06	0.06	0.06	0.06	0.06	0.06	0.06	0.06	0.05
Total		1.07	1.05	1.00	1.03	1.08	1.01	1.07	1.09	1.03	1.05	0.99	1.05	1.04	0.99	1.06	1.04
XF/XOH			17.46	16.49	17.01	17.90	16.79	17.78	17.33	16.88	17.48	16.61	17.46	17.43	16.45	17.87	17.21
XCl/XOH			0.41	0.39	0.38	0.36	0.36	0.36	0.34	0.30	0.27	0.26	0.26	0.26	0.25	0.24	0.32

Page 2												
Two pyroxene granulite sample	2					*2*	Two pyroxene granulite sample	3		*3*	Two pyroxene granulite sample	6
No. on map	73					*73*	No. on map	84		*84*	No. on map	98
Sample No	NIL24-10					*NIL24-10*	Sample No	NIL29-8		*NIL29-8*	Sample No	NIL2-6
Number of grains analysed	1	2	3	4	5	*5*	Number of grains analysed	1	2	*2*	Number of grains analysed	1
						Mean				*Mean*		1
P_2O_5	41.83	42.35	38.31	41.49	42.39	*41.27*	P_2O_5	42.48	42.08	*42.28*	P_2O_5	40.53
SiO_2	0.00	0.00	0.00	0.00	0.00	*0.00*	SiO_2	0.00	0.00	*0.00*	SiO_2	0.18
Al_2O_3	0.00	0.00	0.00	0.00	0.00	*0.00*	Al_2O_3	0.00	0.00	*0.00*	Al_2O_3	0.00
SO_2	0.08	0.04	0.05	0.07	0.04	*0.05*	SO_2	0.02	0.01	*0.01*	SO_2	0.02
Y_2O_3	0.00	0.00	0.00	0.00	0.00	*0.00*	Y_2O_3	0.00	0.00	*0.00*	Y_2O_3	0.03
La_2O_3	0.02	0.01	0.00	0.02	0.00	*0.01*	La_2O_3	0.05	0.00	*0.02*	La_2O_3	0.07
Ce_2O_3	0.00	0.01	0.08	0.09	0.01	*0.04*	Ce_2O_3	0.06	0.00	*0.03*	Ce_2O_3	0.05
Pr_2O_3	0.04	0.00	0.02	0.03	0.00	*0.02*	Pr_2O_3	0.07	0.07	*0.07*	Pr_2O_3	0.00
Nd_2O_3	0.00	0.05	0.06	0.04	0.02	*0.03*	Nd_2O_3	0.09	0.06	*0.07*	Nd_2O_3	0.04
Sm_2O_3	0.04	0.00	0.00	0.03	0.03	*0.02*	Sm_2O_3	0.03	0.07	*0.05*	Sm_2O_3	0.01
Gd_2O_3	0.03	0.00	0.00	0.04	0.00	*0.01*	Gd_2O_3	0.06	0.00	*0.03*	Gd_2O_3	0.00
Dy_2O_3	0.00	0.02	0.00	0.04	0.02	*0.02*	Dy_2O_3	0.03	0.05	*0.04*	Dy_2O_3	0.01
CaO	57.01	57.29	55.41	56.55	54.90	*56.23*	CaO	56.65	57.27	*56.96*	CaO	54.80
FeO	0.21	0.26	0.36	0.14	0.07	*0.21*	FeO	0.06	0.09	*0.07*	FeO	0.10
MnO	0.00	0.00	0.00	0.00	0.00	*0.00*	MnO	0.00	0.00	*0.00*	MnO	0.03
SrO	0.00	0.00	0.01	0.00	0.00	*0.00*	SrO	0.00	0.00	*0.00*	SrO	0.11
Na_2O	0.00	0.00	0.01	0.00	0.00	*0.00*	Na_2O	0.00	0.00	*0.00*	Na_2O	0.01
F	3.22	3.74	3.77	3.78	3.78	*3.66*	F	3.61	3.74	*3.67*	F	3.52
Cl	0.18	0.17	0.16	0.15	0.15	*0.16*	Cl	0.29	0.12	*0.21*	Cl	0.18
H_2O	0.19	0.09	0.09	0.09	0.09	*0.11*	H_2O	0.10	0.10	*0.10*	H_2O	0.09
(F+Cl+OH)	0.00	0.00	0.00	0.00	0.00	*0.00*	(F+Cl+OH)	0.00	0.00	*0.00*	(F+Cl+OH)	0.00
sum	102.86	104.04	98.32	102.54	101.49	*101.85*	sum	103.57	103.65	*103.61*	sum	99.75
O=(F+Cl)	1.40	1.61	1.62	1.63	1.62	*1.58*	O=(F+Cl)	1.58	1.60	*1.59*	O=(F+Cl)	1.52
total	101.46	102.43	96.69	100.92	99.86	*100.27*	total	101.99	102.04	*102.01*	total	98.23
(Y+REE)	0.14	0.10	0.16	0.27	0.08	*0.15*	(Y+REE)	0.37	0.24	*0.31*	(Y+REE)	0.20
P	2.95	2.96	2.86	2.94	3.01	*2.94*	P	2.97	2.95	*2.96*	P	2.95
Si	0.00	0.00	0.00	0.00	0.00	*0.00*	Si	0.00	0.00	*0.00*	Si	0.02
Al	0.00	0.00	0.00	0.00	0.00	*0.00*	Al	0.00	0.00	*0.00*	Al	0.00
S	0.01	0.00	0.01	0.01	0.00	*0.01*	S	0.00	0.00	*0.00*	S	0.00
Y	0.00	0.00	0.00	0.00	0.00	*0.00*	Y	0.00	0.00	*0.00*	Y	0.00
La	0.00	0.00	0.00	0.00	0.00	*0.00*	La	0.00	0.00	*0.00*	La	0.00
Ce	0.00	0.00	0.00	0.00	0.00	*0.00*	Ce	0.00	0.00	*0.00*	Ce	0.00
Pr	0.00	0.00	0.00	0.00	0.00	*0.00*	Pr	0.00	0.00	*0.00*	Pr	0.00
Nd	0.00	0.00	0.00	0.00	0.00	*0.00*	Nd	0.00	0.00	*0.00*	Nd	0.00
Sm	0.00	0.00	0.00	0.00	0.00	*0.00*	Sm	0.00	0.00	*0.00*	Sm	0.00
Gd	0.00	0.00	0.00	0.00	0.00	*0.00*	Gd	0.00	0.00	*0.00*	Gd	0.00
Dy	0.00	0.00	0.00	0.00	0.00	*0.00*	Dy	0.00	0.00	*0.00*	Dy	0.00
Ca	5.09	5.06	5.24	5.07	4.93	*5.08*	Ca	5.02	5.08	*5.05*	Ca	5.05
Fe	0.03	0.04	0.05	0.02	0.01	*0.03*	Fe	0.01	0.01	*0.01*	Fe	0.01
Mn	0.00	0.00	0.00	0.00	0.00	*0.00*	Mn	0.00	0.00	*0.00*	Mn	0.00
Sr	0.00	0.00	0.00	0.00	0.00	*0.00*	Sr	0.00	0.00	*0.00*	Sr	0.01
Na	0.00	0.00	0.00	0.00	0.00	*0.00*	Na	0.00	0.00	*0.00*	Na	0.00
F	0.85	0.97	1.05	1.00	1.00	*0.98*	F	0.94	0.98	*0.96*	F	0.96
Cl	0.03	0.02	0.02	0.02	0.02	*0.02*	Cl	0.04	0.02	*0.03*	Cl	0.03
OH	0.11	0.05	0.05	0.05	0.05	*0.06*	OH	0.06	0.06	*0.06*	OH	0.05
Total	0.98	1.05	1.13	1.07	1.07	*1.06*	Total	1.04	1.05	*1.05*	Total	1.03
XF/XOH	8.04	19.68	19.83	19.93	19.88	*17.48*	XF/XOH	17.10	17.74	*17.42*	XF/XOH	18.53
XCl/XOH	0.24	0.49	0.45	0.43	0.41	*0.40*	XCl/XOH	0.74	0.31	*0.52*	XCl/XOH	0.49

Page 3																
Two pyroxene granulite sample	4															*4*
No. on map	85															*85*
Sample No	NIL29-10															*NIL29-10*
Number of grains analysed	1	2	3	4	5	6	7	8	9	10	12	13	14	15	16	*16*
																Mean
P_2O_5	40.78	40.68	40.85	41.47	40.53	40.31	41.26	41.19	40.17	40.82	41.57	41.84	41.45	41.16	41.07	*41.01*
SiO_2	0.12	0.11	0.14	0.13	0.13	0.12	0.15	0.15	0.12	0.11	0.12	0.15	0.13	0.11	0.10	*0.13*
Al_2O_3	0.00	0.00	0.00	0.00	0.00	0.00	0.00	0.00	0.00	0.00	0.00	0.00	0.00	0.00	0.00	*0.00*
SO_2	0.00	0.00	0.00	0.01	0.00	0.04	0.01	0.00	0.01	0.03	0.01	0.02	0.00	0.00	0.00	*0.01*
Y_2O_3	0.25	0.53	0.22	0.14	0.26	0.16	0.09	0.17	0.32	0.25	0.13	0.20	0.16	0.17	0.22	*0.22*
La_2O_3	0.00	0.00	0.00	0.01	0.00	0.00	0.00	0.00	0.00	0.05	0.00	0.00	0.02	0.00	0.00	*0.01*
Ce_2O_3	0.00	0.01	0.05	0.02	0.09	0.05	0.02	0.00	0.07	0.15	0.03	0.00	0.02	0.01	0.02	*0.04*
Pr_2O_3	0.00	0.07	0.03	0.01	0.06	0.00	0.00	0.06	0.00	0.02	0.00	0.05	0.00	0.01	0.00	*0.02*
Nd_2O_3	0.04	0.02	0.05	0.02	0.10	0.08	0.00	0.00	0.14	0.14	0.04	0.00	0.00	0.04	0.10	*0.05*
Sm_2O_3	0.00	0.01	0.00	0.04	0.01	0.00	0.00	0.03	0.06	0.10	0.05	0.03	0.00	0.00	0.08	*0.03*
Gd_2O_3	0.00	0.00	0.00	0.11	0.08	0.06	0.00	0.00	0.10	0.00	0.09	0.00	0.00	0.05	0.00	*0.03*
Dy_2O_3	0.01	0.07	0.04	0.03	0.02	0.07	0.04	0.00	0.02	0.01	0.03	0.03	0.00	0.05	0.04	*0.03*
CaO	54.23	54.29	54.25	54.76	54.29	54.19	55.00	55.08	53.31	54.58	55.21	57.42	55.05	54.80	54.77	*54.75*
FeO	0.31	0.39	0.20	0.14	0.08	0.23	0.09	0.14	0.38	0.05	0.03	0.06	0.05	0.04	0.07	*0.15*
MnO	0.04	0.05	0.03	0.04	0.02	0.03	0.02	0.04	0.02	0.01	0.01	0.01	0.02	0.03	0.03	*0.03*
SrO	0.00	0.00	0.00	0.02	0.00	0.00	0.03	0.00	0.00	0.00	0.02	0.00	0.00	0.00	0.00	*0.00*
Na_2O	0.00	0.00	0.00	0.01	0.01	0.00	0.00	0.00	0.00	0.00	0.00	0.00	0.00	0.00	0.01	*0.00*
F	3.65	3.56	3.63	3.74	3.69	3.66	3.77	3.78	3.48	3.49	3.71	3.63	3.71	3.54	3.67	*3.65*
Cl	0.35	0.08	0.08	0.08	0.07	0.07	0.07	0.07	0.07	0.06	0.06	0.06	0.06	0.06	0.06	*0.09*
H_2O	0.10	0.10	0.10	0.10	0.10	0.10	0.10	0.10	0.10	0.10	0.10	0.10	0.10	0.10	0.10	*0.10*
(F+Cl+OH)	0.00	0.00	0.00	0.00	0.00	0.00	0.00	0.00	0.00	0.00	0.00	0.00	0.00	0.00	0.00	*0.00*
sum	99.89	99.96	99.69	100.87	99.54	99.17	100.63	100.79	98.35	99.96	101.22	103.59	100.77	100.17	100.32	*100.33*
O=(F+Cl)	1.62	1.52	1.55	1.59	1.57	1.56	1.60	1.61	1.48	1.48	1.58	1.54	1.58	1.50	1.56	*1.56*
total	98.28	98.45	98.14	99.28	97.97	97.62	99.03	99.18	96.87	98.48	99.64	102.05	99.19	98.67	98.77	*98.77*
(Y+REE)	0.31	0.71	0.39	0.39	0.62	0.43	0.14	0.25	0.70	0.71	0.37	0.31	0.20	0.33	0.46	*0.42*
P	2.96	2.96	2.97	2.97	2.96	2.95	2.97	2.96	2.97	2.96	2.97	2.94	2.97	2.97	2.97	*2.96*
Si	0.01	0.01	0.01	0.01	0.01	0.01	0.01	0.01	0.01	0.01	0.01	0.01	0.01	0.01	0.01	*0.01*
Al	0.00	0.00	0.00	0.00	0.00	0.00	0.00	0.00	0.00	0.00	0.00	0.00	0.00	0.00	0.00	*0.00*
S	0.00	0.00	0.00	0.00	0.00	0.00	0.00	0.00	0.00	0.00	0.00	0.00	0.00	0.00	0.00	*0.00*
Y	0.01	0.01	0.01	0.00	0.01	0.00	0.00	0.00	0.01	0.01	0.00	0.00	0.00	0.00	0.00	*0.00*
La	0.00	0.00	0.00	0.00	0.00	0.00	0.00	0.00	0.00	0.00	0.00	0.00	0.00	0.00	0.00	*0.00*
Ce	0.00	0.00	0.00	0.00	0.00	0.00	0.00	0.00	0.00	0.00	0.00	0.00	0.00	0.00	0.00	*0.00*
Pr	0.00	0.00	0.00	0.00	0.00	0.00	0.00	0.00	0.00	0.00	0.00	0.00	0.00	0.00	0.00	*0.00*
Nd	0.00	0.00	0.00	0.00	0.00	0.00	0.00	0.00	0.00	0.00	0.00	0.00	0.00	0.00	0.00	*0.00*
Sm	0.00	0.00	0.00	0.00	0.00	0.00	0.00	0.00	0.00	0.00	0.00	0.00	0.00	0.00	0.00	*0.00*
Gd	0.00	0.00	0.00	0.00	0.00	0.00	0.00	0.00	0.00	0.00	0.00	0.00	0.00	0.00	0.00	*0.00*
Dy	0.00	0.00	0.00	0.00	0.00	0.00	0.00	0.00	0.00	0.00	0.00	0.00	0.00	0.00	0.00	*0.00*
Ca	4.98	4.99	4.99	4.97	5.01	5.02	5.00	5.01	4.98	5.02	5.00	5.10	5.00	5.01	5.01	*5.01*
Fe	0.04	0.06	0.03	0.02	0.01	0.03	0.01	0.02	0.05	0.01	0.00	0.01	0.01	0.01	0.01	*0.02*
Mn	0.01	0.01	0.00	0.01	0.00	0.00	0.00	0.01	0.00	0.00	0.00	0.00	0.00	0.00	0.00	*0.00*
Sr	0.00	0.00	0.00	0.00	0.00	0.00	0.00	0.00	0.00	0.00	0.00	0.00	0.00	0.00	0.00	*0.00*
Na	0.00	0.00	0.00	0.00	0.00	0.00	0.00	0.00	0.00	0.00	0.00	0.00	0.00	0.00	0.00	*0.00*
F	0.99	0.97	0.99	1.00	1.01	1.00	1.01	1.01	0.96	0.95	0.99	0.95	0.99	0.96	0.99	*0.98*
Cl	0.05	0.01	0.01	0.01	0.01	0.01	0.01	0.01	0.01	0.01	0.01	0.01	0.01	0.01	0.01	*0.01*
OH	0.06	0.06	0.06	0.06	0.06	0.06	0.06	0.06	0.06	0.06	0.06	0.06	0.06	0.06	0.06	*0.06*
Total	1.10	1.04	1.05	1.07	1.07	1.07	1.08	1.08	1.03	1.01	1.06	1.02	1.06	1.02	1.05	*1.05*
XF/XOH	17.30	16.87	17.21	17.73	17.51	17.37	17.85	17.92	16.48	16.53	17.58	17.21	17.59	16.77	17.38	*17.29*
XCl/XOH	0.90	0.21	0.21	0.19	0.19	0.18	0.18	0.17	0.17	0.16	0.16	0.16	0.15	0.15	0.15	*0.22*

Page 4																			
Two pyroxene granulite sample	5																		*5*
No. on map																			*69*
Sample No	246	246	246	246	246	246	246	246	246	246	246	246	246	246	246	246	246	246	246	*NIL24-6*
Number of grains analysed	1	2	3	4	5	6	7	8	9	10	12	13	14	15	16	17	18	19	20	*20*
																			Mean
P_2O_5	41.81	42.00	40.96	42.25	40.16	41.64	40.91	41.66	41.70	40.43	40.85	42.27	42.06	42.25	42.22	41.45	41.68	40.56	42.48	*41.54*
SiO_2	0.00	0.00	0.35	0.00	0.10	0.06	0.11	0.07	0.11	0.05	0.77	0.00	0.00	0.00	0.00	0.29	0.00	0.01	0.00	*0.10*
Al_2O_3	0.00	0.00	0.00	0.00	0.00	0.00	0.00	0.00	0.00	0.00	0.00	0.00	0.00	0.00	0.00	0.00	0.00	0.00	0.00	*0.00*
SO_2	0.02	0.01	0.10	0.16	0.04	0.03	0.03	0.00	0.01	0.04	0.02	0.05	0.01	0.05	0.00	0.03	0.00	0.03	0.08	*0.04*
Y_2O_3	0.00	0.00	0.10	0.00	0.06	0.05	0.08	0.05	0.04	0.02	0.06	0.00	0.00	0.00	0.00	0.13	0.00	0.00	0.00	*0.03*
La_2O_3	0.00	0.05	0.02	0.03	0.00	0.00	0.04	0.00	0.00	0.00	0.00	0.00	0.01	0.05	0.00	0.01	0.00	0.00	0.16	*0.02*
Ce_2O_3	0.04	0.19	0.06	0.12	0.01	0.03	0.03	0.01	0.03	0.02	0.00	0.06	0.02	0.15	0.02	0.10	0.05	0.04	0.42	*0.07*
Pr_2O_3	0.00	0.03	0.04	0.14	0.00	0.00	0.00	0.01	0.02	0.06	0.04	0.23	0.03	0.06	0.00	0.04	0.10	0.03	0.18	*0.05*
Nd_2O_3	0.03	0.17	0.10	0.14	0.06	0.01	0.01	0.00	0.08	0.00	0.04	0.07	0.08	0.26	0.04	0.13	0.11	0.10	0.17	*0.08*
Sm_2O_3	0.05	0.02	0.00	0.09	0.00	0.00	0.00	0.00	0.00	0.00	0.00	0.04	0.00	0.07	0.03	0.00	0.06	0.03	0.05	*0.02*
Gd_2O_3	0.00	0.06	0.00	0.02	0.00	0.00	0.00	0.00	0.00	0.00	0.00	0.03	0.06	0.01	0.00	0.00	0.09	0.01	0.00	*0.01*
Dy_2O_3	0.04	0.03	0.00	0.02	0.00	0.00	0.00	0.00	0.00	0.00	0.00	0.00	0.01	0.07	0.00	0.00	0.01	0.02	0.00	*0.01*
CaO	57.22	56.14	55.05	56.62	55.03	55.67	55.06	55.70	55.04	55.57	55.06	56.97	56.29	56.51	56.12	55.09	57.16	54.63	56.02	*55.84*
FeO	0.20	0.11	0.12	0.19	0.07	0.18	0.13	0.24	0.04	0.06	0.05	0.07	0.05	0.19	0.03	0.19	0.12	0.93	0.16	*0.16*
MnO	0.00	0.00	0.07	0.00	0.03	0.05	0.03	0.02	0.08	0.02	0.02	0.00	0.00	0.00	0.00	0.00	0.00	0.00	0.00	*0.02*
SrO	0.00	0.00	0.00	0.00	0.00	0.04	0.00	0.03	0.00	0.02	0.00	0.00	0.00	0.00	0.00	0.00	0.00	0.00	0.00	*0.00*
Na_2O	0.00	0.00	0.01	0.00	0.00	0.00	0.00	0.00	0.01	0.00	0.00	0.00	0.00	0.00	0.00	0.00	0.00	0.00	0.00	*0.00*
F	3.47	3.56	3.46	3.74	3.80	3.86	3.20	3.62	3.28	3.50	3.72	3.72	3.71	3.72	3.72	3.68	3.67	3.67	3.83	*3.63*
Cl	0.26	0.18	0.18	0.17	0.17	0.17	0.17	0.17	0.17	0.16	0.16	0.16	0.15	0.15	0.15	0.15	0.15	0.14	0.13	*0.17*
H_2O	0.10	0.10	0.10	0.10	0.05	0.05	0.20	0.10	0.20	0.10	0.10	0.09	0.10	0.09	0.09	0.10	0.08	0.10	0.10	*0.10*
(F+Cl+OH)	0.00	0.00	0.00	0.00	0.00	0.00	0.00	0.00	0.00	0.00	0.00	0.00	0.00	0.00	0.00	0.00	0.00	0.00	0.00	*0.00*
sum	103.23	102.65	100.73	103.77	99.57	101.84	100.00	101.67	100.81	100.05	100.89	103.75	102.58	103.61	102.42	101.38	103.27	100.29	103.77	*101.91*
O=(F+Cl)	1.52	1.54	1.50	1.61	1.64	1.66	1.39	1.56	1.42	1.51	1.60	1.60	1.60	1.60	1.60	1.58	1.58	1.58	1.64	*1.56*
total	101.71	101.11	99.23	102.16	97.94	100.18	98.62	100.11	99.39	98.54	99.29	102.15	100.98	102.02	100.82	99.80	101.69	98.71	102.13	*100.35*
(Y+REE)	0.16	0.55	0.32	0.55	0.14	0.09	0.16	0.07	0.17	0.11	0.13	0.43	0.20	0.67	0.09	0.40	0.42	0.22	0.97	*0.31*
P	2.95	2.97	2.95	2.96	2.93	2.96	2.96	2.97	2.99	2.94	2.93	2.96	2.97	2.96	2.98	2.96	2.94	2.94	2.97	*2.96*
Si	0.00	0.00	0.03	0.00	0.01	0.01	0.01	0.01	0.01	0.00	0.07	0.00	0.00	0.00	0.00	0.02	0.00	0.00	0.00	*0.01*
Al	0.00	0.00	0.00	0.00	0.00	0.00	0.00	0.00	0.00	0.00	0.00	0.00	0.00	0.00	0.00	0.00	0.00	0.00	0.00	*0.00*
S	0.00	0.00	0.01	0.02	0.00	0.00	0.00	0.00	0.00	0.00	0.00	0.01	0.00	0.01	0.00	0.00	0.00	0.00	0.01	*0.00*
Y	0.00	0.00	0.00	0.00	0.00	0.00	0.00	0.00	0.00	0.00	0.00	0.00	0.00	0.00	0.00	0.00	0.00	0.00	0.00	*0.00*
La	0.00	0.00	0.00	0.00	0.00	0.00	0.00	0.00	0.00	0.00	0.00	0.00	0.00	0.00	0.00	0.00	0.00	0.00	0.00	*0.00*
Ce	0.00	0.01	0.00	0.00	0.00	0.00	0.00	0.00	0.00	0.00	0.00	0.00	0.00	0.00	0.00	0.00	0.00	0.00	0.01	*0.00*
Pr	0.00	0.00	0.00	0.00	0.00	0.00	0.00	0.00	0.00	0.00	0.00	0.00	0.00	0.00	0.00	0.00	0.00	0.00	0.00	*0.00*
Nd	0.00	0.01	0.00	0.00	0.00	0.00	0.00	0.00	0.00	0.00	0.00	0.00	0.00	0.01	0.00	0.00	0.00	0.00	0.01	*0.00*
Sm	0.00	0.00	0.00	0.00	0.00	0.00	0.00	0.00	0.00	0.00	0.00	0.00	0.00	0.00	0.00	0.00	0.00	0.00	0.00	*0.00*
Gd	0.00	0.00	0.00	0.00	0.00	0.00	0.00	0.00	0.00	0.00	0.00	0.00	0.00	0.00	0.00	0.00	0.00	0.00	0.00	*0.00*
Dy	0.00	0.00	0.00	0.00	0.00	0.00	0.00	0.00	0.00	0.00	0.00	0.00	0.00	0.00	0.00	0.00	0.00	0.00	0.00	*0.00*
Ca	5.10	5.03	5.01	5.01	5.09	5.01	5.05	5.02	4.99	5.11	4.99	5.05	5.03	5.02	5.01	4.98	5.10	5.02	4.96	*5.03*
Fe	0.03	0.02	0.02	0.03	0.01	0.02	0.02	0.03	0.01	0.01	0.01	0.01	0.01	0.03	0.00	0.03	0.02	0.13	0.02	*0.02*
Mn	0.00	0.00	0.01	0.00	0.00	0.01	0.00	0.00	0.01	0.00	0.00	0.00	0.00	0.00	0.00	0.00	0.00	0.00	0.00	*0.00*
Sr	0.00	0.00	0.00	0.00	0.00	0.00	0.00	0.00	0.00	0.00	0.00	0.00	0.00	0.00	0.00	0.00	0.00	0.00	0.00	*0.00*
Na	0.00	0.00	0.00	0.00	0.00	0.00	0.00	0.00	0.00	0.00	0.00	0.00	0.00	0.00	0.00	0.00	0.00	0.00	0.00	*0.00*
F	0.91	0.94	0.93	0.98	1.04	1.03	0.87	0.96	0.88	0.95	1.00	0.97	0.98	0.97	0.98	0.98	0.97	0.99	1.00	*0.96*
Cl	0.04	0.03	0.03	0.02	0.03	0.02	0.02	0.02	0.02	0.02	0.02	0.02	0.02	0.02	0.02	0.02	0.02	0.02	0.02	*0.02*
OH	0.06	0.06	0.06	0.06	0.03	0.03	0.11	0.06	0.11	0.06	0.06	0.05	0.06	0.05	0.05	0.06	0.04	0.06	0.06	*0.06*
Total	1.00	1.02	1.01	1.06	1.09	1.08	1.00	1.04	1.01	1.03	1.08	1.04	1.06	1.04	1.05	1.06	1.03	1.07	1.07	*1.05*
XF/XOH	16.43	16.88	16.40	17.71	36.03	36.60	7.59	17.16	7.77	16.59	17.64	19.57	17.60	19.57	19.58	17.45	21.72	17.39	18.15	*18.83*
XCl/XOH	0.66	0.46	0.46	0.44	0.87	0.86	0.21	0.43	0.21	0.41	0.41	0.44	0.39	0.43	0.42	0.38	0.47	0.36	0.34	*0.46*

Extended Data Table 5: Apatite compositions (wt%) in charnockite samples.

Page 1									
Charnockite sample	1								*1*
No. on map	6								*6*
Sample No.	NIL25-5								NIL25-5
No of grains analysed	1	2	3	4	5	6	7	8	9	9
										Mean
P_2O_5	41.58	41.03	40.96	40.98	41.20	41.24	41.28	41.28	39.67	*41.02*
SiO_2	0.15	0.14	0.18	0.19	0.14	0.15	0.19	0.15	0.14	*0.16*
Al_2O_3	0.00	0.00	0.00	0.00	0.00	0.00	0.00	0.00	0.00	*0.00*
SO_2	0.00	0.02	0.02	0.02	0.01	0.00	0.01	0.01	0.00	*0.01*
Y_2O_3	0.05	0.05	0.04	0.01	0.00	0.06	0.01	0.00	0.00	*0.02*
La_2O_3	0.02	0.00	0.02	0.00	0.00	0.00	0.00	0.03	0.00	*0.01*
Ce_2O_3	0.02	0.03	0.14	0.09	0.05	0.08	0.03	0.06	0.04	*0.06*
Pr_2O_3	0.03	0.00	0.00	0.00	0.01	0.00	0.00	0.01	0.03	*0.01*
Nd_2O_3	0.10	0.01	0.15	0.05	0.11	0.07	0.08	0.09	0.04	*0.08*
Sm_2O_3	0.04	0.04	0.11	0.00	0.00	0.01	0.08	0.00	0.07	*0.04*
Gd_2O_3	0.00	0.00	0.05	0.00	0.00	0.03	0.06	0.11	0.00	*0.03*
Dy_2O_3	0.01	0.03	0.02	0.04	0.00	0.00	0.00	0.01	0.00	*0.01*
CaO	55.33	55.21	55.24	55.40	55.37	55.69	55.32	55.28	53.40	*55.14*
FeO	0.09	0.10	0.17	0.08	0.04	0.07	0.09	0.18	0.38	*0.13*
MnO	0.02	0.02	0.03	0.00	0.00	0.02	0.00	0.01	0.02	*0.01*
SrO	0.03	0.00	0.06	0.00	0.02	0.02	0.00	0.03	0.01	*0.02*
Na_2O	0.00	0.00	0.00	0.00	0.00	0.00	0.00	0.00	0.00	*0.00*
F	3.73	3.75	3.63	3.68	3.64	3.85	3.77	3.70	3.51	*3.70*
Cl	0.32	0.28	0.23	0.23	0.23	0.22	0.21	0.17	0.16	*0.23*
H_2O	0.10	0.10	0.10	0.10	0.10	0.10	0.10	0.10	0.10	*0.10*
(F+Cl+OH)	0.00	0.00	0.00	0.00	0.00	0.00	0.00	0.00	0.00	*0.00*
sum	101.61	100.81	101.13	100.86	100.90	101.63	101.23	101.23	97.57	*100.77*
O=(F+Cl)	1.64	1.64	1.58	1.60	1.58	1.67	1.63	1.60	1.51	*1.61*
total	99.97	99.17	99.55	99.26	99.32	99.96	99.60	99.63	96.05	*99.17*
(Y+REE)	0.27	0.16	0.51	0.18	0.17	0.26	0.26	0.31	0.19	*0.26*
P	2.97	2.95	2.95	2.95	2.96	2.95	2.96	2.96	2.95	*2.95*
Si	0.01	0.01	0.02	0.02	0.01	0.01	0.02	0.01	0.01	*0.01*
Al	0.00	0.00	0.00	0.00	0.00	0.00	0.00	0.00	0.00	*0.00*
S	0.00	0.00	0.00	0.00	0.00	0.00	0.00	0.00	0.00	*0.00*
Y	0.00	0.00	0.00	0.00	0.00	0.00	0.00	0.00	0.00	*0.00*
La	0.00	0.00	0.00	0.00	0.00	0.00	0.00	0.00	0.00	*0.00*
Ce	0.00	0.00	0.00	0.00	0.00	0.00	0.00	0.00	0.00	*0.00*
Pr	0.00	0.00	0.00	0.00	0.00	0.00	0.00	0.00	0.00	*0.00*
Nd	0.00	0.00	0.00	0.00	0.00	0.00	0.00	0.00	0.00	*0.00*
Sm	0.00	0.00	0.00	0.00	0.00	0.00	0.00	0.00	0.00	*0.00*
Gd	0.00	0.00	0.00	0.00	0.00	0.00	0.00	0.00	0.00	*0.00*
Dy	0.00	0.00	0.00	0.00	0.00	0.00	0.00	0.00	0.00	*0.00*
Ca	4.99	5.03	5.03	5.04	5.03	5.03	5.01	5.01	5.03	*5.02*
Fe	0.01	0.01	0.02	0.01	0.01	0.01	0.01	0.03	0.06	*0.02*
Mn	0.00	0.00	0.00	0.00	0.00	0.00	0.00	0.00	0.00	*0.00*
Sr	0.00	0.00	0.00	0.00	0.00	0.00	0.00	0.00	0.00	*0.00*
Na	0.00	0.00	0.00	0.00	0.00	0.00	0.00	0.00	0.00	*0.00*
F	0.99	1.01	0.98	0.99	0.98	1.03	1.01	0.99	0.98	*0.99*
Cl	0.04	0.04	0.03	0.03	0.03	0.03	0.03	0.02	0.02	*0.03*
OH	0.06	0.06	0.06	0.06	0.06	0.06	0.06	0.06	0.06	*0.06*
Total	1.10	1.11	1.07	1.08	1.07	1.12	1.09	1.07	1.06	*1.08*
XF/XOH	17.69	17.79	17.21	17.44	17.26	18.27	17.85	17.54	16.65	*17.52*
XCl/XOH	0.80	0.72	0.59	0.58	0.57	0.56	0.53	0.44	0.40	*0.58*

Page 2							
Charnockite sample	2						*2*
No. on map	8						*8*
Sample No.	NIL25-11						NIL25-11
No of grains analysed	1	2	3	4	5	6	7	7
							Mean
P_2O_5	38.94	38.01	38.98	38.49	38.73	38.57	38.42	*38.59*
SiO_2	0.10	0.12	0.12	0.12	0.11	0.10	0.11	*0.11*
Al_2O_3	0.00	0.00	0.00	0.00	0.00	0.00	0.00	*0.00*
SO_2	0.00	0.02	0.00	0.02	0.00	0.02	0.02	*0.01*
Y_2O_3	0.02	0.00	0.03	0.00	0.03	0.00	0.00	*0.01*
La_2O_3	0.00	0.01	0.00	0.00	0.00	0.00	0.00	*0.00*
Ce_2O_3	0.05	0.05	0.03	0.03	0.05	0.05	0.05	*0.04*
Pr_2O_3	0.00	0.00	0.02	0.00	0.03	0.00	0.00	*0.01*
Nd_2O_3	0.02	0.08	0.01	0.05	0.02	0.05	0.03	*0.04*
Sm_2O_3	0.00	0.00	0.08	0.01	0.04	0.00	0.01	*0.02*
Gd_2O_3	0.00	0.00	0.00	0.01	0.05	0.06	0.00	*0.02*
Dy_2O_3	0.00	0.00	0.00	0.00	0.01	0.00	0.00	*0.00*
CaO	55.55	55.46	55.02	55.24	55.52	55.73	55.30	*55.40*
FeO	0.06	0.38	0.24	0.05	0.06	0.07	0.04	*0.13*
MnO	0.01	0.03	0.01	0.01	0.01	0.03	0.00	*0.01*
SrO	0.00	0.00	0.02	0.04	0.02	0.00	0.03	*0.01*
Na_2O	0.00	0.01	0.01	0.00	0.01	0.01	0.00	*0.00*
F	2.98	3.25	3.27	3.24	2.98	3.11	3.10	*3.13*
Cl	0.32	0.31	0.25	0.19	0.18	0.12	0.03	*0.20*
H_2O	0.20	0.10	0.10	0.10	0.20	0.20	0.20	*0.16*
(F+Cl+OH)	0.00	0.00	0.00	0.00	0.00	0.00	0.00	*0.00*
sum	98.24	97.81	98.17	97.60	98.06	98.12	97.35	*97.91*
O=(F+Cl)	1.33	1.44	1.43	1.41	1.30	1.34	1.31	*1.36*
total	96.91	96.38	96.73	96.19	96.76	96.78	96.03	*96.54*
(Y+REE)	0.09	0.14	0.16	0.11	0.24	0.17	0.09	*0.14*
P	2.90	2.86	2.90	2.89	2.89	2.88	2.89	*2.89*
Si	0.01	0.01	0.01	0.01	0.01	0.01	0.01	*0.01*
Al	0.00	0.00	0.00	0.00	0.00	0.00	0.00	*0.00*
S	0.00	0.00	0.00	0.00	0.00	0.00	0.00	*0.00*
Y	0.00	0.00	0.00	0.00	0.00	0.00	0.00	*0.00*
La	0.00	0.00	0.00	0.00	0.00	0.00	0.00	*0.00*
Ce	0.00	0.00	0.00	0.00	0.00	0.00	0.00	*0.00*
Pr	0.00	0.00	0.00	0.00	0.00	0.00	0.00	*0.00*
Nd	0.00	0.00	0.00	0.00	0.00	0.00	0.00	*0.00*
Sm	0.00	0.00	0.00	0.00	0.00	0.00	0.00	*0.00*
Gd	0.00	0.00	0.00	0.00	0.00	0.00	0.00	*0.00*
Dy	0.00	0.00	0.00	0.00	0.00	0.00	0.00	*0.00*
Ca	5.23	5.28	5.19	5.25	5.24	5.26	5.26	*5.24*
Fe	0.01	0.06	0.04	0.01	0.01	0.01	0.01	*0.02*
Mn	0.00	0.00	0.00	0.00	0.00	0.00	0.00	*0.00*
Sr	0.00	0.00	0.00	0.00	0.00	0.00	0.00	*0.00*
Na	0.00	0.00	0.00	0.00	0.00	0.00	0.00	*0.00*
F	0.83	0.91	0.91	0.91	0.83	0.87	0.87	*0.88*
Cl	0.05	0.05	0.04	0.03	0.03	0.02	0.00	*0.03*
OH	0.12	0.06	0.06	0.06	0.12	0.12	0.12	*0.09*
Total	0.99	1.02	1.00	1.00	0.98	1.00	0.99	*1.00*
XF/XOH	7.07	15.38	15.48	15.36	7.06	7.38	7.36	*10.73*
XCl/XOH	0.41	0.77	0.63	0.48	0.23	0.15	0.04	*0.39*

Page 3													
Charnockite sample	3												3
No. on map	42												42
Sample No.	NIL2-18											NIL2-18	NIL2-18
No of grains analysed	1	2	3	4	5	6	7	8	9	10	11	12	13	13
													Mean
P_2O_5	41.12	40.11	40.10	40.77	41.97	40.41	42.16	42.42	41.19	42.28	42.36	40.88	40.09	41.22
SiO_2	0.15	0.17	0.17	0.17	0.00	0.14	0.00	0.00	0.20	0.00	0.00	0.13	0.16	0.10
Al_2O_3	0.00	0.00	0.00	0.00	0.00	0.00	0.00	0.00	0.00	0.00	0.00	0.00	0.00	0.00
SO_2	0.00	0.00	0.00	0.02	0.02	0.02	0.01	0.00	0.03	0.01	0.00	0.01	0.03	0.01
Y_2O_3	0.00	0.02	0.04	0.03	0.00	0.02	0.00	0.00	0.05	0.00	0.00	0.04	0.01	0.02
La_2O_3	0.00	0.05	0.00	0.00	0.02	0.00	0.00	0.04	0.00	0.01	0.00	0.05	0.00	0.01
Ce_2O_3	0.07	0.07	0.12	0.08	0.11	0.11	0.04	0.02	0.04	0.03	0.05	0.13	0.04	0.07
Pr_2O_3	0.00	0.00	0.04	0.00	0.06	0.03	0.00	0.00	0.03	0.00	0.01	0.03	0.04	0.02
Nd_2O_3	0.02	0.07	0.12	0.08	0.13	0.09	0.05	0.10	0.06	0.08	0.10	0.15	0.04	0.08
Sm_2O_3	0.06	0.02	0.06	0.00	0.04	0.03	0.01	0.06	0.00	0.04	0.07	0.03	0.00	0.03
Gd_2O_3	0.00	0.00	0.10	0.00	0.00	0.00	0.00	0.00	0.08	0.08	0.01	0.04	0.00	0.02
Dy_2O_3	0.00	0.03	0.05	0.06	0.00	0.00	0.00	0.04	0.03	0.01	0.00	0.00	0.00	0.02
CaO	53.72	52.95	51.83	53.05	56.58	53.23	56.67	56.58	53.85	56.57	56.97	54.16	53.32	54.57
FeO	0.19	0.35	0.93	0.26	0.26	0.06	0.18	0.04	0.02	0.05	0.05	0.05	0.15	0.20
MnO	0.00	0.01	0.02	0.00	0.00	0.00	0.00	0.00	0.00	0.00	0.00	0.02	0.00	0.00
SrO	0.00	0.00	0.01	0.00	0.00	0.00	0.00	0.00	0.02	0.00	0.00	0.01	0.00	0.00
Na_2O	0.00	0.00	0.01	0.01	0.00	0.01	0.00	0.00	0.01	0.00	0.00	0.00	0.03	0.00
F	3.68	3.41	3.58	3.72	3.32	3.45	3.80	3.42	3.74	3.68	3.76	3.67	3.61	3.60
Cl	1.27	1.24	0.76	0.52	0.47	0.45	0.45	0.24	0.22	0.21	0.20	0.16	0.14	0.49
H_2O	0.00	0.00	0.00	0.00	0.10	0.00	0.00	0.10	0.00	0.10	0.00	0.00	0.00	0.02
(F+Cl+OH)	0.00	0.00	0.00	0.00	0.00	0.00	0.00	0.00	0.00	0.00	0.00	0.00	0.00	0.00
sum	100.29	98.50	97.92	98.78	103.07	98.05	103.38	103.05	99.56	103.12	103.57	99.57	97.66	100.50
O=(F+Cl)	1.83	1.72	1.68	1.68	1.50	1.55	1.70	1.50	1.62	1.59	1.63	1.58	1.55	1.63
total	98.46	96.79	96.24	97.09	101.56	96.50	101.68	101.56	97.93	101.53	101.94	97.99	96.11	98.87
(Y+REE)	0.16	0.27	0.52	0.26	0.35	0.29	0.10	0.26	0.29	0.24	0.23	0.47	0.13	0.27
P	2.98	2.97	2.98	2.99	2.96	2.99	2.96	2.98	2.99	2.97	2.97	2.98	2.97	2.98
Si	0.01	0.02	0.01	0.01	0.00	0.01	0.00	0.00	0.02	0.00	0.00	0.01	0.01	0.01
Al	0.00	0.00	0.00	0.00	0.00	0.00	0.00	0.00	0.00	0.00	0.00	0.00	0.00	0.00
S	0.00	0.00	0.00	0.00	0.00	0.00	0.00	0.00	0.00	0.00	0.00	0.00	0.00	0.00
Y	0.00	0.00	0.00	0.00	0.00	0.00	0.00	0.00	0.00	0.00	0.00	0.00	0.00	0.00
La	0.00	0.00	0.00	0.00	0.00	0.00	0.00	0.00	0.00	0.00	0.00	0.00	0.00	0.00
Ce	0.00	0.00	0.00	0.00	0.00	0.00	0.00	0.00	0.00	0.00	0.00	0.00	0.00	0.00
Pr	0.00	0.00	0.00	0.00	0.00	0.00	0.00	0.00	0.00	0.00	0.00	0.00	0.00	0.00
Nd	0.00	0.00	0.00	0.00	0.00	0.00	0.00	0.00	0.00	0.00	0.00	0.00	0.00	0.00
Sm	0.00	0.00	0.00	0.00	0.00	0.00	0.00	0.00	0.00	0.00	0.00	0.00	0.00	0.00
Gd	0.00	0.00	0.00	0.00	0.00	0.00	0.00	0.00	0.00	0.00	0.00	0.00	0.00	0.00
Dy	0.00	0.00	0.00	0.00	0.00	0.00	0.00	0.00	0.00	0.00	0.00	0.00	0.00	0.00
Ca	4.92	4.95	4.87	4.92	5.06	4.98	5.04	5.03	4.95	5.03	5.06	4.99	5.00	4.99
Fe	0.03	0.05	0.14	0.04	0.04	0.01	0.02	0.00	0.00	0.01	0.01	0.01	0.02	0.03
Mn	0.00	0.00	0.00	0.00	0.00	0.00	0.00	0.00	0.00	0.00	0.00	0.00	0.00	0.00
Sr	0.00	0.00	0.00	0.00	0.00	0.00	0.00	0.00	0.00	0.00	0.00	0.00	0.00	0.00
Na	0.00	0.00	0.00	0.00	0.00	0.00	0.00	0.00	0.00	0.00	0.00	0.00	0.00	0.00
F	0.99	0.94	0.99	1.02	0.88	0.95	1.00	0.90	1.01	0.97	0.98	1.00	1.00	0.97
Cl	0.18	0.18	0.11	0.08	0.07	0.07	0.06	0.03	0.03	0.03	0.03	0.02	0.02	0.07
OH	0.00	0.00	0.00	0.00	0.06	0.00	0.00	0.06	0.00	0.06	0.00	0.00	0.00	0.01
Total	1.18	1.13	1.11	1.10	1.00	1.02	1.06	0.99	1.04	1.05	1.01	1.02	1.02	1.06
XF/XOH					15.74			16.22		17.43				16.46
XCl/XOH					1.19			0.61		0.52				0.78

Page 4													
Charnockite sample	3												3
No. on map	42												42
Sample No.	NIL2-18												NIL2-18
No of grains analysed	1	2	3	4	5	6	7	8	9	10	11	12	12
													Mean
P_2O_5	40.54	40.79	40.99	40.50	43.06	41.42	40.51	41.01	40.29	41.19	40.95	40.98	41.02
SiO_2	0.13	0.18	0.14	0.15	0.00	0.16	0.13	0.15	0.15	0.16	0.12	0.14	0.13
Al_2O_3	0.00	0.00	0.00	0.00	0.00	0.00	0.00	0.00	0.00	0.00	0.00	0.00	0.00
SO_2	0.01	0.00	0.03	0.00	0.01	0.00	0.02	0.00	0.02	0.00	0.01	0.00	0.01
Y_2O_3	0.01	0.00	0.00	0.04	0.00	0.02	0.00	0.00	0.00	0.00	0.01	0.00	0.01
La_2O_3	0.04	0.00	0.00	0.00	0.00	0.05	0.06	0.01	0.01	0.00	0.01	0.03	0.02
Ce_2O_3	0.09	0.02	0.02	0.08	0.00	0.09	0.23	0.09	0.10	0.04	0.08	0.13	0.08
Pr_2O_3	0.00	0.00	0.01	0.00	0.00	0.01	0.02	0.03	0.01	0.00	0.00	0.02	0.01
Nd_2O_3	0.07	0.03	0.04	0.08	0.11	0.09	0.26	0.05	0.13	0.09	0.17	0.13	0.10
Sm_2O_3	0.09	0.11	0.06	0.07	0.09	0.00	0.06	0.00	0.03	0.00	0.00	0.10	0.05
Gd_2O_3	0.00	0.02	0.01	0.05	0.04	0.03	0.00	0.02	0.00	0.02	0.00	0.00	0.02
Dy_2O_3	0.01	0.00	0.00	0.01	0.00	0.00	0.03	0.00	0.07	0.00	0.03	0.03	0.01
CaO	53.09	52.97	53.63	53.48	57.28	53.45	52.14	54.10	52.01	53.46	53.35	52.81	53.48
FeO	0.37	0.07	0.18	0.06	0.06	0.07	0.82	0.10	1.03	0.37	0.78	0.06	0.33
MnO	0.00	0.00	0.00	0.00	0.00	0.00	0.02	0.00	0.03	0.01	0.00	0.00	0.01
SrO	0.00	0.01	0.03	0.00	0.00	0.04	0.00	0.00	0.03	0.07	0.02	0.03	0.02
Na_2O	0.00	0.00	0.01	0.01	0.00	0.00	0.02	0.00	0.01	0.00	0.00	0.01	0.01
F	3.66	3.66	3.68	3.49	2.23	3.44	3.51	3.71	3.60	3.65	3.66	3.47	3.48
Cl	0.10	0.09	0.08	0.06	0.06	0.03	0.03	0.02	0.01	0.01	0.01	0.01	0.04
H_2O	0.10	0.10	0.10	0.10	0.10	0.15	0.10	0.10	0.10	0.10	0.10	0.10	0.10
(F+Cl+OH)	0.00	0.00	0.00	0.00	0.00	0.00	0.00	0.00	0.00	0.00	0.00	0.00	0.00
sum	98.30	98.05	99.00	98.17	103.03	99.05	97.96	99.39	97.62	99.18	99.29	98.04	98.92
O=(F+Cl)	1.56	1.56	1.57	1.48	0.95	1.45	1.48	1.56	1.52	1.54	1.54	1.46	1.47
total	96.74	96.49	97.43	96.68	102.08	97.60	96.47	97.82	96.10	97.64	97.74	96.58	97.45
(Y+REE)	0.31	0.19	0.13	0.32	0.24	0.29	0.67	0.20	0.35	0.15	0.29	0.45	0.30
P	2.98	3.00	2.99	2.98	3.03	3.01	2.99	2.98	2.99	3.00	2.98	3.01	2.99
Si	0.01	0.02	0.01	0.01	0.00	0.01	0.01	0.01	0.01	0.01	0.01	0.01	0.01
Al	0.00	0.00	0.00	0.00	0.00	0.00	0.00	0.00	0.00	0.00	0.00	0.00	0.00
S	0.00	0.00	0.00	0.00	0.00	0.00	0.00	0.00	0.00	0.00	0.00	0.00	0.00
Y	0.00	0.00	0.00	0.00	0.00	0.00	0.00	0.00	0.00	0.00	0.00	0.00	0.00
La	0.00	0.00	0.00	0.00	0.00	0.00	0.00	0.00	0.00	0.00	0.00	0.00	0.00
Ce	0.00	0.00	0.00	0.00	0.00	0.00	0.01	0.00	0.00	0.00	0.00	0.00	0.00
Pr	0.00	0.00	0.00	0.00	0.00	0.00	0.00	0.00	0.00	0.00	0.00	0.00	0.00
Nd	0.00	0.00	0.00	0.00	0.00	0.00	0.01	0.00	0.00	0.00	0.01	0.00	0.00
Sm	0.00	0.00	0.00	0.00	0.00	0.00	0.00	0.00	0.00	0.00	0.00	0.00	0.00
Gd	0.00	0.00	0.00	0.00	0.00	0.00	0.00	0.00	0.00	0.00	0.00	0.00	0.00
Dy	0.00	0.00	0.00	0.00	0.00	0.00	0.00	0.00	0.00	0.00	0.00	0.00	0.00
Ca	4.94	4.92	4.95	4.98	5.09	4.92	4.88	4.97	4.88	4.92	4.92	4.91	4.94
Fe	0.05	0.01	0.03	0.01	0.01	0.01	0.12	0.01	0.15	0.05	0.11	0.01	0.05
Mn	0.00	0.00	0.00	0.00	0.00	0.00	0.00	0.00	0.00	0.00	0.00	0.00	0.00
Sr	0.00	0.00	0.00	0.00	0.00	0.00	0.00	0.00	0.00	0.00	0.00	0.00	0.00
Na	0.00	0.00	0.00	0.00	0.00	0.00	0.00	0.00	0.00	0.00	0.00	0.00	0.00
F	1.00	1.00	1.00	0.96	0.58	0.93	0.97	1.01	1.00	0.99	1.00	0.95	0.95
Cl	0.01	0.01	0.01	0.01	0.01	0.00	0.00	0.00	0.00	0.00	0.00	0.00	0.01
OH	0.06	0.06	0.06	0.06	0.06	0.09	0.06	0.06	0.06	0.06	0.06	0.06	0.06
Total	1.08	1.07	1.07	1.03	0.65	1.02	1.03	1.07	1.06	1.05	1.06	1.01	1.02
XF/XOH	17.34	17.34	17.46	16.54	10.56	10.86	16.62	17.58	17.06	17.30	17.36	16.43	16.04
XCl/XOH	0.25	0.23	0.21	0.16	0.15	0.05	0.07	0.04	0.04	0.03	0.02	0.02	0.10

Page 5															
Charnockite sample	4														*4*
No. on map	27														*27*
Sample No.	NIL31-16														NIL31-16
No of grains analysed	1	2	3	4	5	6	7	8	9	10	11	12	13	14	15	15
															Mean
P_2O_5	40.85	40.99	41.67	40.51	41.64	40.93	41.44	41.00	41.08	41.74	41.66	41.68	41.61	41.75	41.35	*41.33*
SiO_2	0.15	0.15	0.14	0.13	0.16	0.13	0.15	0.12	0.17	0.13	0.13	0.12	0.13	0.14	0.17	*0.14*
Al_2O_3	0.00	0.00	0.00	0.00	0.00	0.00	0.00	0.00	0.00	0.00	0.00	0.00	0.00	0.00	0.00	*0.00*
SO_2	0.00	0.01	0.01	0.00	0.00	0.00	0.00	0.00	0.00	0.01	0.00	0.00	0.01	0.01	0.00	*0.00*
Y_2O_3	0.00	0.00	0.00	0.00	0.03	0.03	0.00	0.01	0.00	0.00	0.00	0.00	0.00	0.00	0.05	*0.01*
La_2O_3	0.11	0.02	0.00	0.02	0.00	0.00	0.00	0.01	0.01	0.00	0.01	0.01	0.00	0.01	0.04	*0.02*
Ce_2O_3	0.31	0.14	0.07	0.16	0.10	0.05	0.01	0.05	0.08	0.04	0.01	0.00	0.04	0.06	0.05	*0.08*
Pr_2O_3	0.05	0.03	0.04	0.05	0.03	0.00	0.01	0.00	0.04	0.01	0.00	0.04	0.03	0.00	0.00	*0.02*
Nd_2O_3	0.29	0.20	0.08	0.21	0.07	0.13	0.05	0.03	0.10	0.06	0.07	0.03	0.06	0.08	0.09	*0.10*
Sm_2O_3	0.02	0.09	0.05	0.14	0.04	0.03	0.00	0.03	0.04	0.00	0.09	0.04	0.00	0.00	0.04	*0.04*
Gd_2O_3	0.07	0.04	0.00	0.00	0.02	0.00	0.00	0.00	0.01	0.10	0.08	0.00	0.00	0.13	0.00	*0.03*
Dy_2O_3	0.04	0.02	0.00	0.04	0.00	0.01	0.00	0.04	0.00	0.01	0.01	0.00	0.00	0.00	0.01	*0.01*
CaO	53.86	54.50	54.70	53.24	54.90	53.97	55.12	54.10	54.16	54.87	54.98	54.75	54.85	54.77	54.73	*54.50*
FeO	0.23	0.30	0.21	0.17	0.49	0.08	0.06	0.23	0.10	0.24	0.11	0.18	0.21	0.08	0.08	*0.18*
MnO	0.00	0.00	0.02	0.00	0.00	0.01	0.02	0.02	0.00	0.00	0.00	0.01	0.00	0.00	0.00	*0.01*
SrO	0.05	0.01	0.03	0.00	0.05	0.00	0.03	0.00	0.01	0.06	0.00	0.00	0.03	0.04	0.00	*0.02*
Na_2O	0.00	0.00	0.00	0.00	0.00	0.00	0.00	0.00	0.00	0.00	0.00	0.00	0.00	0.00	0.00	*0.00*
F	3.57	3.65	3.77	3.60	3.68	3.62	3.73	3.62	3.48	3.72	3.79	3.73	3.79	3.74	3.53	*3.67*
Cl	0.05	0.04	0.04	0.03	0.03	0.03	0.02	0.02	0.02	0.02	0.02	0.02	0.02	0.02	0.02	*0.03*
H_2O	0.10	0.10	0.10	0.10	0.10	0.10	0.10	0.10	0.10	0.10	0.10	0.10	0.10	0.10	0.10	*0.10*
(F+Cl+OH)	0.00	0.00	0.00	0.00	0.00	0.00	0.00	0.00	0.00	0.00	0.00	0.00	0.00	0.00	0.00	*0.00*
sum	99.76	100.30	100.92	98.39	101.35	99.12	100.74	99.38	99.41	101.10	101.05	100.71	100.87	100.93	100.26	*100.28*
O=(F+Cl)	1.51	1.54	1.60	1.52	1.56	1.53	1.58	1.53	1.47	1.57	1.60	1.57	1.60	1.58	1.49	*1.55*
total	98.24	98.75	99.33	96.86	99.79	97.59	99.17	97.85	97.94	99.53	99.44	99.14	99.27	99.35	98.77	*98.73*
(Y+REE)	0.89	0.54	0.24	0.62	0.28	0.26	0.07	0.16	0.28	0.21	0.26	0.12	0.13	0.28	0.30	*0.31*
P	2.97	2.96	2.98	2.98	2.97	2.98	2.97	2.98	2.99	2.98	2.98	2.99	2.98	2.99	2.98	*2.98*
Si	0.01	0.01	0.01	0.01	0.01	0.01	0.01	0.01	0.01	0.01	0.01	0.01	0.01	0.01	0.01	*0.01*
Al	0.00	0.00	0.00	0.00	0.00	0.00	0.00	0.00	0.00	0.00	0.00	0.00	0.00	0.00	0.00	*0.00*
S	0.00	0.00	0.00	0.00	0.00	0.00	0.00	0.00	0.00	0.00	0.00	0.00	0.00	0.00	0.00	*0.00*
Y	0.00	0.00	0.00	0.00	0.00	0.00	0.00	0.00	0.00	0.00	0.00	0.00	0.00	0.00	0.00	*0.00*
La	0.00	0.00	0.00	0.00	0.00	0.00	0.00	0.00	0.00	0.00	0.00	0.00	0.00	0.00	0.00	*0.00*
Ce	0.01	0.00	0.00	0.01	0.00	0.00	0.00	0.00	0.00	0.00	0.00	0.00	0.00	0.00	0.00	*0.00*
Pr	0.00	0.00	0.00	0.00	0.00	0.00	0.00	0.00	0.00	0.00	0.00	0.00	0.00	0.00	0.00	*0.00*
Nd	0.01	0.01	0.00	0.01	0.00	0.00	0.00	0.00	0.00	0.00	0.00	0.00	0.00	0.00	0.00	*0.00*
Sm	0.00	0.00	0.00	0.00	0.00	0.00	0.00	0.00	0.00	0.00	0.00	0.00	0.00	0.00	0.00	*0.00*
Gd	0.00	0.00	0.00	0.00	0.00	0.00	0.00	0.00	0.00	0.00	0.00	0.00	0.00	0.00	0.00	*0.00*
Dy	0.00	0.00	0.00	0.00	0.00	0.00	0.00	0.00	0.00	0.00	0.00	0.00	0.00	0.00	0.00	*0.00*
Ca	4.96	4.99	4.96	4.96	4.96	4.98	5.00	4.98	4.98	4.96	4.98	4.96	4.97	4.96	4.99	*4.97*
Fe	0.03	0.04	0.03	0.02	0.07	0.01	0.01	0.03	0.01	0.03	0.02	0.03	0.03	0.01	0.01	*0.03*
Mn	0.00	0.00	0.00	0.00	0.00	0.00	0.00	0.00	0.00	0.00	0.00	0.00	0.00	0.00	0.00	*0.00*
Sr	0.00	0.00	0.00	0.00	0.00	0.00	0.00	0.00	0.00	0.00	0.00	0.00	0.00	0.00	0.00	*0.00*
Na	0.00	0.00	0.00	0.00	0.00	0.00	0.00	0.00	0.00	0.00	0.00	0.00	0.00	0.00	0.00	*0.00*
F	0.97	0.98	1.01	0.99	0.98	0.98	1.00	0.98	0.94	0.99	1.01	1.00	1.01	1.00	0.95	*0.99*
Cl	0.01	0.01	0.01	0.00	0.00	0.00	0.00	0.00	0.00	0.00	0.00	0.00	0.00	0.00	0.00	*0.00*
OH	0.06	0.06	0.06	0.06	0.06	0.06	0.06	0.06	0.06	0.06	0.06	0.06	0.06	0.06	0.06	*0.06*
Total	1.03	1.05	1.07	1.05	1.04	1.05	1.06	1.04	1.00	1.05	1.07	1.06	1.07	1.06	1.01	*1.05*
XF/XOH	16.92	17.28	17.88	17.05	17.47	17.15	17.68	17.18	16.48	17.65	17.98	17.66	17.95	17.71	16.73	*17.38*
XCl/XOH	0.12	0.09	0.09	0.08	0.07	0.06	0.06	0.06	0.06	0.05	0.05	0.05	0.05	0.04	0.04	*0.06*

Page 6															
Charnockite sample	5														*5*
No. on map	29														*29*
Sample No.	NIL31-18														NIL31-18
No of grains analysed	1	2	3	4	5	6	7	8	9	10	11	12	13	14	14
															Mean
P_2O_5	40.35	39.66	40.39	41.49	41.37	40.37	41.12	41.64	40.26	41.33	40.64	40.82	41.16	41.54	*40.86*
SiO_2	0.41	0.43	0.40	0.45	0.43	0.41	0.40	0.45	0.44	0.44	0.41	0.43	0.44	0.41	*0.42*
Al_2O_3	0.00	0.00	0.00	0.00	0.00	0.00	0.00	0.00	0.00	0.00	0.00	0.00	0.00	0.00	*0.00*
SO_2	0.00	0.00	0.00	0.00	0.00	0.00	0.00	0.00	0.01	0.00	0.00	0.01	0.00	0.00	*0.00*
Y_2O_3	0.04	0.00	0.02	0.00	0.00	0.00	0.00	0.01	0.00	0.00	0.00	0.02	0.02	0.00	*0.01*
La_2O_3	0.00	0.05	0.00	0.00	0.05	0.03	0.04	0.00	0.00	0.00	0.00	0.00	0.00	0.00	*0.01*
Ce_2O_3	0.20	0.18	0.02	0.01	0.07	0.03	0.04	0.04	0.15	0.06	0.01	0.08	0.04	0.06	*0.07*
Pr_2O_3	0.06	0.03	0.00	0.05	0.03	0.00	0.00	0.00	0.00	0.00	0.05	0.05	0.03	0.05	*0.03*
Nd_2O_3	0.13	0.16	0.08	0.02	0.06	0.07	0.10	0.08	0.14	0.09	0.05	0.03	0.11	0.05	*0.08*
Sm_2O_3	0.04	0.06	0.00	0.00	0.00	0.00	0.00	0.05	0.06	0.02	0.04	0.04	0.14	0.03	*0.03*
Gd_2O_3	0.07	0.00	0.00	0.12	0.03	0.00	0.07	0.08	0.00	0.00	0.02	0.01	0.12	0.03	*0.04*
Dy_2O_3	0.00	0.06	0.04	0.00	0.00	0.00	0.01	0.00	0.00	0.00	0.00	0.00	0.02	0.01	*0.01*
CaO	52.54	53.98	52.07	54.47	54.36	54.05	54.28	53.94	53.89	53.05	54.38	54.33	54.07	54.38	*53.84*
FeO	0.10	0.32	0.03	0.12	0.05	0.05	0.05	0.07	0.44	0.05	0.27	0.07	0.06	0.06	*0.12*
MnO	0.00	0.00	0.01	0.02	0.00	0.01	0.01	0.00	0.00	0.02	0.00	0.00	0.01	0.03	*0.01*
SrO	0.00	0.06	0.04	0.04	0.00	0.02	0.01	0.02	0.00	0.00	0.09	0.00	0.05	0.00	*0.02*
Na_2O	0.01	0.00	0.00	0.00	0.00	0.00	0.00	0.00	0.00	0.00	0.00	0.00	0.00	0.01	*0.00*
F	3.52	3.64	3.73	3.82	3.81	3.55	3.74	3.69	3.66	3.45	3.75	3.73	3.70	3.62	*3.67*
Cl	0.04	0.04	0.03	0.03	0.03	0.03	0.03	0.03	0.03	0.03	0.03	0.03	0.02	0.01	*0.03*
H_2O	0.10	0.10	0.10	0.10	0.10	0.10	0.10	0.10	0.10	0.10	0.10	0.10	0.10	0.10	*0.10*
(F+Cl+OH)	0.00	0.00	0.00	0.00	0.00	0.00	0.00	0.00	0.00	0.00	0.00	0.00	0.00	0.00	*0.00*
sum	97.60	98.77	96.96	100.75	100.38	98.73	99.98	100.21	99.17	98.65	99.83	99.73	100.11	100.39	*99.38*
O=(F+Cl)	1.49	1.54	1.58	1.61	1.61	1.50	1.58	1.56	1.55	1.46	1.59	1.58	1.56	1.53	*1.55*
total	96.11	97.23	95.38	99.14	98.77	97.23	98.40	98.65	97.62	97.19	98.25	98.15	98.55	98.86	*97.82*
(Y+REE)	0.54	0.55	0.15	0.20	0.24	0.14	0.25	0.27	0.35	0.17	0.17	0.22	0.50	0.23	*0.28*
P	2.98	2.92	2.99	2.97	2.97	2.96	2.97	2.99	2.94	3.01	2.95	2.96	2.97	2.98	*2.97*
Si	0.04	0.04	0.04	0.04	0.04	0.04	0.03	0.04	0.04	0.04	0.04	0.04	0.04	0.03	*0.04*
Al	0.00	0.00	0.00	0.00	0.00	0.00	0.00	0.00	0.00	0.00	0.00	0.00	0.00	0.00	*0.00*
S	0.00	0.00	0.00	0.00	0.00	0.00	0.00	0.00	0.00	0.00	0.00	0.00	0.00	0.00	*0.00*
Y	0.00	0.00	0.00	0.00	0.00	0.00	0.00	0.00	0.00	0.00	0.00	0.00	0.00	0.00	*0.00*
La	0.00	0.00	0.00	0.00	0.00	0.00	0.00	0.00	0.00	0.00	0.00	0.00	0.00	0.00	*0.00*
Ce	0.01	0.01	0.00	0.00	0.00	0.00	0.00	0.00	0.00	0.00	0.00	0.00	0.00	0.00	*0.00*
Pr	0.00	0.00	0.00	0.00	0.00	0.00	0.00	0.00	0.00	0.00	0.00	0.00	0.00	0.00	*0.00*
Nd	0.00	0.01	0.00	0.00	0.00	0.00	0.00	0.00	0.00	0.00	0.00	0.00	0.00	0.00	*0.00*
Sm	0.00	0.00	0.00	0.00	0.00	0.00	0.00	0.00	0.00	0.00	0.00	0.00	0.00	0.00	*0.00*
Gd	0.00	0.00	0.00	0.00	0.00	0.00	0.00	0.00	0.00	0.00	0.00	0.00	0.00	0.00	*0.00*
Dy	0.00	0.00	0.00	0.00	0.00	0.00	0.00	0.00	0.00	0.00	0.00	0.00	0.00	0.00	*0.00*
Ca	4.92	5.03	4.88	4.93	4.94	5.01	4.96	4.90	4.99	4.89	4.99	4.98	4.94	4.94	*4.95*
Fe	0.02	0.05	0.00	0.02	0.01	0.01	0.01	0.01	0.06	0.01	0.04	0.01	0.01	0.01	*0.02*
Mn	0.00	0.00	0.00	0.00	0.00	0.00	0.00	0.00	0.00	0.00	0.00	0.00	0.00	0.00	*0.00*
Sr	0.00	0.00	0.00	0.00	0.00	0.00	0.00	0.00	0.00	0.00	0.00	0.00	0.00	0.00	*0.00*
Na	0.00	0.00	0.00	0.00	0.00	0.00	0.00	0.00	0.00	0.00	0.00	0.00	0.00	0.00	*0.00*
F	0.97	1.00	1.03	1.02	1.02	0.97	1.01	0.99	1.00	0.94	1.02	1.01	1.00	0.97	*1.00*
Cl	0.01	0.01	0.00	0.00	0.00	0.00	0.00	0.00	0.00	0.00	0.00	0.00	0.00	0.00	*0.00*
OH	0.06	0.06	0.06	0.06	0.06	0.06	0.06	0.06	0.06	0.06	0.06	0.06	0.06	0.06	*0.06*
Total	1.04	1.06	1.10	1.08	1.08	1.03	1.07	1.05	1.06	1.00	1.08	1.07	1.06	1.03	*1.06*
XF/XOH	16.66	17.25	17.70	18.10	18.06	16.84	17.71	17.50	17.37	16.35	17.79	17.67	17.52	17.16	*17.41*
XCl/XOH	0.10	0.09	0.08	0.08	0.08	0.07	0.07	0.07	0.07	0.07	0.07	0.07	0.06	0.03	*0.07*

Page 7									
Charnockite sample	6								*6*
No. on map	18								*18*
Sample No.	NIL31-03								NIL31-03
No of grains analysed	1	2	3	4	5	6	7	8	9	9
									Mean
P_2O_5	41.60	41.10	41.10	40.53	40.34	41.57	41.25	41.09	41.70	*41.14*
SiO_2	0.12	0.12	0.14	0.15	0.14	0.11	0.15	0.14	0.12	*0.13*
Al_2O_3	0.00	0.00	0.00	0.00	0.00	0.00	0.00	0.00	0.00	*0.00*
SO_2	0.01	0.00	0.02	0.00	0.01	0.00	0.01	0.00	0.00	*0.01*
Y_2O_3	0.02	0.03	0.02	0.01	0.03	0.00	0.04	0.00	0.00	*0.02*
La_2O_3	0.01	0.02	0.00	0.03	0.03	0.00	0.00	0.00	0.03	*0.01*
Ce_2O_3	0.05	0.11	0.12	0.02	0.17	0.16	0.12	0.11	0.08	*0.10*
Pr_2O_3	0.02	0.05	0.00	0.02	0.00	0.01	0.06	0.05	0.01	*0.02*
Nd_2O_3	0.06	0.19	0.17	0.07	0.22	0.17	0.18	0.15	0.19	*0.15*
Sm_2O_3	0.12	0.09	0.07	0.00	0.09	0.00	0.01	0.07	0.02	*0.05*
Gd_2O_3	0.00	0.03	0.03	0.00	0.02	0.00	0.04	0.04	0.00	*0.02*
Dy_2O_3	0.00	0.03	0.03	0.00	0.02	0.05	0.00	0.01	0.00	*0.02*
CaO	55.53	54.45	54.89	54.29	54.30	55.24	55.15	54.92	55.38	*54.90*
FeO	0.55	0.63	0.87	0.70	1.23	0.66	0.77	0.81	0.97	*0.80*
MnO	0.04	0.01	0.01	0.01	0.02	0.02	0.02	0.01	0.01	*0.02*
SrO	0.01	0.01	0.00	0.00	0.05	0.00	0.06	0.01	0.00	*0.01*
Na_2O	0.00	0.01	0.01	0.01	0.00	0.00	0.00	0.00	0.00	*0.00*
F	3.46	3.64	3.72	3.67	3.51	3.71	3.71	3.62	3.52	*3.62*
Cl	0.07	0.04	0.04	0.03	0.03	0.03	0.03	0.02	0.02	*0.04*
H_2O	0.10	0.10	0.10	0.10	0.10	0.10	0.10	0.10	0.10	*0.10*
(F+Cl+OH)	0.00	0.00	0.00	0.00	0.00	0.00	0.00	0.00	0.00	*0.00*
sum	101.77	100.64	101.33	99.64	100.31	101.83	101.68	101.14	102.14	*101.16*
O=(F+Cl)	1.47	1.54	1.58	1.55	1.49	1.57	1.57	1.53	1.48	*1.53*
total	100.29	99.10	99.76	98.09	98.83	100.25	100.11	99.62	100.66	*99.63*
(Y+REE)	0.27	0.55	0.44	0.15	0.59	0.38	0.44	0.43	0.33	*0.40*
P	2.97	2.97	2.95	2.95	2.94	2.96	2.95	2.95	2.97	*2.96*
Si	0.01	0.01	0.01	0.01	0.01	0.01	0.01	0.01	0.01	*0.01*
Al	0.00	0.00	0.00	0.00	0.00	0.00	0.00	0.00	0.00	*0.00*
S	0.00	0.00	0.00	0.00	0.00	0.00	0.00	0.00	0.00	*0.00*
Y	0.00	0.00	0.00	0.00	0.00	0.00	0.00	0.00	0.00	*0.00*
La	0.00	0.00	0.00	0.00	0.00	0.00	0.00	0.00	0.00	*0.00*
Ce	0.00	0.00	0.00	0.00	0.01	0.00	0.00	0.00	0.00	*0.00*
Pr	0.00	0.00	0.00	0.00	0.00	0.00	0.00	0.00	0.00	*0.00*
Nd	0.00	0.01	0.01	0.00	0.01	0.00	0.01	0.00	0.01	*0.00*
Sm	0.00	0.00	0.00	0.00	0.00	0.00	0.00	0.00	0.00	*0.00*
Gd	0.00	0.00	0.00	0.00	0.00	0.00	0.00	0.00	0.00	*0.00*
Dy	0.00	0.00	0.00	0.00	0.00	0.00	0.00	0.00	0.00	*0.00*
Ca	5.01	4.97	4.99	5.00	5.00	4.98	4.99	5.00	4.98	*4.99*
Fe	0.08	0.09	0.12	0.10	0.18	0.09	0.11	0.11	0.14	*0.11*
Mn	0.01	0.00	0.00	0.00	0.00	0.00	0.00	0.00	0.00	*0.00*
Sr	0.00	0.00	0.00	0.00	0.00	0.00	0.00	0.00	0.00	*0.00*
Na	0.00	0.00	0.00	0.00	0.00	0.00	0.00	0.00	0.00	*0.00*
F	0.92	0.98	1.00	1.00	0.95	0.99	0.99	0.97	0.93	*0.97*
Cl	0.01	0.01	0.01	0.00	0.00	0.00	0.00	0.00	0.00	*0.01*
OH	0.06	0.06	0.06	0.06	0.06	0.06	0.06	0.06	0.06	*0.06*
Total	0.99	1.04	1.06	1.06	1.02	1.05	1.05	1.03	0.99	*1.03*
XF/XOH	16.40	17.24	17.64	17.37	16.64	17.61	17.58	17.14	16.67	*17.14*
XCl/XOH	0.18	0.11	0.10	0.09	0.08	0.08	0.07	0.06	0.04	*0.09*

Page 8										
Charnockite sample	7									*7*
No. on map	23									*23*
Sample No.	NIL31-12									NIL31-12
No of grains analysed	1	2	3	4	5	6	7	8	9	9
										Mean
P_2O_5	41.55	41.45	41.70	41.60	40.79	41.53	41.69	41.64	42.03	*41.55*
SiO_2	0.14	0.15	0.00	0.15	0.00	0.17	0.00	0.13	0.00	*0.08*
Al_2O_3	0.00	0.00	0.00	0.00	0.00	0.00	0.00	0.00	0.00	*0.00*
SO_2	0.04	0.00	0.00	0.01	0.02	0.01	0.01	0.02	0.00	*0.01*
Y_2O_3	0.00	0.02	0.00	0.04	0.00	0.02	0.00	0.00	0.00	*0.01*
La_2O_3	0.00	0.00	0.02	0.00	0.00	0.00	0.03	0.00	0.01	*0.01*
Ce_2O_3	0.09	0.07	0.09	0.13	0.08	0.04	0.06	0.07	0.14	*0.09*
Pr_2O_3	0.01	0.03	0.00	0.04	0.13	0.03	0.11	0.00	0.00	*0.04*
Nd_2O_3	0.14	0.08	0.12	0.17	0.18	0.16	0.18	0.10	0.26	*0.15*
Sm_2O_3	0.09	0.00	0.06	0.09	0.06	0.06	0.09	0.04	0.07	*0.06*
Gd_2O_3	0.11	0.00	0.02	0.06	0.00	0.05	0.11	0.00	0.05	*0.04*
Dy_2O_3	0.00	0.01	0.06	0.00	0.00	0.05	0.00	0.03	0.03	*0.02*
CaO	54.56	54.60	56.77	54.59	55.30	54.87	57.21	54.92	56.58	*55.49*
FeO	0.59	0.61	0.80	0.73	1.30	0.77	0.73	0.44	0.54	*0.72*
MnO	0.00	0.04	0.00	0.01	0.00	0.00	0.00	0.00	0.00	*0.01*
SrO	0.01	0.05	0.00	0.01	0.00	0.00	0.00	0.00	0.00	*0.01*
Na_2O	0.00	0.00	0.00	0.00	0.00	0.00	0.00	0.00	0.00	*0.00*
F	3.73	3.60	2.95	3.54	4.06	3.48	3.82	3.44	3.99	*3.62*
Cl	0.12	0.05	0.04	0.03	0.03	0.03	0.03	0.03	0.03	*0.04*
H_2O	0.10	0.10	0.10	0.10	0.10	0.10	0.10	0.10	0.10	*0.10*
(F+Cl+OH)	0.00	0.00	0.00	0.00	0.00	0.00	0.00	0.00	0.00	*0.00*
sum	101.28	100.85	102.73	101.28	102.06	101.35	104.16	100.96	103.81	*102.05*
O=(F+Cl)	1.60	1.52	1.25	1.50	1.72	1.47	1.61	1.45	1.69	*1.53*
total	99.68	99.33	101.48	99.78	100.34	99.88	102.55	99.51	102.12	*100.52*
(Y+REE)	0.43	0.21	0.37	0.50	0.45	0.40	0.57	0.24	0.55	*0.41*
P	2.97	2.98	2.96	2.98	2.92	2.97	2.93	2.98	2.95	*2.96*
Si	0.01	0.01	0.00	0.01	0.00	0.01	0.00	0.01	0.00	*0.01*
Al	0.00	0.00	0.00	0.00	0.00	0.00	0.00	0.00	0.00	*0.00*
S	0.00	0.00	0.00	0.00	0.00	0.00	0.00	0.00	0.00	*0.00*
Y	0.00	0.00	0.00	0.00	0.00	0.00	0.00	0.00	0.00	*0.00*
La	0.00	0.00	0.00	0.00	0.00	0.00	0.00	0.00	0.00	*0.00*
Ce	0.00	0.00	0.00	0.00	0.00	0.00	0.00	0.00	0.00	*0.00*
Pr	0.00	0.00	0.00	0.00	0.00	0.00	0.00	0.00	0.00	*0.00*
Nd	0.00	0.00	0.00	0.00	0.01	0.00	0.01	0.00	0.01	*0.00*
Sm	0.00	0.00	0.00	0.00	0.00	0.00	0.00	0.00	0.00	*0.00*
Gd	0.00	0.00	0.00	0.00	0.00	0.00	0.00	0.00	0.00	*0.00*
Dy	0.00	0.00	0.00	0.00	0.00	0.00	0.00	0.00	0.00	*0.00*
Ca	4.94	4.96	5.10	4.95	5.01	4.97	5.08	4.98	5.02	*5.00*
Fe	0.08	0.09	0.11	0.10	0.18	0.11	0.10	0.06	0.07	*0.10*
Mn	0.00	0.01	0.00	0.00	0.00	0.00	0.00	0.00	0.00	*0.00*
Sr	0.00	0.00	0.00	0.00	0.00	0.00	0.00	0.00	0.00	*0.00*
Na	0.00	0.00	0.00	0.00	0.00	0.00	0.00	0.00	0.00	*0.00*
F	1.00	0.96	0.78	0.95	1.09	0.93	1.00	0.92	1.04	*0.96*
Cl	0.02	0.01	0.01	0.00	0.00	0.00	0.00	0.00	0.00	*0.01*
OH	0.06	0.06	0.06	0.06	0.06	0.06	0.06	0.06	0.06	*0.06*
Total	1.07	1.03	0.84	1.01	1.15	0.99	1.06	0.98	1.10	*1.03*
XF/XOH	17.67	17.05	13.97	16.77	19.27	16.47	18.10	16.29	18.91	*17.17*
XCl/XOH	0.32	0.12	0.10	0.09	0.08	0.08	0.08	0.07	0.07	*0.11*

Page 9													
Charnockite sample	8												
No. on map	59												continued on next page
Sample No.	NIL3-20												NIL3-20
No of grains analysed	1	2	3	4	5	6	7	8	9	10	11	12	13
P_2O_5	41.85	41.64	41.54	42.24	42.19	41.78	41.72	40.10	41.57	41.33	41.35	41.79	41.05
SiO_2	0.13	0.11	0.14	0.11	0.12	0.11	0.11	0.11	0.12	0.08	0.10	0.16	0.10
Al_2O_3	0.00	0.00	0.00	0.00	0.00	0.00	0.00	0.00	0.00	0.00	0.00	0.00	0.00
SO_2	0.01	0.00	0.01	0.02	0.02	0.00	0.01	0.02	0.00	0.00	0.00	0.03	0.00
Y_2O_3	0.01	0.03	0.02	0.00	0.00	0.00	0.01	0.00	0.00	0.00	0.00	0.06	0.02
La_2O_3	0.02	0.00	0.00	0.04	0.01	0.00	0.00	0.00	0.00	0.00	0.00	0.00	0.03
Ce_2O_3	0.01	0.07	0.03	0.04	0.04	0.02	0.03	0.12	0.05	0.07	0.02	0.04	0.05
Pr_2O_3	0.00	0.04	0.05	0.00	0.04	0.00	0.00	0.03	0.00	0.03	0.08	0.00	0.02
Nd_2O_3	0.04	0.05	0.04	0.11	0.03	0.03	0.01	0.15	0.04	0.15	0.03	0.07	0.09
Sm_2O_3	0.00	0.11	0.09	0.04	0.00	0.00	0.00	0.04	0.02	0.03	0.03	0.02	0.00
Gd_2O_3	0.03	0.00	0.05	0.03	0.00	0.01	0.03	0.03	0.00	0.11	0.05	0.00	0.01
Dy_2O_3	0.02	0.00	0.00	0.00	0.01	0.05	0.00	0.00	0.00	0.02	0.05	0.03	0.00
CaO	54.27	54.31	54.49	54.72	54.43	54.51	54.45	53.32	54.25	54.14	54.18	54.30	53.60
FeO	0.03	0.12	0.04	0.04	0.05	0.04	0.04	0.04	0.04	0.05	0.05	0.05	0.31
MnO	0.02	0.01	0.00	0.01	0.04	0.01	0.02	0.01	0.01	0.01	0.00	0.01	0.00
SrO	0.00	0.01	0.01	0.00	0.00	0.04	0.00	0.01	0.00	0.00	0.00	0.04	0.00
Na_2O	0.00	0.01	0.00	0.01	0.01	0.01	0.01	0.00	0.00	0.00	0.00	0.00	0.00
F	3.51	3.71	3.68	3.75	3.77	3.57	3.74	3.54	3.76	3.49	3.71	3.67	3.43
Cl	0.02	0.02	0.02	0.02	0.02	0.02	0.02	0.02	0.02	0.02	0.02	0.02	0.02
H_2O	0.10	0.10	0.10	0.10	0.10	0.10	0.10	0.10	0.10	0.10	0.10	0.10	0.10
(F+Cl+OH)	0.00	0.00	0.00	0.00	0.00	0.00	0.00	0.00	0.00	0.00	0.00	0.00	0.00
sum	100.08	100.32	100.31	101.27	100.85	100.29	100.28	97.64	99.97	99.62	99.76	100.38	98.82
O=(F+Cl)	1.48	1.57	1.55	1.58	1.59	1.50	1.58	1.50	1.59	1.47	1.57	1.55	1.45
total	98.60	98.75	98.76	99.69	99.26	98.78	98.70	96.14	98.38	98.14	98.19	98.83	97.37
(Y+REE)	0.13	0.30	0.28	0.26	0.12	0.12	0.08	0.37	0.11	0.41	0.25	0.21	0.21
P	3.01	2.99	2.99	3.00	3.01	3.00	3.00	2.97	3.00	3.00	2.99	3.00	3.00
Si	0.01	0.01	0.01	0.01	0.01	0.01	0.01	0.01	0.01	0.01	0.01	0.01	0.01
Al	0.00	0.00	0.00	0.00	0.00	0.00	0.00	0.00	0.00	0.00	0.00	0.00	0.00
S	0.00	0.00	0.00	0.00	0.00	0.00	0.00	0.00	0.00	0.00	0.00	0.00	0.00
Y	0.00	0.00	0.00	0.00	0.00	0.00	0.00	0.00	0.00	0.00	0.00	0.00	0.00
La	0.00	0.00	0.00	0.00	0.00	0.00	0.00	0.00	0.00	0.00	0.00	0.00	0.00
Ce	0.00	0.00	0.00	0.00	0.00	0.00	0.00	0.00	0.00	0.00	0.00	0.00	0.00
Pr	0.00	0.00	0.00	0.00	0.00	0.00	0.00	0.00	0.00	0.00	0.00	0.00	0.00
Nd	0.00	0.00	0.00	0.00	0.00	0.00	0.00	0.00	0.00	0.00	0.00	0.00	0.00
Sm	0.00	0.00	0.00	0.00	0.00	0.00	0.00	0.00	0.00	0.00	0.00	0.00	0.00
Gd	0.00	0.00	0.00	0.00	0.00	0.00	0.00	0.00	0.00	0.00	0.00	0.00	0.00
Dy	0.00	0.00	0.00	0.00	0.00	0.00	0.00	0.00	0.00	0.00	0.00	0.00	0.00
Ca	4.94	4.94	4.96	4.93	4.91	4.96	4.95	5.00	4.95	4.97	4.96	4.93	4.95
Fe	0.00	0.02	0.01	0.01	0.01	0.00	0.01	0.01	0.00	0.01	0.01	0.01	0.04
Mn	0.00	0.00	0.00	0.00	0.01	0.00	0.00	0.00	0.00	0.00	0.00	0.00	0.00
Sr	0.00	0.00	0.00	0.00	0.00	0.00	0.00	0.00	0.00	0.00	0.00	0.00	0.00
Na	0.00	0.00	0.00	0.00	0.00	0.00	0.00	0.00	0.00	0.00	0.00	0.00	0.00
F	0.94	1.00	0.99	1.00	1.00	0.96	1.00	0.98	1.01	0.95	1.00	0.98	0.94
Cl	0.00	0.00	0.00	0.00	0.00	0.00	0.00	0.00	0.00	0.00	0.00	0.00	0.00
OH	0.06	0.06	0.06	0.06	0.06	0.06	0.06	0.06	0.06	0.06	0.06	0.06	0.06
Total	1.00	1.06	1.05	1.05	1.06	1.02	1.06	1.04	1.07	1.01	1.06	1.04	0.99
XF/XOH	16.65	17.57	17.43	17.77	17.85	16.90	17.71	16.80	17.83	16.55	17.59	17.40	16.25
XCl/XOH	0.06	0.05	0.05	0.04	0.04	0.04	0.04	0.04	0.04	0.04	0.04	0.04	0.04

Page 10																	
Charnockite sample	8 cont'd																8
No. on map	59																59
Sample No.	NIL3-20																NIL3-20
No of grains analysed	14	15	16	17	18	19	20	21	22	23	24	25	26	27	28	29	29
																	Mean
P_2O_5	41.32	41.02	41.75	42.10	41.13	41.65	41.44	42.21	41.92	41.64	41.53	40.64	40.99	41.89	41.80	41.27	41.52
SiO_2	0.12	0.09	0.12	0.13	0.23	0.13	0.03	0.16	0.15	0.05	0.18	0.05	0.05	0.05	0.43	0.05	0.12
Al_2O_3	0.00	0.00	0.00	0.00	0.00	0.00	0.00	0.00	0.00	0.00	0.00	0.00	0.00	0.00	0.00	0.00	0.00
SO_2	0.01	0.00	0.02	0.02	0.00	0.00	0.01	0.00	0.02	0.01	0.01	0.02	0.00	0.00	0.00	0.00	0.01
Y_2O_3	0.01	0.02	0.02	0.05	0.00	0.01	0.00	0.00	0.06	0.00	0.00	0.00	0.00	0.00	0.01	0.00	0.01
La_2O_3	0.00	0.00	0.00	0.02	0.08	0.00	0.01	0.00	0.05	0.02	0.00	0.07	0.00	0.00	0.00	0.00	0.01
Ce_2O_3	0.05	0.06	0.02	0.03	0.12	0.01	0.07	0.04	0.07	0.05	0.08	0.03	0.06	0.00	0.06	0.04	0.05
Pr_2O_3	0.01	0.10	0.02	0.00	0.05	0.00	0.00	0.02	0.00	0.00	0.01	0.13	0.00	0.00	0.00	0.03	0.02
Nd_2O_3	0.02	0.03	0.04	0.05	0.18	0.07	0.03	0.07	0.07	0.00	0.16	0.06	0.05	0.06	0.05	0.09	0.06
Sm_2O_3	0.00	0.00	0.02	0.04	0.00	0.03	0.00	0.00	0.00	0.00	0.00	0.00	0.00	0.00	0.00	0.00	0.02
Gd_2O_3	0.03	0.00	0.04	0.00	0.00	0.09	0.00	0.00	0.00	0.00	0.00	0.00	0.00	0.00	0.00	0.00	0.02
Dy_2O_3	0.00	0.00	0.02	0.01	0.00	0.01	0.00	0.00	0.02	0.00	0.00	0.00	0.00	0.00	0.00	0.00	0.01
CaO	54.20	55.43	54.32	54.43	54.80	54.61	55.29	55.10	55.14	55.09	54.98	54.91	55.01	55.27	54.60	54.95	54.59
FeO	0.04	0.03	0.03	0.04	0.07	0.04	0.11	0.01	0.05	0.09	0.17	0.05	0.08	0.08	0.07	0.07	0.07
MnO	0.01	0.00	0.02	0.02	0.08	0.01	0.00	0.01	0.00	0.03	0.00	0.08	0.01	0.00	0.00	0.00	0.01
SrO	0.03	0.00	0.00	0.00	0.00	0.00	0.00	0.01	0.01	0.00	0.00	0.00	0.00	0.00	0.00	0.00	0.01
Na_2O	0.00	0.00	0.00	0.01	0.00	0.00	0.00	0.00	0.00	0.00	0.00	0.00	0.00	0.00	0.00	0.00	0.00
F	3.50	3.57	3.63	3.56	3.69	3.71	3.37	3.73	3.71	3.43	3.73	3.67	3.15	3.63	3.88	3.69	3.62
Cl	0.02	0.01	0.01	0.01	0.01	0.01	0.01	0.01	0.01	0.01	0.01	0.00	0.00	0.00	0.00	0.00	0.01
H_2O	0.10	0.06	0.10	0.10	0.05	0.10	0.17	0.10	0.10	0.14	0.10	0.10	0.26	0.06	0.08	0.10	0.04
(F+Cl+OH)	0.00	0.00	0.00	0.00	0.00	0.00	0.00	0.00	0.00	0.00	0.00	0.00	0.00	0.00	0.00	0.00	0.00
sum	99.47	100.44	100.19	100.61	100.49	100.48	100.54	101.47	101.35	100.56	100.95	99.82	99.66	101.04	100.98	100.30	100.26
O=(F+Cl)	1.48	1.51	1.53	1.50	1.56	1.56	1.42	1.57	1.56	1.45	1.57	1.55	1.33	1.53	1.63	1.55	1.53
total	97.99	98.93	98.66	99.11	98.93	98.91	99.12	99.90	99.79	99.11	99.38	98.27	98.33	99.51	99.34	98.74	98.74
(Y+REE)	0.12	0.22	0.18	0.18	0.43	0.21	0.11	0.13	0.26	0.07	0.25	0.29	0.12	0.06	0.12	0.17	0.20
P	3.00	2.96	3.00	3.01	2.97	2.99	2.98	3.00	2.99	2.99	2.97	2.95	2.97	2.99	2.98	2.98	2.99
Si	0.01	0.01	0.01	0.01	0.02	0.01	0.00	0.01	0.01	0.00	0.02	0.00	0.00	0.00	0.04	0.00	0.01
Al	0.00	0.00	0.00	0.00	0.00	0.00	0.00	0.00	0.00	0.00	0.00	0.00	0.00	0.00	0.00	0.00	0.00
S	0.00	0.00	0.00	0.00	0.00	0.00	0.00	0.00	0.00	0.00	0.00	0.00	0.00	0.00	0.00	0.00	0.00
Y	0.00	0.00	0.00	0.00	0.00	0.00	0.00	0.00	0.00	0.00	0.00	0.00	0.00	0.00	0.00	0.00	0.00
La	0.00	0.00	0.00	0.00	0.00	0.00	0.00	0.00	0.00	0.00	0.00	0.00	0.00	0.00	0.00	0.00	0.00
Ce	0.00	0.00	0.00	0.00	0.00	0.00	0.00	0.00	0.00	0.00	0.00	0.00	0.00	0.00	0.00	0.00	0.00
Pr	0.00	0.00	0.00	0.00	0.00	0.00	0.00	0.00	0.00	0.00	0.00	0.00	0.00	0.00	0.00	0.00	0.00
Nd	0.00	0.00	0.00	0.00	0.01	0.00	0.00	0.00	0.00	0.00	0.00	0.00	0.00	0.00	0.00	0.00	0.00
Sm	0.00	0.00	0.00	0.00	0.00	0.00	0.00	0.00	0.00	0.00	0.00	0.00	0.00	0.00	0.00	0.00	0.00
Gd	0.00	0.00	0.00	0.00	0.00	0.00	0.00	0.00	0.00	0.00	0.00	0.00	0.00	0.00	0.00	0.00	0.00
Dy	0.00	0.00	0.00	0.00	0.00	0.00	0.00	0.00	0.00	0.00	0.00	0.00	0.00	0.00	0.00	0.00	0.00
Ca	4.97	5.07	4.94	4.93	5.00	4.96	5.03	4.95	4.97	5.00	4.98	5.05	5.05	5.00	4.93	5.01	4.98
Fe	0.01	0.00	0.00	0.01	0.01	0.01	0.02	0.00	0.01	0.01	0.02	0.01	0.01	0.01	0.01	0.01	0.01
Mn	0.00	0.00	0.00	0.00	0.01	0.00	0.00	0.00	0.00	0.00	0.00	0.01	0.00	0.00	0.00	0.00	0.00
Sr	0.00	0.00	0.00	0.00	0.00	0.00	0.00	0.00	0.00	0.00	0.00	0.00	0.00	0.00	0.00	0.00	0.00
Na	0.00	0.00	0.00	0.00	0.00	0.00	0.00	0.00	0.00	0.00	0.00	0.00	0.00	0.00	0.00	0.00	0.00
F	0.95	0.96	0.98	0.95	0.99	0.99	0.91	0.99	0.99	0.92	1.00	1.00	0.85	0.97	1.03	0.99	0.97
Cl	0.00	0.00	0.00	0.00	0.00	0.00	0.00	0.00	0.00	0.00	0.00	0.00	0.00	0.00	0.00	0.00	0.00
OH	0.06	0.03	0.06	0.06	0.03	0.06	0.09	0.06	0.06	0.08	0.06	0.06	0.15	0.03	0.04	0.06	0.02
Total	1.01	1.00	1.03	1.01	1.02	1.05	1.00	1.05	1.04	1.00	1.05	1.05	1.00	1.00	1.08	1.05	1.00
XF/XOH	16.60	27.75	17.22	16.88	34.99	17.57	9.68	17.67	17.56	11.61	17.66	17.40	5.86	31.29	22.99	17.49	18.76
XCl/XOH	0.04	0.06	0.04	0.04	0.07	0.03	0.02	0.02	0.02	0.01	0.01	0.01	0.00	0.01	0.00	0.00	0.08

Page 11														
Charnockite sample	9													9
No. on map	12													12
Sample No.	NIL25-17													NIL25-17
No of grains analysed	1	2	3	4	5	6	7	8	9	10	11	12	13	14	14
														Mean
P_2O_5	41.45	41.11	41.02	41.36	41.53	40.22	41.46	41.31	40.90	40.84	41.48	41.02	41.03	41.20	41.14
SiO_2	0.17	0.12	0.14	0.13	0.13	0.14	0.15	0.15	0.13	0.19	0.15	0.14	0.15	0.15	0.14
Al_2O_3	0.00	0.00	0.00	0.00	0.00	0.00	0.00	0.00	0.00	0.00	0.00	0.00	0.00	0.00	0.00
SO_2	0.00	0.01	0.04	0.01	0.01	0.01	0.00	0.00	0.01	0.00	0.00	0.00	0.00	0.00	0.01
Y_2O_3	0.01	0.04	0.02	0.05	0.00	0.05	0.04	0.03	0.00	0.05	0.01	0.04	0.00	0.03	0.03
La_2O_3	0.00	0.00	0.00	0.00	0.02	0.02	0.01	0.00	0.00	0.03	0.03	0.02	0.00	0.21	0.02
Ce_2O_3	0.09	0.05	0.02	0.08	0.02	0.12	0.02	0.05	0.00	0.16	0.05	0.08	0.00	0.72	0.10
Pr_2O_3	0.01	0.03	0.00	0.03	0.00	0.02	0.00	0.00	0.00	0.00	0.01	0.03	0.02	0.08	0.02
Nd_2O_3	0.14	0.09	0.02	0.10	0.05	0.15	0.02	0.07	0.03	0.23	0.04	0.13	0.04	0.54	0.12
Sm_2O_3	0.05	0.07	0.02	0.04	0.01	0.10	0.14	0.02	0.05	0.07	0.12	0.07	0.00	0.04	0.06
Gd_2O_3	0.09	0.00	0.00	0.00	0.02	0.00	0.04	0.00	0.00	0.01	0.10	0.03	0.01	0.14	0.03
Dy_2O_3	0.00	0.09	0.00	0.00	0.02	0.00	0.01	0.00	0.00	0.00	0.00	0.01	0.04	0.00	0.01
CaO	54.76	54.83	54.67	54.83	55.00	53.60	54.76	55.09	54.44	54.31	55.06	54.43	54.87	53.78	54.60
FeO	0.33	0.32	0.06	0.42	0.14	0.82	0.23	0.09	0.28	0.30	0.21	0.69	0.09	0.50	0.32
MnO	0.01	0.03	0.00	0.00	0.02	0.01	0.00	0.02	0.02	0.00	0.01	0.02	0.00	0.00	0.01
SrO	0.04	0.01	0.01	0.00	0.00	0.04	0.00	0.05	0.00	0.07	0.02	0.03	0.00	0.00	0.02
Na_2O	0.00	0.00	0.00	0.00	0.00	0.01	0.01	0.00	0.00	0.01	0.00	0.01	0.01	0.00	0.00
F	3.72	3.79	3.75	3.73	3.73	3.44	3.73	3.57	3.66	3.53	3.62	3.67	3.72	3.51	3.65
Cl	0.08	0.07	0.07	0.05	0.05	0.05	0.05	0.04	0.04	0.03	0.03	0.03	0.02	0.02	0.04
H_2O	0.10	0.10	0.10	0.10	0.10	0.10	0.10	0.10	0.10	0.10	0.10	0.10	0.10	0.10	0.10
(F+Cl+OH)	0.00	0.00	0.00	0.00	0.00	0.00	0.00	0.00	0.00	0.00	0.00	0.00	0.00	0.00	0.00
sum	101.04	100.78	99.95	100.94	100.84	98.89	100.76	100.58	99.64	99.91	101.03	100.54	100.10	101.01	100.43
O=(F+Cl)	1.58	1.61	1.59	1.58	1.58	1.46	1.58	1.51	1.55	1.49	1.53	1.55	1.57	1.48	1.55
total	99.46	99.17	98.36	99.36	99.26	97.43	99.17	99.07	98.09	98.41	99.50	98.99	98.53	99.53	98.88
(Y+REE)	0.39	0.38	0.08	0.29	0.15	0.46	0.28	0.17	0.08	0.53	0.35	0.40	0.11	1.75	0.39
P	2.97	2.96	2.97	2.97	2.98	2.96	2.98	2.97	2.97	2.97	2.97	2.96	2.96	2.98	2.97
Si	0.01	0.01	0.01	0.01	0.01	0.01	0.01	0.01	0.01	0.02	0.01	0.01	0.01	0.01	0.01
Al	0.00	0.00	0.00	0.00	0.00	0.00	0.00	0.00	0.00	0.00	0.00	0.00	0.00	0.00	0.00
S	0.00	0.00	0.01	0.00	0.00	0.00	0.00	0.00	0.00	0.00	0.00	0.00	0.00	0.00	0.00
Y	0.00	0.00	0.00	0.00	0.00	0.00	0.00	0.00	0.00	0.00	0.00	0.00	0.00	0.00	0.00
La	0.00	0.00	0.00	0.00	0.00	0.00	0.00	0.00	0.00	0.00	0.00	0.00	0.00	0.01	0.00
Ce	0.00	0.00	0.00	0.00	0.00	0.00	0.00	0.00	0.00	0.00	0.00	0.00	0.00	0.02	0.00
Pr	0.00	0.00	0.00	0.00	0.00	0.00	0.00	0.00	0.00	0.00	0.00	0.00	0.00	0.00	0.00
Nd	0.00	0.00	0.00	0.00	0.00	0.00	0.00	0.00	0.00	0.01	0.00	0.00	0.00	0.02	0.00
Sm	0.00	0.00	0.00	0.00	0.00	0.00	0.00	0.00	0.00	0.00	0.00	0.00	0.00	0.00	0.00
Gd	0.00	0.00	0.00	0.00	0.00	0.00	0.00	0.00	0.00	0.00	0.00	0.00	0.00	0.00	0.00
Dy	0.00	0.00	0.00	0.00	0.00	0.00	0.00	0.00	0.00	0.00	0.00	0.00	0.00	0.00	0.00
Ca	4.97	4.99	5.00	4.98	4.99	4.99	4.97	5.02	5.00	4.99	4.99	4.97	5.02	4.91	4.99
Fe	0.05	0.05	0.01	0.06	0.02	0.12	0.03	0.01	0.04	0.04	0.03	0.10	0.01	0.07	0.05
Mn	0.00	0.00	0.00	0.00	0.00	0.00	0.00	0.00	0.00	0.00	0.00	0.00	0.00	0.00	0.00
Sr	0.00	0.00	0.00	0.00	0.00	0.00	0.00	0.00	0.00	0.00	0.00	0.00	0.00	0.00	0.00
Na	0.00	0.00	0.00	0.00	0.00	0.00	0.00	0.00	0.00	0.00	0.00	0.00	0.00	0.00	0.00
F	1.00	1.02	1.01	1.00	1.00	0.94	1.00	0.96	0.99	0.96	0.97	0.99	1.00	0.95	0.98
Cl	0.01	0.01	0.01	0.01	0.01	0.01	0.01	0.01	0.01	0.00	0.00	0.00	0.00	0.00	0.01
OH	0.06	0.06	0.06	0.06	0.06	0.06	0.06	0.06	0.06	0.06	0.06	0.06	0.06	0.06	0.06
Total	1.06	1.09	1.08	1.06	1.06	1.01	1.06	1.02	1.05	1.02	1.03	1.05	1.06	1.01	1.05
XF/XOH	17.62	17.99	17.76	17.66	17.67	16.30	17.67	16.91	17.33	16.72	17.15	17.38	17.64	16.64	17.32
XCl/XOH	0.19	0.19	0.17	0.14	0.12	0.12	0.12	0.11	0.09	0.08	0.07	0.07	0.06	0.04	0.11

Page 12												
Charnockite sample	10											*10*
No. on map	54											*54*
Sample No.	NIL3-9											NIL3-9
No of grains analysed	1	2	3	4	5	6	7	8	9	10	11	12	12
												Mean
P_2O_5	42.90	41.97	41.22	41.91	42.10	41.17	42.15	41.47	41.75	41.97	42.20	42.30	*41.92*
SiO_2	0.00	0.16	0.11	0.13	0.18	0.14	0.17	0.14	0.13	0.12	0.15	0.17	*0.13*
Al_2O_3	0.00	0.00	0.00	0.00	0.00	0.00	0.00	0.00	0.00	0.00	0.00	0.00	*0.00*
SO_2	0.00	0.00	0.02	0.01	0.00	0.00	0.01	0.00	0.01	0.01	0.00	0.01	*0.01*
Y_2O_3	0.00	0.07	0.06	0.02	0.01	0.00	0.00	0.00	0.00	0.00	0.01	0.00	*0.01*
La_2O_3	0.00	0.00	0.00	0.00	0.01	0.00	0.01	0.53	0.00	0.00	0.00	0.01	*0.05*
Ce_2O_3	0.03	0.06	0.06	0.14	0.05	0.05	0.11	1.75	0.04	0.20	0.03	0.04	*0.21*
Pr_2O_3	0.00	0.04	0.04	0.05	0.02	0.00	0.04	0.29	0.00	0.03	0.03	0.04	*0.05*
Nd_2O_3	0.02	0.12	0.08	0.21	0.06	0.08	0.11	1.06	0.06	0.25	0.11	0.14	*0.19*
Sm_2O_3	0.00	0.06	0.07	0.05	0.06	0.04	0.00	0.10	0.02	0.08	0.04	0.05	*0.05*
Gd_2O_3	0.02	0.00	0.08	0.12	0.02	0.00	0.01	0.10	0.03	0.00	0.03	0.00	*0.04*
Dy_2O_3	0.00	0.00	0.00	0.00	0.00	0.00	0.02	0.05	0.00	0.00	0.02	0.00	*0.01*
CaO	57.70	55.58	54.35	55.02	55.70	54.48	55.57	53.12	55.50	55.37	55.49	55.91	*55.32*
FeO	0.41	0.45	0.84	0.52	0.10	0.75	0.15	0.07	0.41	0.56	0.67	0.30	*0.44*
MnO	0.00	0.01	0.02	0.02	0.00	0.01	0.01	0.00	0.03	0.01	0.01	0.01	*0.01*
SrO	0.00	0.00	0.00	0.07	0.00	0.05	0.03	0.02	0.02	0.06	0.04	0.05	*0.03*
Na_2O	0.03	0.01	0.00	0.01	0.00	0.00	0.00	0.00	0.00	0.01	0.00	0.00	*0.01*
F	3.82	3.83	3.77	3.79	3.83	3.67	3.81	3.55	3.70	3.64	3.45	3.63	*3.71*
Cl	0.06	0.03	0.02	0.02	0.02	0.02	0.01	0.01	0.01	0.01	0.01	0.01	*0.02*
H_2O	0.09	0.10	0.10	0.10	0.10	0.10	0.10	0.10	0.10	0.10	0.10	0.10	*0.10*
(F+Cl+OH)	0.00	0.00	0.00	0.00	0.00	0.00	0.00	0.00	0.00	0.00	0.00	0.00	*0.00*
sum	105.07	102.47	100.84	102.18	102.27	100.57	102.32	102.35	101.81	102.40	102.38	102.78	*102.29*
O=(F+Cl)	1.62	1.62	1.59	1.60	1.62	1.55	1.61	1.50	1.56	1.53	1.45	1.53	*1.56*
total	103.45	100.86	99.25	100.58	100.66	99.02	100.71	100.85	100.25	100.86	100.92	101.25	*100.72*
(Y+REE)	0.07	0.34	0.39	0.59	0.24	0.18	0.31	3.87	0.15	0.55	0.27	0.28	*0.60*
P	2.96	2.97	2.97	2.97	2.97	2.97	2.98	2.98	2.97	2.97	2.99	2.98	*2.97*
Si	0.00	0.01	0.01	0.01	0.02	0.01	0.01	0.01	0.01	0.01	0.01	0.01	*0.01*
Al	0.00	0.00	0.00	0.00	0.00	0.00	0.00	0.00	0.00	0.00	0.00	0.00	*0.00*
S	0.00	0.00	0.00	0.00	0.00	0.00	0.00	0.00	0.00	0.00	0.00	0.00	*0.00*
Y	0.00	0.00	0.00	0.00	0.00	0.00	0.00	0.00	0.00	0.00	0.00	0.00	*0.00*
La	0.00	0.00	0.00	0.00	0.00	0.00	0.00	0.02	0.00	0.00	0.00	0.00	*0.00*
Ce	0.00	0.00	0.00	0.00	0.00	0.00	0.00	0.05	0.00	0.01	0.00	0.00	*0.01*
Pr	0.00	0.00	0.00	0.00	0.00	0.00	0.00	0.00	0.00	0.00	0.00	0.00	*0.00*
Nd	0.00	0.00	0.00	0.01	0.00	0.00	0.00	0.03	0.00	0.01	0.00	0.00	*0.01*
Sm	0.00	0.00	0.00	0.00	0.00	0.00	0.00	0.00	0.00	0.00	0.00	0.00	*0.00*
Gd	0.00	0.00	0.00	0.00	0.00	0.00	0.00	0.00	0.00	0.00	0.00	0.00	*0.00*
Dy	0.00	0.00	0.00	0.00	0.00	0.00	0.00	0.00	0.00	0.00	0.00	0.00	*0.00*
Ca	5.04	4.97	4.95	4.94	4.98	4.97	4.97	4.83	5.00	4.97	4.97	4.98	*4.96*
Fe	0.06	0.06	0.12	0.07	0.01	0.11	0.02	0.01	0.06	0.08	0.09	0.04	*0.06*
Mn	0.00	0.00	0.00	0.00	0.00	0.00	0.00	0.00	0.00	0.00	0.00	0.00	*0.00*
Sr	0.00	0.00	0.00	0.00	0.00	0.00	0.00	0.00	0.00	0.00	0.00	0.00	*0.00*
Na	0.00	0.00	0.00	0.00	0.00	0.00	0.00	0.00	0.00	0.00	0.00	0.00	*0.00*
F	0.98	1.01	1.01	1.00	1.01	0.99	1.00	0.95	0.98	0.96	0.91	0.96	*0.98*
Cl	0.01	0.00	0.00	0.00	0.00	0.00	0.00	0.00	0.00	0.00	0.00	0.00	*0.00*
OH	0.05	0.06	0.06	0.06	0.06	0.06	0.06	0.06	0.06	0.06	0.06	0.06	*0.06*
Total	1.04	1.07	1.07	1.06	1.07	1.05	1.06	1.01	1.04	1.02	0.97	1.01	*1.04*
XF/XOH	20.11	18.14	17.87	17.95	18.16	17.40	18.04	16.85	17.54	17.25	16.34	17.23	*17.74*
XCl/XOH	0.17	0.07	0.06	0.05	0.05	0.04	0.03	0.03	0.03	0.03	0.02	0.02	*0.05*

Page 13							
Charnockite sample	11						*11*
No. on map	57						*57*
Sample No.	NIL3-16						NIL3-16
No of grains analysed	1	2	3	4	5	6	6
							Mean
P_2O_5	41.67	42.41	41.18	41.14	41.34	40.55	*41.38*
SiO_2	0.20	0.16	0.13	0.16	0.13	0.13	*0.15*
Al_2O_3	0.00	0.00	0.00	0.00	0.00	0.00	*0.00*
SO_2	0.00	0.02	0.01	0.02	0.01	0.01	*0.01*
Y_2O_3	0.01	0.04	0.04	0.01	0.00	0.00	*0.02*
La_2O_3	0.01	0.01	0.00	0.00	0.03	0.00	*0.01*
Ce_2O_3	0.05	0.02	0.08	0.11	0.00	0.00	*0.04*
Pr_2O_3	0.00	0.05	0.00	0.00	0.00	0.03	*0.01*
Nd_2O_3	0.02	0.09	0.11	0.11	0.03	0.02	*0.06*
Sm_2O_3	0.00	0.00	0.00	0.00	0.06	0.07	*0.02*
Gd_2O_3	0.06	0.06	0.00	0.08	0.08	0.00	*0.05*
Dy_2O_3	0.00	0.04	0.00	0.00	0.02	0.00	*0.01*
CaO	54.01	58.22	53.90	53.89	53.88	53.85	*54.62*
FeO	0.34	0.88	0.41	0.08	0.07	0.08	*0.31*
MnO	0.01	0.03	0.01	0.00	0.01	0.01	*0.01*
SrO	0.04	0.00	0.02	0.01	0.00	0.00	*0.01*
Na_2O	0.01	0.00	0.01	0.01	0.01	0.01	*0.01*
F	3.58	3.93	3.72	3.72	3.71	3.74	*3.73*
Cl	0.40	0.13	0.08	0.06	0.09	0.08	*0.14*
H_2O	0.10	0.10	0.10	0.10	0.10	0.10	*0.10*
(F+Cl+OH)	0.00	0.00	0.00	0.00	0.00	0.00	*0.00*
sum	100.50	106.18	99.79	99.49	99.56	98.68	*100.70*
O=(F+Cl)	1.60	1.69	1.58	1.58	1.58	1.59	*1.60*
total	98.90	104.49	98.21	97.91	97.98	97.09	*99.10*
(Y+REE)	0.15	0.30	0.22	0.30	0.22	0.13	*0.22*
P	2.99	2.92	2.98	2.98	2.99	2.97	*2.97*
Si	0.02	0.01	0.01	0.01	0.01	0.01	*0.01*
Al	0.00	0.00	0.00	0.00	0.00	0.00	*0.00*
S	0.00	0.00	0.00	0.00	0.00	0.00	*0.00*
Y	0.00	0.00	0.00	0.00	0.00	0.00	*0.00*
La	0.00	0.00	0.00	0.00	0.00	0.00	*0.00*
Ce	0.00	0.00	0.00	0.00	0.00	0.00	*0.00*
Pr	0.00	0.00	0.00	0.00	0.00	0.00	*0.00*
Nd	0.00	0.00	0.00	0.00	0.00	0.00	*0.00*
Sm	0.00	0.00	0.00	0.00	0.00	0.00	*0.00*
Gd	0.00	0.00	0.00	0.00	0.00	0.00	*0.00*
Dy	0.00	0.00	0.00	0.00	0.00	0.00	*0.00*
Ca	4.91	5.06	4.94	4.95	4.94	4.99	*4.97*
Fe	0.05	0.12	0.06	0.01	0.01	0.01	*0.04*
Mn	0.00	0.00	0.00	0.00	0.00	0.00	*0.00*
Sr	0.00	0.00	0.00	0.00	0.00	0.00	*0.00*
Na	0.00	0.00	0.00	0.00	0.00	0.00	*0.00*
F	0.96	1.01	1.01	1.01	1.00	1.02	*1.00*
Cl	0.06	0.02	0.01	0.01	0.01	0.01	*0.02*
OH	0.06	0.05	0.06	0.06	0.06	0.06	*0.06*
Total	1.07	1.08	1.07	1.07	1.07	1.09	*1.08*
XF/XOH	16.95	18.64	17.62	17.65	17.60	17.71	*17.69*
XCl/XOH	1.01	0.34	0.19	0.15	0.22	0.20	*0.35*

Page 14										
Charnockite sample	15									*15*
No. on map	57									*57*
Sample No.	NIL3-16									NIL3-16
No of grains analysed	1	2	3	4	5	6	7	8	9	9
										Mean
P_2O_5	41.81	42.00	40.99	41.96	42.67	42.23	42.19	42.71	41.16	*41.97*
SiO_2	0.00	0.00	0.13	0.00	0.00	0.00	0.00	0.00	0.00	*0.01*
Al_2O_3	0.00	0.00	0.00	0.00	0.00	0.00	0.00	0.00	0.00	*0.00*
SO_2	0.04	0.04	0.02	0.03	0.04	0.00	0.00	0.00	0.01	*0.02*
Y_2O_3	0.00	0.00	0.00	0.00	0.00	0.00	0.00	0.00	0.00	*0.00*
La_2O_3	0.00	0.01	0.00	0.00	0.17	0.01	0.04	0.00	0.00	*0.03*
Ce_2O_3	0.06	0.03	0.02	0.03	0.35	0.02	0.02	0.01	0.02	*0.06*
Pr_2O_3	0.11	0.00	0.19	0.00	0.09	0.00	0.06	0.12	0.05	*0.07*
Nd_2O_3	0.09	0.08	0.07	0.10	0.16	0.03	0.00	0.01	0.05	*0.07*
Sm_2O_3	0.03	0.08	0.04	0.00	0.05	0.05	0.05	0.01	0.00	*0.03*
Gd_2O_3	0.00	0.11	0.00	0.05	0.08	0.10	0.02	0.00	0.00	*0.04*
Dy_2O_3	0.06	0.00	0.03	0.02	0.00	0.00	0.04	0.03	0.00	*0.02*
CaO	56.42	56.24	55.27	57.00	55.96	56.48	57.03	56.67	55.83	*56.32*
FeO	0.16	0.21	0.59	0.38	0.06	0.07	0.10	0.11	0.45	*0.24*
MnO	0.00	0.00	0.00	0.00	0.00	0.00	0.00	0.00	0.00	*0.00*
SrO	0.00	0.00	0.00	0.00	0.00	0.00	0.00	0.00	0.00	*0.00*
Na_2O	0.00	0.00	0.00	0.00	0.00	0.00	0.00	0.01	0.00	*0.00*
F	3.53	3.57	3.50	3.60	3.57	3.57	3.65	3.52	3.57	*3.56*
Cl	0.23	0.05	0.07	0.07	0.15	0.06	0.02	0.02	0.02	*0.08*
H_2O	0.10	0.10	0.10	0.10	0.10	0.10	0.10	0.10	0.10	*0.10*
(F+Cl+OH)	0.00	0.00	0.00	0.00	0.00	0.00	0.00	0.00	0.00	*0.00*
sum	102.61	102.50	101.01	103.34	103.45	102.71	103.32	103.33	101.25	*102.61*
O=(F+Cl)	1.54	1.51	1.49	1.53	1.54	1.52	1.54	1.48	1.51	*1.52*
total	101.07	100.99	99.52	101.81	101.91	101.19	101.78	101.84	99.74	*101.10*
(Y+REE)	0.34	0.30	0.34	0.20	0.90	0.20	0.23	0.18	0.12	*0.31*
P	2.96	2.97	2.95	2.95	2.99	2.98	2.96	2.99	2.95	*2.97*
Si	0.00	0.00	0.01	0.00	0.00	0.00	0.00	0.00	0.00	*0.00*
Al	0.00	0.00	0.00	0.00	0.00	0.00	0.00	0.00	0.00	*0.00*
S	0.00	0.00	0.00	0.00	0.01	0.00	0.00	0.00	0.00	*0.00*
Y	0.00	0.00	0.00	0.00	0.00	0.00	0.00	0.00	0.00	*0.00*
La	0.00	0.00	0.00	0.00	0.01	0.00	0.00	0.00	0.00	*0.00*
Ce	0.00	0.00	0.00	0.00	0.01	0.00	0.00	0.00	0.00	*0.00*
Pr	0.00	0.00	0.00	0.00	0.00	0.00	0.00	0.00	0.00	*0.00*
Nd	0.00	0.00	0.00	0.00	0.00	0.00	0.00	0.00	0.00	*0.00*
Sm	0.00	0.00	0.00	0.00	0.00	0.00	0.00	0.00	0.00	*0.00*
Gd	0.00	0.00	0.00	0.00	0.00	0.00	0.00	0.00	0.00	*0.00*
Dy	0.00	0.00	0.00	0.00	0.00	0.00	0.00	0.00	0.00	*0.00*
Ca	5.06	5.03	5.03	5.07	4.96	5.04	5.07	5.02	5.07	*5.04*
Fe	0.02	0.03	0.08	0.05	0.01	0.01	0.01	0.02	0.06	*0.03*
Mn	0.00	0.00	0.00	0.00	0.00	0.00	0.00	0.00	0.00	*0.00*
Sr	0.00	0.00	0.00	0.00	0.00	0.00	0.00	0.00	0.00	*0.00*
Na	0.00	0.00	0.00	0.00	0.00	0.00	0.00	0.00	0.00	*0.00*
F	0.93	0.94	0.94	0.95	0.93	0.94	0.96	0.92	0.96	*0.94*
Cl	0.03	0.01	0.01	0.01	0.02	0.01	0.00	0.00	0.00	*0.01*
OH	0.06	0.06	0.06	0.06	0.06	0.06	0.06	0.06	0.06	*0.06*
Total	1.02	1.01	1.01	1.01	1.01	1.00	1.02	0.98	1.02	*1.01*
XF/XOH	16.72	16.90	16.61	17.08	16.90	16.93	17.31	16.66	16.93	*16.89*
XCl/XOH	0.59	0.13	0.17	0.18	0.39	0.15	0.05	0.05	0.04	*0.19*

Page 15										
Charnockite sample	12									*12*
No. on map										
Sample No.	NIL3-17									NIL3-17
No of grains analysed	1	2	3	4	5	6	7	8	9	9
										Mean
P_2O_5	41.22	41.37	40.83	41.82	41.11	40.74	42.91	42.05	40.27	*41.37*
SiO_2	0.12	0.05	0.14	0.06	0.14	0.09	0.17	0.05	0.18	*0.11*
Al_2O_3	0.00	0.00	0.00	0.00	0.00	0.00	0.00	0.00	0.00	*0.00*
SO_2	0.01	0.00	0.01	0.01	0.02	0.01	0.00	0.01	0.01	*0.01*
Y_2O_3	0.00	0.00	0.00	0.00	0.00	0.00	0.00	0.00	0.00	*0.00*
La_2O_3	0.00	0.02	0.00	0.03	0.01	0.00	0.02	0.02	0.00	*0.01*
Ce_2O_3	0.07	0.05	0.10	0.07	0.03	0.02	0.09	0.00	0.00	*0.05*
Pr_2O_3	0.06	0.00	0.02	0.06	0.00	0.02	0.00	0.00	0.04	*0.02*
Nd_2O_3	0.15	0.03	0.14	0.06	0.09	0.06	0.03	0.03	0.07	*0.07*
Sm_2O_3	0.00	0.00	0.00	0.00	0.00	0.00	0.00	0.00	0.00	*0.00*
Gd_2O_3	0.00	0.00	0.00	0.00	0.00	0.00	0.00	0.00	0.00	*0.00*
Dy_2O_3	0.00	0.00	0.00	0.00	0.00	0.00	0.00	0.00	0.00	*0.00*
CaO	55.27	55.47	54.65	55.36	55.05	55.21	55.32	55.63	54.73	*55.19*
FeO	0.36	0.19	0.34	0.17	0.05	0.72	0.11	0.09	0.20	*0.25*
MnO	0.00	0.00	0.03	0.07	0.02	0.12	0.00	0.00	0.12	*0.04*
SrO	0.00	0.00	0.00	0.00	0.00	0.00	0.00	0.00	0.00	*0.00*
Na_2O	0.00	0.00	0.00	0.00	0.00	0.00	0.00	0.02	0.02	*0.00*
F	3.48	3.48	3.45	3.48	3.41	3.55	3.53	3.53	3.60	*3.50*
Cl	0.04	0.03	0.03	0.02	0.02	0.02	0.02	0.01	0.01	*0.02*
H_2O	0.10	0.10	0.10	0.10	0.10	0.10	0.10	0.10	0.10	*0.10*
(F+Cl+OH)	0.00	0.00	0.00	0.00	0.00	0.00	0.00	0.00	0.00	*0.00*
sum	100.88	100.79	99.84	101.31	100.05	100.66	102.30	101.54	99.35	*100.75*
O=(F+Cl)	1.47	1.47	1.46	1.47	1.44	1.50	1.49	1.49	1.52	*1.48*
total	99.41	99.32	98.38	99.84	98.61	99.16	100.81	100.05	97.83	*99.27*
(Y+REE)	0.28	0.10	0.26	0.22	0.13	0.10	0.14	0.05	0.11	*0.15*
P	2.96	2.97	2.97	2.99	2.97	2.94	3.02	2.99	2.94	*2.97*
Si	0.01	0.00	0.01	0.01	0.01	0.01	0.01	0.00	0.02	*0.01*
Al	0.00	0.00	0.00	0.00	0.00	0.00	0.00	0.00	0.00	*0.00*
S	0.00	0.00	0.00	0.00	0.00	0.00	0.00	0.00	0.00	*0.00*
Y	0.00	0.00	0.00	0.00	0.00	0.00	0.00	0.00	0.00	*0.00*
La	0.00	0.00	0.00	0.00	0.00	0.00	0.00	0.00	0.00	*0.00*
Ce	0.00	0.00	0.00	0.00	0.00	0.00	0.00	0.00	0.00	*0.00*
Pr	0.00	0.00	0.00	0.00	0.00	0.00	0.00	0.00	0.00	*0.00*
Nd	0.00	0.00	0.00	0.00	0.00	0.00	0.00	0.00	0.00	*0.00*
Sm	0.00	0.00	0.00	0.00	0.00	0.00	0.00	0.00	0.00	*0.00*
Gd	0.00	0.00	0.00	0.00	0.00	0.00	0.00	0.00	0.00	*0.00*
Dy	0.00	0.00	0.00	0.00	0.00	0.00	0.00	0.00	0.00	*0.00*
Ca	5.03	5.04	5.02	5.00	5.04	5.05	4.92	5.01	5.06	*5.02*
Fe	0.05	0.03	0.05	0.02	0.01	0.10	0.02	0.01	0.03	*0.04*
Mn	0.00	0.00	0.00	0.01	0.00	0.02	0.00	0.00	0.02	*0.01*
Sr	0.00	0.00	0.00	0.00	0.00	0.00	0.00	0.00	0.00	*0.00*
Na	0.00	0.00	0.00	0.00	0.00	0.00	0.00	0.00	0.00	*0.00*
F	0.93	0.93	0.94	0.93	0.92	0.96	0.93	0.94	0.98	*0.94*
Cl	0.01	0.00	0.00	0.00	0.00	0.00	0.00	0.00	0.00	*0.00*
OH	0.06	0.06	0.06	0.06	0.06	0.06	0.06	0.06	0.06	*0.06*
Total	1.00	0.99	1.00	0.99	0.98	1.02	0.99	1.00	1.04	*1.00*
XF/XOH	16.50	16.50	16.36	16.50	16.17	16.83	16.73	16.73	17.07	*16.60*
XCl/XOH	0.10	0.08	0.08	0.05	0.05	0.05	0.05	0.03	0.03	*0.06*

Page 16																			
Charnockite sample	13																		*13*
No. on map	58																		*58*
Sample No.	NIL3-19																		NIL3-19
No of grains analysed	1	2	3	4	5	6	7	8	9	10	11	12	13	14	15	16	17	18	18
																			Mean
P_2O_5	40.15	41.18	40.85	42.03	41.19	41.13	41.96	41.53	41.94	42.65	41.41	42.07	41.92	42.27	42.08	41.47	41.79	41.93	*41.64*
SiO_2	0.24	0.23	0.21	0.25	0.25	0.24	0.26	0.24	0.22	0.26	0.22	0.21	0.21	0.22	0.22	0.21	0.21	0.25	*0.23*
Al_2O_3	0.00	0.00	0.00	0.00	0.00	0.00	0.00	0.00	0.00	0.00	0.00	0.00	0.00	0.00	0.00	0.00	0.00	0.00	*0.00*
SO_2	0.02	0.02	0.02	0.00	0.00	0.00	0.01	0.00	0.01	0.02	0.01	0.01	0.02	0.01	0.00	0.01	0.01	0.02	*0.01*
Y_2O_3	0.03	0.02	0.00	0.01	0.01	0.00	0.00	0.03	0.00	0.00	0.01	0.00	0.02	0.00	0.01	0.05	0.00	0.09	*0.01*
La_2O_3	0.00	0.03	0.01	0.00	0.01	0.00	0.00	0.00	0.00	0.00	0.00	0.00	0.01	0.00	0.00	0.04	0.00	0.01	*0.01*
Ce_2O_3	0.03	0.05	0.07	0.04	0.01	0.02	0.04	0.03	0.00	0.02	0.00	0.01	0.06	0.01	0.03	0.00	0.00	0.02	*0.02*
Pr_2O_3	0.02	0.02	0.06	0.00	0.00	0.02	0.00	0.02	0.00	0.00	0.03	0.03	0.01	0.00	0.01	0.03	0.00	0.00	*0.01*
Nd_2O_3	0.06	0.04	0.02	0.06	0.04	0.00	0.06	0.06	0.03	0.04	0.06	0.06	0.08	0.02	0.04	0.01	0.06	0.06	*0.04*
Sm_2O_3	0.03	0.04	0.05	0.05	0.00	0.00	0.00	0.08	0.04	0.08	0.08	0.00	0.00	0.00	0.00	0.01	0.00	0.07	*0.03*
Gd_2O_3	0.01	0.07	0.05	0.02	0.00	0.00	0.09	0.00	0.00	0.04	0.00	0.06	0.03	0.00	0.02	0.12	0.04	0.00	*0.03*
Dy_2O_3	0.00	0.00	0.02	0.02	0.05	0.00	0.03	0.05	0.00	0.07	0.00	0.01	0.00	0.02	0.04	0.02	0.02	0.02	*0.02*
CaO	52.44	53.76	52.94	54.96	53.47	53.70	54.06	53.90	54.92	55.10	53.68	54.14	54.62	54.44	54.89	54.20	54.36	54.45	*54.11*
FeO	0.24	0.03	0.03	0.05	0.05	0.06	0.03	0.03	0.04	0.02	0.28	0.04	0.04	0.03	0.03	0.04	0.10	0.11	*0.07*
MnO	0.01	0.00	0.00	0.02	0.03	0.00	0.00	0.01	0.00	0.01	0.00	0.00	0.01	0.01	0.00	0.02	0.00	0.03	*0.01*
SrO	0.05	0.05	0.00	0.01	0.01	0.01	0.00	0.01	0.00	0.00	0.01	0.02	0.01	0.00	0.01	0.00	0.01	0.01	*0.01*
Na_2O	0.01	0.00	0.01	0.00	0.01	0.01	0.00	0.01	0.00	0.00	0.01	0.00	0.01	0.00	0.01	0.01	0.01	0.00	*0.01*
F	3.60	3.71	3.32	3.81	3.48	3.68	3.73	3.65	3.86	3.75	3.66	3.74	3.74	3.66	3.65	3.67	3.67	3.63	*3.67*
Cl	0.03	0.03	0.03	0.03	0.03	0.03	0.03	0.02	0.02	0.02	0.02	0.02	0.02	0.02	0.02	0.02	0.01	0.01	*0.02*
H_2O	0.10	0.10	0.10	0.10	0.10	0.10	0.10	0.10	0.10	0.10	0.10	0.10	0.10	0.10	0.10	0.10	0.10	0.10	*0.10*
(F+Cl+OH)	0.00	0.00	0.00	0.00	0.00	0.00	0.00	0.00	0.00	0.00	0.00	0.00	0.00	0.00	0.00	0.00	0.00	0.00	*0.00*
sum	97.05	99.38	97.77	101.44	98.73	99.00	100.40	99.76	101.19	102.20	99.56	100.54	100.90	100.79	101.17	100.01	100.39	100.80	*100.06*
O=(F+Cl)	1.52	1.57	1.41	1.61	1.47	1.56	1.58	1.54	1.63	1.58	1.54	1.58	1.58	1.54	1.54	1.55	1.55	1.53	*1.55*
total	95.53	97.81	96.37	99.83	97.26	97.45	98.82	98.22	99.56	100.61	98.01	98.96	99.32	99.25	99.63	98.47	98.84	99.27	*98.51*
(Y+REE)	0.18	0.26	0.27	0.19	0.11	0.05	0.22	0.27	0.07	0.26	0.17	0.18	0.20	0.04	0.15	0.28	0.12	0.27	*0.18*
P	2.98	2.99	3.01	2.99	3.00	2.99	3.01	3.00	2.99	3.00	3.00	3.01	2.99	3.01	3.00	2.99	3.00	3.00	*3.00*
Si	0.02	0.02	0.02	0.02	0.02	0.02	0.02	0.02	0.02	0.02	0.02	0.02	0.02	0.02	0.02	0.02	0.02	0.02	*0.02*
Al	0.00	0.00	0.00	0.00	0.00	0.00	0.00	0.00	0.00	0.00	0.00	0.00	0.00	0.00	0.00	0.00	0.00	0.00	*0.00*
S	0.00	0.00	0.00	0.00	0.00	0.00	0.00	0.00	0.00	0.00	0.00	0.00	0.00	0.00	0.00	0.00	0.00	0.00	*0.00*
Y	0.00	0.00	0.00	0.00	0.00	0.00	0.00	0.00	0.00	0.00	0.00	0.00	0.00	0.00	0.00	0.00	0.00	0.00	*0.00*
La	0.00	0.00	0.00	0.00	0.00	0.00	0.00	0.00	0.00	0.00	0.00	0.00	0.00	0.00	0.00	0.00	0.00	0.00	*0.00*
Ce	0.00	0.00	0.00	0.00	0.00	0.00	0.00	0.00	0.00	0.00	0.00	0.00	0.00	0.00	0.00	0.00	0.00	0.00	*0.00*
Pr	0.00	0.00	0.00	0.00	0.00	0.00	0.00	0.00	0.00	0.00	0.00	0.00	0.00	0.00	0.00	0.00	0.00	0.00	*0.00*
Nd	0.00	0.00	0.00	0.00	0.00	0.00	0.00	0.00	0.00	0.00	0.00	0.00	0.00	0.00	0.00	0.00	0.00	0.00	*0.00*
Sm	0.00	0.00	0.00	0.00	0.00	0.00	0.00	0.00	0.00	0.00	0.00	0.00	0.00	0.00	0.00	0.00	0.00	0.00	*0.00*
Gd	0.00	0.00	0.00	0.00	0.00	0.00	0.00	0.00	0.00	0.00	0.00	0.00	0.00	0.00	0.00	0.00	0.00	0.00	*0.00*
Dy	0.00	0.00	0.00	0.00	0.00	0.00	0.00	0.00	0.00	0.00	0.00	0.00	0.00	0.00	0.00	0.00	0.00	0.00	*0.00*
Ca	4.93	4.94	4.93	4.94	4.93	4.94	4.90	4.92	4.95	4.91	4.92	4.90	4.94	4.91	4.95	4.95	4.93	4.93	*4.93*
Fe	0.03	0.00	0.00	0.01	0.01	0.01	0.00	0.00	0.01	0.00	0.04	0.01	0.01	0.00	0.00	0.01	0.01	0.02	*0.01*
Mn	0.00	0.00	0.00	0.00	0.00	0.00	0.00	0.00	0.00	0.00	0.00	0.00	0.00	0.00	0.00	0.00	0.00	0.00	*0.00*
Sr	0.00	0.00	0.00	0.00	0.00	0.00	0.00	0.00	0.00	0.00	0.00	0.00	0.00	0.00	0.00	0.00	0.00	0.00	*0.00*
Na	0.00	0.00	0.00	0.00	0.00	0.00	0.00	0.00	0.00	0.00	0.00	0.00	0.00	0.00	0.00	0.00	0.00	0.00	*0.00*
F	1.00	1.01	0.91	1.01	0.95	1.00	1.00	0.98	1.03	0.99	0.99	1.00	1.00	0.97	0.97	0.99	0.98	0.97	*0.99*
Cl	0.00	0.00	0.00	0.00	0.00	0.00	0.00	0.00	0.00	0.00	0.00	0.00	0.00	0.00	0.00	0.00	0.00	0.00	*0.00*
OH	0.06	0.06	0.06	0.06	0.06	0.06	0.06	0.06	0.06	0.06	0.06	0.06	0.06	0.06	0.06	0.06	0.06	0.06	*0.06*
Total	1.06	1.07	0.98	1.07	1.01	1.06	1.06	1.05	1.09	1.04	1.05	1.06	1.06	1.03	1.03	1.05	1.04	1.03	*1.05*
XF/XOH	17.05	17.59	15.75	18.06	16.49	17.46	17.69	17.31	18.30	17.77	17.33	17.75	17.74	17.34	17.28	17.39	17.38	17.19	*17.38*
XCl/XOH	0.08	0.08	0.08	0.07	0.07	0.07	0.06	0.06	0.06	0.06	0.05	0.05	0.05	0.05	0.04	0.04	0.03	0.03	*0.06*

Page 17															
Charnockite sample	14														*14*
No. on map	62														*62*
Sample No.	NIL30-15														NIL30-15
No of grains analysed	1	2	3	4	5	6	7	8	9	10	11	12	13	14	14
															Mean
P_2O_5	40.20	40.23	39.96	41.01	40.73	41.13	41.07	40.76	41.53	41.11	41.00	41.51	41.44	41.88	*40.97*
SiO_2	0.13	0.12	0.14	0.09	0.12	0.13	0.10	0.16	0.16	0.12	0.12	0.15	0.14	0.13	*0.13*
Al_2O_3	0.00	0.00	0.00	0.00	0.00	0.00	0.00	0.00	0.00	0.00	0.00	0.00	0.00	0.00	*0.00*
SO_2	0.05	0.04	0.00	0.04	0.04	0.00	0.03	0.02	0.02	0.02	0.03	0.01	0.03	0.01	*0.02*
Y_2O_3	0.00	0.01	0.00	0.04	0.00	0.02	0.01	0.01	0.00	0.02	0.00	0.01	0.00	0.00	*0.01*
La_2O_3	0.01	0.04	0.06	0.01	0.01	0.03	0.00	0.06	0.00	0.02	0.00	0.04	0.02	0.00	*0.02*
Ce_2O_3	0.07	0.06	0.15	0.05	0.18	0.10	0.15	0.16	0.05	0.23	0.11	0.11	0.28	0.05	*0.13*
Pr_2O_3	0.03	0.00	0.02	0.04	0.00	0.00	0.06	0.05	0.01	0.07	0.01	0.00	0.01	0.04	*0.02*
Nd_2O_3	0.09	0.08	0.17	0.06	0.17	0.10	0.18	0.15	0.08	0.27	0.14	0.14	0.21	0.12	*0.14*
Sm_2O_3	0.01	0.03	0.02	0.00	0.07	0.00	0.00	0.00	0.01	0.01	0.00	0.00	0.01	0.00	*0.01*
Gd_2O_3	0.03	0.01	0.00	0.01	0.02	0.09	0.00	0.00	0.00	0.04	0.00	0.03	0.07	0.06	*0.03*
Dy_2O_3	0.01	0.06	0.00	0.02	0.00	0.00	0.00	0.00	0.00	0.03	0.00	0.00	0.04	0.00	*0.01*
CaO	54.23	54.32	54.04	54.38	54.52	54.60	54.54	54.86	55.27	54.53	54.72	54.51	54.50	55.14	*54.58*
FeO	0.05	0.06	0.23	0.09	0.07	0.32	0.04	0.05	0.14	0.32	0.06	0.05	0.36	0.06	*0.13*
MnO	0.01	0.00	0.00	0.00	0.00	0.01	0.00	0.03	0.02	0.00	0.00	0.00	0.00	0.00	*0.00*
SrO	0.03	0.02	0.02	0.00	0.00	0.00	0.00	0.03	0.00	0.05	0.00	0.00	0.06	0.00	*0.01*
Na_2O	0.02	0.02	0.01	0.01	0.01	0.01	0.01	0.01	0.01	0.01	0.01	0.01	0.01	0.00	*0.01*
F	3.42	3.54	3.67	3.53	3.51	3.38	3.64	3.73	3.66	3.54	3.49	3.57	3.67	3.73	*3.58*
Cl	0.05	0.04	0.04	0.02	0.02	0.02	0.02	0.02	0.02	0.02	0.02	0.01	0.01	0.01	*0.02*
H_2O	0.10	0.10	0.10	0.10	0.10	0.10	0.10	0.10	0.10	0.10	0.10	0.10	0.10	0.10	*0.10*
(F+Cl+OH)	0.00	0.00	0.00	0.00	0.00	0.00	0.00	0.00	0.00	0.00	0.00	0.00	0.00	0.00	*0.00*
sum	98.54	98.76	98.65	99.51	99.56	100.04	99.94	100.18	101.07	100.47	99.80	100.25	100.96	101.32	*99.93*
O=(F+Cl)	1.45	1.50	1.56	1.49	1.48	1.43	1.54	1.57	1.54	1.49	1.47	1.51	1.55	1.57	*1.51*
total	97.09	97.26	97.09	98.02	98.08	98.61	98.40	98.60	99.52	98.98	98.33	98.74	99.41	99.75	*98.42*
(Y+REE)	0.25	0.28	0.43	0.23	0.45	0.34	0.39	0.43	0.16	0.67	0.26	0.33	0.63	0.26	*0.37*
P	2.96	2.95	2.94	2.98	2.97	2.98	2.97	2.95	2.97	2.97	2.97	2.99	2.98	2.98	*2.97*
Si	0.01	0.01	0.01	0.01	0.01	0.01	0.01	0.01	0.01	0.01	0.01	0.01	0.01	0.01	*0.01*
Al	0.00	0.00	0.00	0.00	0.00	0.00	0.00	0.00	0.00	0.00	0.00	0.00	0.00	0.00	*0.00*
S	0.01	0.01	0.00	0.01	0.00	0.00	0.00	0.00	0.00	0.00	0.00	0.00	0.00	0.00	*0.00*
Y	0.00	0.00	0.00	0.00	0.00	0.00	0.00	0.00	0.00	0.00	0.00	0.00	0.00	0.00	*0.00*
La	0.00	0.00	0.00	0.00	0.00	0.00	0.00	0.00	0.00	0.00	0.00	0.00	0.00	0.00	*0.00*
Ce	0.00	0.00	0.00	0.00	0.01	0.00	0.00	0.01	0.00	0.01	0.00	0.00	0.01	0.00	*0.00*
Pr	0.00	0.00	0.00	0.00	0.00	0.00	0.00	0.00	0.00	0.00	0.00	0.00	0.00	0.00	*0.00*
Nd	0.00	0.00	0.01	0.00	0.01	0.00	0.01	0.00	0.00	0.01	0.00	0.00	0.01	0.00	*0.00*
Sm	0.00	0.00	0.00	0.00	0.00	0.00	0.00	0.00	0.00	0.00	0.00	0.00	0.00	0.00	*0.00*
Gd	0.00	0.00	0.00	0.00	0.00	0.00	0.00	0.00	0.00	0.00	0.00	0.00	0.00	0.00	*0.00*
Dy	0.00	0.00	0.00	0.00	0.00	0.00	0.00	0.00	0.00	0.00	0.00	0.00	0.00	0.00	*0.00*
Ca	5.05	5.05	5.04	5.00	5.02	5.00	5.00	5.03	5.00	4.99	5.02	4.97	4.95	4.97	*5.01*
Fe	0.01	0.01	0.03	0.01	0.01	0.05	0.01	0.01	0.02	0.05	0.01	0.01	0.05	0.01	*0.02*
Mn	0.00	0.00	0.00	0.00	0.00	0.00	0.00	0.00	0.00	0.00	0.00	0.00	0.00	0.00	*0.00*
Sr	0.00	0.00	0.00	0.00	0.00	0.00	0.00	0.00	0.00	0.00	0.00	0.00	0.00	0.00	*0.00*
Na	0.00	0.00	0.00	0.00	0.00	0.00	0.00	0.00	0.00	0.00	0.00	0.00	0.00	0.00	*0.00*
F	0.94	0.97	1.01	0.96	0.95	0.91	0.99	1.01	0.98	0.95	0.94	0.96	0.99	0.99	*0.97*
Cl	0.01	0.01	0.01	0.00	0.00	0.00	0.00	0.00	0.00	0.00	0.00	0.00	0.00	0.00	*0.00*
OH	0.06	0.06	0.06	0.06	0.06	0.06	0.06	0.06	0.06	0.06	0.06	0.06	0.06	0.06	*0.06*
Total	1.01	1.04	1.07	1.02	1.01	0.97	1.05	1.07	1.04	1.01	1.00	1.02	1.04	1.05	*1.03*
XF/XOH	16.22	16.79	17.40	16.73	16.63	16.00	17.27	17.67	17.35	16.76	16.53	16.92	17.42	17.68	*16.95*
XCl/XOH	0.12	0.11	0.11	0.06	0.06	0.06	0.06	0.05	0.04	0.04	0.04	0.04	0.03	0.02	*0.06*

Page 18																
Charnockite sample	16															*16*
No. on map	37															*37*
Sample No.	NIL2-8															NIL2-8
No of grains analysed	1	2	3	4	5	6	7	8	9	10	11	12	13	14	15	16	16
																Mean
P_2O_5	42.03	42.11	42.38	42.34	42.09	41.93	42.44	42.07	43.29	40.09	42.39	42.24	42.19	42.24	42.55	42.02	*42.15*
SiO_2	0.15	0.16	0.00	0.00	0.13	0.13	0.14	0.18	0.00	0.10	0.17	0.11	0.17	0.19	0.17	0.14	*0.12*
Al_2O_3	0.00	0.00	0.00	0.00	0.00	0.00	0.00	0.00	0.00	0.00	0.00	0.00	0.00	0.00	0.00	0.00	*0.00*
SO_2	0.00	0.00	0.00	0.00	0.01	0.01	0.04	0.00	0.02	0.00	0.00	0.00	0.00	0.00	0.00	0.01	*0.01*
Y_2O_3	0.01	0.02	0.00	0.00	0.00	0.00	0.03	0.00	0.00	0.00	0.02	0.09	0.01	0.06	0.00	0.00	*0.01*
La_2O_3	0.00	0.00	0.00	0.01	0.00	0.00	0.00	0.03	0.00	0.01	0.04	0.02	0.00	0.00	0.03	0.02	*0.01*
Ce_2O_3	0.12	0.03	0.08	0.07	0.05	0.02	0.07	0.01	0.04	0.09	0.04	0.02	0.06	0.10	0.00	0.04	*0.05*
Pr_2O_3	0.00	0.04	0.13	0.01	0.00	0.01	0.00	0.00	0.01	0.02	0.00	0.06	0.02	0.02	0.01	0.03	*0.02*
Nd_2O_3	0.12	0.06	0.12	0.08	0.07	0.09	0.02	0.05	0.12	0.08	0.08	0.10	0.06	0.08	0.02	0.16	*0.08*
Sm_2O_3	0.05	0.01	0.08	0.01	0.00	0.01	0.08	0.06	0.04	0.03	0.03	0.00	0.03	0.02	0.04	0.05	*0.03*
Gd_2O_3	0.00	0.01	0.07	0.00	0.00	0.00	0.05	0.00	0.09	0.09	0.00	0.07	0.00	0.07	0.00	0.11	*0.03*
Dy_2O_3	0.00	0.00	0.02	0.03	0.00	0.00	0.02	0.05	0.00	0.01	0.00	0.00	0.00	0.00	0.05	0.01	*0.01*
CaO	55.46	55.31	57.22	57.35	56.16	55.69	55.95	55.43	57.77	53.54	55.52	55.86	55.94	55.79	55.98	55.50	*55.90*
FeO	0.13	0.13	0.60	0.09	0.08	0.27	0.42	0.44	0.11	0.14	0.05	0.26	0.27	0.10	0.29	0.64	*0.25*
MnO	0.00	0.02	0.00	0.00	0.00	0.00	0.00	0.00	0.00	0.02	0.00	0.00	0.04	0.00	0.01	0.00	*0.01*
SrO	0.00	0.04	0.00	0.00	0.00	0.00	0.01	0.05	0.00	0.08	0.00	0.04	0.05	0.02	0.03	0.04	*0.02*
Na_2O	0.00	0.00	0.00	0.00	0.00	0.00	0.00	0.00	0.02	0.04	0.00	0.00	0.00	0.00	0.00	0.00	*0.01*
F	3.78	3.84	3.96	4.20	3.73	3.73	3.76	3.66	6.01	3.62	3.73	3.90	3.90	3.72	3.42	3.76	*3.92*
Cl	0.01	0.01	0.01	0.01	0.01	0.01	0.01	0.01	0.01	0.00	0.00	0.00	0.00	0.00	0.00	0.00	*0.00*
H_2O	0.10	0.10	0.10	0.10	0.10	0.10	0.10	0.10	0.10	0.10	0.10	0.10	0.10	0.10	0.10	0.10	*0.10*
(F+Cl+OH)	0.00	0.00	0.00	0.00	0.00	0.00	0.00	0.00	0.00	0.00	0.00	0.00	0.00	0.00	0.00	0.00	*0.00*
sum	101.95	101.89	104.76	104.29	102.43	101.98	103.13	102.14	107.62	98.06	102.18	102.88	102.84	102.49	102.69	102.62	*102.75*
O=(F+Cl)	1.59	1.62	1.67	1.77	1.57	1.57	1.58	1.54	2.53	1.53	1.57	1.64	1.64	1.57	1.44	1.58	*1.65*
total	100.35	100.27	103.09	102.52	100.86	100.41	101.55	100.60	105.09	96.53	100.60	101.23	101.19	100.93	101.25	101.04	*101.09*
(Y+REE)	0.30	0.18	0.49	0.21	0.13	0.13	0.26	0.20	0.30	0.33	0.21	0.36	0.18	0.33	0.15	0.41	*0.26*
P	2.98	2.98	2.95	2.95	2.97	2.97	2.98	2.98	2.91	2.96	2.99	2.97	2.97	2.98	2.99	2.97	*2.97*
Si	0.01	0.01	0.00	0.00	0.01	0.01	0.01	0.02	0.00	0.01	0.01	0.01	0.01	0.02	0.01	0.01	*0.01*
Al	0.00	0.00	0.00	0.00	0.00	0.00	0.00	0.00	0.00	0.00	0.00	0.00	0.00	0.00	0.00	0.00	*0.00*
S	0.00	0.00	0.00	0.00	0.00	0.00	0.01	0.00	0.00	0.00	0.00	0.00	0.00	0.00	0.00	0.00	*0.00*
Y	0.00	0.00	0.00	0.00	0.00	0.00	0.00	0.00	0.00	0.00	0.00	0.00	0.00	0.00	0.00	0.00	*0.00*
La	0.00	0.00	0.00	0.00	0.00	0.00	0.00	0.00	0.00	0.00	0.00	0.00	0.00	0.00	0.00	0.00	*0.00*
Ce	0.00	0.00	0.00	0.00	0.00	0.00	0.00	0.00	0.00	0.00	0.00	0.00	0.00	0.00	0.00	0.00	*0.00*
Pr	0.00	0.00	0.00	0.00	0.00	0.00	0.00	0.00	0.00	0.00	0.00	0.00	0.00	0.00	0.00	0.00	*0.00*
Nd	0.00	0.00	0.00	0.00	0.00	0.00	0.00	0.00	0.00	0.00	0.00	0.00	0.00	0.00	0.00	0.00	*0.00*
Sm	0.00	0.00	0.00	0.00	0.00	0.00	0.00	0.00	0.00	0.00	0.00	0.00	0.00	0.00	0.00	0.00	*0.00*
Gd	0.00	0.00	0.00	0.00	0.00	0.00	0.00	0.00	0.00	0.00	0.00	0.00	0.00	0.00	0.00	0.00	*0.00*
Dy	0.00	0.00	0.00	0.00	0.00	0.00	0.00	0.00	0.00	0.00	0.00	0.00	0.00	0.00	0.00	0.00	*0.00*
Ca	4.97	4.96	5.03	5.05	5.02	5.00	4.97	4.97	4.92	5.01	4.96	4.97	4.98	4.98	4.98	4.96	*4.98*
Fe	0.02	0.02	0.08	0.01	0.01	0.04	0.06	0.06	0.02	0.02	0.01	0.04	0.04	0.01	0.04	0.09	*0.03*
Mn	0.00	0.00	0.00	0.00	0.00	0.00	0.00	0.00	0.00	0.00	0.00	0.00	0.00	0.00	0.00	0.00	*0.00*
Sr	0.00	0.00	0.00	0.00	0.00	0.00	0.00	0.00	0.00	0.00	0.00	0.00	0.00	0.00	0.00	0.00	*0.00*
Na	0.00	0.00	0.00	0.00	0.00	0.00	0.00	0.00	0.00	0.01	0.00	0.00	0.00	0.00	0.00	0.00	*0.00*
F	1.00	1.01	1.03	1.09	0.98	0.99	0.98	0.97	1.51	1.00	0.98	1.02	1.02	0.98	0.90	0.99	*1.03*
Cl	0.00	0.00	0.00	0.00	0.00	0.00	0.00	0.00	0.00	0.00	0.00	0.00	0.00	0.00	0.00	0.00	*0.00*
OH	0.06	0.06	0.05	0.05	0.06	0.06	0.06	0.06	0.05	0.06	0.06	0.06	0.06	0.06	0.06	0.06	*0.06*
Total	1.06	1.07	1.08	1.15	1.04	1.04	1.04	1.03	1.56	1.06	1.04	1.08	1.08	1.04	0.95	1.05	*1.09*
XF/XOH	17.92	18.18	18.77	19.89	17.67	17.67	17.81	17.37	28.51	17.16	17.70	18.48	18.49	17.63	16.21	17.82	*18.58*
XCl/XOH	0.02	0.02	0.02	0.02	0.02	0.02	0.01	0.01	0.01	0.01	0.01	0.01	0.01	0.00	0.00	0.00	*0.01*

Page 19																	
Charnockite sample	17																
No. on map	45																continued on next page
Sample No.	NIL2-21																NIL2-21
No of grains analysed	1	2	3	4	5	6	7	8	9	10	11	12	13	14	15	16	17
P_2O_5	41.50	40.92	40.64	40.44	40.82	40.88	40.61	40.78	40.28	41.07	40.81	41.80	41.07	40.45	40.33	40.42	41.39
SiO_2	0.00	0.03	0.16	0.13	0.15	0.15	0.15	0.17	0.15	0.17	0.17	0.00	0.18	0.12	0.17	0.13	0.14
Al_2O_3	0.00	0.00	0.00	0.00	0.00	0.00	0.00	0.00	0.00	0.00	0.00	0.00	0.00	0.00	0.00	0.00	0.00
SO_2	0.02	0.02	0.01	0.04	0.01	0.02	0.00	0.00	0.02	0.01	0.00	0.01	0.00	0.00	0.01	0.01	0.01
Y_2O_3	0.00	0.00	0.00	0.00	0.02	0.00	0.00	0.00	0.01	0.00	0.02	0.00	0.03	0.00	0.00	0.04	0.00
La_2O_3	0.00	0.00	0.00	0.01	0.09	0.03	0.00	0.03	0.04	0.00	0.03	0.01	0.00	0.00	0.01	0.05	0.01
Ce_2O_3	0.05	0.02	0.05	0.07	0.11	0.06	0.00	0.03	0.01	0.09	0.00	0.05	0.01	0.00	0.01	0.10	0.00
Pr_2O_3	0.20	0.00	0.00	0.05	0.00	0.00	0.02	0.00	0.00	0.00	0.00	0.20	0.00	0.01	0.02	0.05	0.09
Nd_2O_3	0.11	0.01	0.11	0.15	0.11	0.06	0.08	0.06	0.05	0.11	0.07	0.13	0.08	0.11	0.07	0.16	0.07
Sm_2O_3	0.06	0.00	0.00	0.00	0.00	0.01	0.02	0.06	0.07	0.03	0.00	0.01	0.04	0.03	0.04	0.00	0.00
Gd_2O_3	0.05	0.00	0.00	0.00	0.01	0.06	0.06	0.01	0.05	0.00	0.00	0.06	0.04	0.00	0.00	0.02	0.00
Dy_2O_3	0.08	0.00	0.03	0.00	0.00	0.00	0.00	0.00	0.00	0.00	0.00	0.00	0.00	0.00	0.02	0.00	0.01
CaO	55.86	55.04	54.12	54.66	53.85	53.81	53.55	54.34	53.40	53.90	54.21	56.61	54.42	53.40	53.84	53.32	54.44
FeO	0.83	0.05	0.09	0.08	0.48	0.03	0.14	0.07	0.06	0.07	0.05	0.13	0.11	0.14	0.07	0.06	0.08
MnO	0.00	0.03	0.01	0.00	0.00	0.00	0.01	0.02	0.02	0.02	0.00	0.00	0.00	0.02	0.02	0.01	0.01
SrO	0.00	0.01	0.00	0.00	0.05	0.03	0.00	0.01	0.07	0.00	0.01	0.00	0.00	0.01	0.03	0.00	0.01
Na_2O	0.00	0.00	0.01	0.00	0.01	0.00	0.01	0.01	0.01	0.01	0.01	0.00	0.00	0.01	0.00	0.01	0.01
F	4.54	3.35	3.48	2.99	3.47	3.62	3.62	3.74	3.31	3.64	3.47	4.13	3.52	3.60	3.39	3.42	3.64
Cl	0.30	0.22	0.19	0.18	0.18	0.16	0.12	0.11	0.10	0.08	0.06	0.05	0.04	0.04	0.03	0.03	0.03
H_2O		0.15	0.10	0.30	0.10	0.10	0.10	0.10	0.10	0.10	0.10	0.10	0.10	0.10	0.10	0.10	0.10
(F+Cl+OH)	0.00	0.00	0.00	0.00	0.00	0.00	0.00	0.00	0.00	0.00	0.00	0.00	0.00	0.00	0.00	0.00	0.00
sum	103.59	99.85	99.01	99.09	99.44	99.00	98.48	99.54	97.73	99.30	98.98	103.28	99.64	98.05	98.15	97.92	100.05
O=(F+Cl)	1.98	1.46	1.51	1.30	1.50	1.56	1.55	1.60	1.42	1.55	1.47	1.75	1.49	1.53	1.43	1.45	1.54
total	101.61	98.39	97.50	97.79	97.94	97.44	96.93	97.95	96.32	97.75	97.51	101.53	98.15	96.53	96.72	96.47	98.51
(Y+REE)	0.54	0.04	0.19	0.28	0.34	0.21	0.18	0.20	0.22	0.24	0.12	0.46	0.21	0.16	0.17	0.42	0.18
P	2.93	2.97	2.97	2.96	2.97	2.98	2.98	2.96	2.98	2.99	2.98	2.94	2.98	2.98	2.97	2.99	2.99
Si	0.00	0.00	0.01	0.01	0.01	0.01	0.01	0.01	0.01	0.01	0.01	0.00	0.02	0.01	0.01	0.01	0.01
Al	0.00	0.00	0.00	0.00	0.00	0.00	0.00	0.00	0.00	0.00	0.00	0.00	0.00	0.00	0.00	0.00	0.00
S	0.00	0.00	0.00	0.00	0.00	0.00	0.00	0.00	0.00	0.00	0.00	0.00	0.00	0.00	0.00	0.00	0.00
Y	0.00	0.00	0.00	0.00	0.00	0.00	0.00	0.00	0.00	0.00	0.00	0.00	0.00	0.00	0.00	0.00	0.00
La	0.00	0.00	0.00	0.00	0.00	0.00	0.00	0.00	0.00	0.00	0.00	0.00	0.00	0.00	0.00	0.00	0.00
Ce	0.00	0.00	0.00	0.00	0.00	0.00	0.00	0.00	0.00	0.00	0.00	0.00	0.00	0.00	0.00	0.00	0.00
Pr	0.00	0.00	0.00	0.00	0.00	0.00	0.00	0.00	0.00	0.00	0.00	0.00	0.00	0.00	0.00	0.00	0.00
Nd	0.00	0.00	0.00	0.00	0.00	0.00	0.00	0.00	0.00	0.00	0.00	0.00	0.00	0.00	0.00	0.01	0.00
Sm	0.00	0.00	0.00	0.00	0.00	0.00	0.00	0.00	0.00	0.00	0.00	0.00	0.00	0.00	0.00	0.00	0.00
Gd	0.00	0.00	0.00	0.00	0.00	0.00	0.00	0.00	0.00	0.00	0.00	0.00	0.00	0.00	0.00	0.00	0.00
Dy	0.00	0.00	0.00	0.00	0.00	0.00	0.00	0.00	0.00	0.00	0.00	0.00	0.00	0.00	0.00	0.00	0.00
Ca	4.98	5.05	5.01	5.06	4.97	4.97	4.97	5.00	5.00	4.96	5.01	5.04	5.00	4.98	5.02	4.98	4.97
Fe	0.12	0.01	0.01	0.01	0.07	0.00	0.02	0.01	0.01	0.01	0.01	0.02	0.02	0.02	0.01	0.01	0.01
Mn	0.00	0.00	0.00	0.00	0.00	0.00	0.00	0.00	0.00	0.00	0.00	0.00	0.00	0.00	0.00	0.00	0.00
Sr	0.00	0.00	0.00	0.00	0.00	0.00	0.00	0.00	0.00	0.00	0.00	0.00	0.00	0.00	0.00	0.00	0.00
Na	0.00	0.00	0.00	0.00	0.00	0.00	0.00	0.00	0.00	0.00	0.00	0.00	0.00	0.00	0.00	0.00	0.00
F	1.20	0.91	0.95	0.82	0.95	0.99	0.99	1.01	0.92	0.99	0.95	1.09	0.95	0.99	0.93	0.94	0.98
Cl	0.04	0.03	0.03	0.03	0.03	0.02	0.02	0.02	0.02	0.01	0.01	0.01	0.01	0.01	0.00	0.00	0.00
OH	0.00	0.09	0.06	0.17	0.06	0.06	0.06	0.06	0.06	0.06	0.06	0.06	0.06	0.06	0.06	0.06	0.06
Total	1.24	1.02	1.04	1.01	1.03	1.07	1.07	1.09	0.99	1.06	1.01	1.15	1.01	1.05	1.00	1.01	1.04
XF/XOH		10.59	16.50	4.72	16.47	17.15	17.14	17.71	15.70	17.24	16.44	19.60	16.66	17.08	16.07	16.19	17.27
XCl/XOH		0.37	0.48	0.15	0.44	0.40	0.30	0.28	0.26	0.20	0.15	0.11	0.09	0.09	0.08	0.08	0.08

Page 20																			
Chamockite sample	17 cont'd																		17
No. on map	45																		45
Sample No.	NIL2-21																		NIL2-21
No of grains analysed	18	19	20	21	22	23	24	25	26	27	28	29	30	31	32	33	34	35	35
																			Mean
P_2O_5	40.87	40.97	41.46	41.15	41.27	41.08	40.21	41.10	41.59	40.97	40.79	40.93	41.08	42.10	40.86	41.36	40.85	40.99	40.97
SiO_2	0.18	0.19	0.16	0.18	0.13	0.13	0.11	0.14	0.16	0.15	0.03	0.10	0.02	0.00	0.04	0.01	0.06	0.09	0.11
Al_2O_3	0.00	0.00	0.00	0.00	0.00	0.00	0.00	0.00	0.00	0.00	0.00	0.00	0.00	0.00	0.00	0.00	0.00	0.00	0.00
SO_2	0.02	0.01	0.00	0.02	0.01	0.00	0.00	0.00	0.01	0.02	0.01	0.01	0.00	0.02	0.00	0.00	0.01	0.00	0.01
Y_2O_3	0.00	0.00	0.00	0.00	0.00	0.00	0.01	0.00	0.01	0.00	0.00	0.00	0.00	0.00	0.00	0.00	0.00	0.00	0.00
La_2O_3	0.00	0.00	0.01	0.02	0.00	0.00	0.08	0.01	0.01	0.00	0.00	0.00	0.01	0.00	0.08	0.04	0.01	0.04	0.02
Ce_2O_3	0.07	0.02	0.05	0.06	0.11	0.05	0.06	0.04	0.05	0.06	0.04	0.04	0.01	0.07	0.00	0.00	0.01	0.10	0.04
Pr_2O_3	0.02	0.01	0.00	0.00	0.07	0.00	0.02	0.00	0.00	0.01	0.00	0.00	0.08	0.00	0.00	0.00	0.06	0.04	0.03
Nd_2O_3	0.13	0.02	0.09	0.07	0.14	0.06	0.14	0.05	0.12	0.06	0.12	0.03	0.08	0.12	0.03	0.07	0.08	0.11	0.09
Sm_2O_3	0.07	0.03	0.03	0.00	0.11	0.01	0.00	0.00	0.00	0.03	0.00	0.00	0.00	0.12	0.00	0.00	0.00	0.00	0.02
Gd_2O_3	0.00	0.04	0.01	0.06	0.12	0.02	0.00	0.00	0.09	0.04	0.00	0.00	0.00	0.08	0.00	0.00	0.00	0.00	0.02
Dy_2O_3	0.00	0.00	0.00	0.04	0.00	0.07	0.00	0.01	0.00	0.00	0.00	0.00	0.00	0.00	0.00	0.00	0.00	0.00	0.01
CaO	53.47	54.15	54.49	54.65	54.10	53.94	55.01	54.35	54.33	54.10	55.46	54.99	55.40	56.31	55.27	55.08	54.96	55.14	54.51
FeO	0.48	0.12	0.04	0.04	0.06	0.04	0.08	0.12	0.07	0.06	0.09	0.08	0.04	0.40	0.06	0.05	0.34	0.12	0.14
MnO	0.03	0.00	0.00	0.00	0.00	0.01	0.09	0.00	0.00	0.01	0.00	0.02	0.00	0.00	0.10	0.03	0.00	0.00	0.01
SrO	0.02	0.00	0.00	0.01	0.07	0.00	0.00	0.05	0.00	0.00	0.00	0.00	0.00	0.00	0.00	0.00	0.00	0.00	0.01
Na_2O	0.01	0.01	0.00	0.00	0.00	0.00	0.00	0.00	0.01	0.01	0.00	0.00	0.00	0.00	0.00	0.00	0.00	0.00	0.00
F	3.60	3.64	3.66	3.62	3.42	3.69	3.15	3.69	3.51	3.80	3.56	3.65	3.57	4.51	3.56	3.14	3.42	2.98	3.57
Cl	0.03	0.03	0.03	0.03	0.03	0.03	0.03	0.02	0.02	0.02	0.02	0.01	0.01	0.01	0.00	0.00	0.00	0.00	0.06
H_2O	0.10	0.10	0.10	0.10	0.10	0.10	0.30	0.10	0.10	0.10	0.10	0.10	0.10	0.10	0.10	0.30	0.30	0.40	0.04
(F+Cl+OH)	0.00	0.00	0.00	0.00	0.00	0.00	0.00	0.00	0.00	0.00	0.00	0.00	0.00	0.00	0.00	0.00	0.00	0.00	0.00
sum	99.10	99.32	100.13	100.05	99.72	99.22	99.29	99.67	100.07	99.41	100.22	99.96	100.41	103.83	100.11	100.09	100.10	100.02	99.77
O=(F+Cl)	1.52	1.54	1.55	1.53	1.45	1.56	1.33	1.56	1.48	1.60	1.50	1.54	1.51	1.90	1.50	1.32	1.44	1.25	1.52
total	97.58	97.78	98.58	98.52	98.27	97.66	97.96	98.11	98.59	97.81	98.72	98.42	98.90	101.93	98.61	98.77	98.66	98.76	98.25
(Y+REE)	0.29	0.12	0.19	0.26	0.53	0.20	0.30	0.11	0.27	0.19	0.16	0.07	0.18	0.39	0.11	0.11	0.16	0.30	0.23
P	2.98	2.98	2.99	2.97	2.99	2.99	2.94	2.98	3.00	2.98	2.95	2.96	2.97	2.94	2.96	2.98	2.95	2.96	2.98
Si	0.02	0.02	0.01	0.02	0.01	0.01	0.01	0.01	0.01	0.01	0.00	0.01	0.00	0.00	0.00	0.00	0.00	0.01	0.01
Al	0.00	0.00	0.00	0.00	0.00	0.00	0.00	0.00	0.00	0.00	0.00	0.00	0.00	0.00	0.00	0.00	0.00	0.00	0.00
S	0.00	0.00	0.00	0.00	0.00	0.00	0.00	0.00	0.00	0.00	0.00	0.00	0.00	0.00	0.00	0.00	0.00	0.00	0.00
Y	0.00	0.00	0.00	0.00	0.00	0.00	0.00	0.00	0.00	0.00	0.00	0.00	0.00	0.00	0.00	0.00	0.00	0.00	0.00
La	0.00	0.00	0.00	0.00	0.00	0.00	0.00	0.00	0.00	0.00	0.00	0.00	0.00	0.00	0.00	0.00	0.00	0.00	0.00
Ce	0.00	0.00	0.00	0.00	0.00	0.00	0.00	0.00	0.00	0.00	0.00	0.00	0.00	0.00	0.00	0.00	0.00	0.00	0.00
Pr	0.00	0.00	0.00	0.00	0.00	0.00	0.00	0.00	0.00	0.00	0.00	0.00	0.00	0.00	0.00	0.00	0.00	0.00	0.00
Nd	0.00	0.00	0.00	0.00	0.00	0.00	0.00	0.00	0.00	0.00	0.00	0.00	0.00	0.00	0.00	0.00	0.00	0.00	0.00
Sm	0.00	0.00	0.00	0.00	0.00	0.00	0.00	0.00	0.00	0.00	0.00	0.00	0.00	0.00	0.00	0.00	0.00	0.00	0.00
Gd	0.00	0.00	0.00	0.00	0.00	0.00	0.00	0.00	0.00	0.00	0.00	0.00	0.00	0.00	0.00	0.00	0.00	0.00	0.00
Dy	0.00	0.00	0.00	0.00	0.00	0.00	0.00	0.00	0.00	0.00	0.00	0.00	0.00	0.00	0.00	0.00	0.00	0.00	0.00
Ca	4.94	4.98	4.97	5.00	4.97	4.96	5.09	4.99	4.96	4.97	5.08	5.04	5.06	4.98	5.07	5.02	5.03	5.04	5.01
Fe	0.07	0.02	0.01	0.01	0.01	0.01	0.01	0.02	0.01	0.01	0.01	0.01	0.01	0.05	0.01	0.01	0.05	0.02	0.02
Mn	0.00	0.00	0.00	0.00	0.00	0.00	0.01	0.00	0.00	0.00	0.00	0.00	0.00	0.00	0.01	0.01	0.00	0.00	0.00
Sr	0.00	0.00	0.00	0.00	0.00	0.00	0.00	0.00	0.00	0.00	0.00	0.00	0.00	0.00	0.00	0.00	0.00	0.00	0.00
Na	0.00	0.00	0.00	0.00	0.00	0.00	0.00	0.00	0.00	0.00	0.00	0.00	0.00	0.00	0.00	0.00	0.00	0.00	0.00
F	0.98	0.99	0.98	0.98	0.93	1.00	0.86	1.00	0.95	1.03	0.96	0.99	0.96	1.18	0.96	0.85	0.92	0.80	0.97
Cl	0.00	0.00	0.00	0.00	0.00	0.00	0.00	0.00	0.00	0.00	0.00	0.00	0.00	0.00	0.00	0.00	0.00	0.00	0.01
OH	0.06	0.06	0.06	0.06	0.06	0.06	0.17	0.06	0.06	0.06	0.06	0.06	0.06	0.06	0.06	0.17	0.17	0.23	0.02
Total	1.04	1.05	1.05	1.04	0.99	1.06	1.04	1.06	1.01	1.09	1.02	1.05	1.02	1.23	1.02	1.02	1.09	1.03	1.00
XF/XOH	17.07	17.24	17.34	17.14	16.20	17.48		17.48	16.64	17.99	16.88	17.30	16.92	21.40	16.88				17.43
XCl/XOH	0.08	0.07	0.07	0.07	0.07	0.07	0.02	0.06	0.06	0.05	0.05	0.03	0.03	0.02	0.01	0.00	0.00	0.00	0.04

Page 21											
Charnockite sample	18										*18*
No. on map	61										*61*
Sample No.	NIL26-24										NIL26-24
No of grains analysed	1	2	3	4	5	6	7	8	9	10	10
											Mean
P_2O_5	40.18	40.52	40.85	40.88	41.51	41.61	40.91	39.59	41.33	41.81	*40.92*
SiO_2	0.13	0.17	0.13	0.14	0.15	0.16	0.17	0.06	0.15	0.14	*0.14*
Al_2O_3	0.00	0.00	0.00	0.00	0.00	0.00	0.00	0.00	0.00	0.00	*0.00*
SO_2	0.01	0.03	0.02	0.02	0.01	0.01	0.01	0.00	0.01	0.00	*0.01*
Y_2O_3	0.02	0.02	0.07	0.04	0.00	0.00	0.01	0.05	0.00	0.00	*0.02*
La_2O_3	0.48	0.03	0.00	0.00	0.01	0.02	0.02	0.00	0.00	0.00	*0.06*
Ce_2O_3	1.40	0.29	0.04	0.04	0.09	0.05	0.03	0.05	0.09	0.03	*0.21*
Pr_2O_3	0.21	0.06	0.03	0.00	0.00	0.03	0.00	0.03	0.01	0.01	*0.04*
Nd_2O_3	0.87	0.25	0.08	0.09	0.10	0.04	0.03	0.03	0.12	0.03	*0.16*
Sm_2O_3	0.14	0.07	0.07	0.09	0.00	0.06	0.00	0.02	0.05	0.00	*0.05*
Gd_2O_3	0.28	0.00	0.08	0.04	0.08	0.03	0.06	0.03	0.03	0.00	*0.06*
Dy_2O_3	0.01	0.03	0.05	0.02	0.03	0.00	0.03	0.00	0.01	0.02	*0.02*
CaO	52.04	53.31	55.17	54.40	55.00	54.99	55.23	52.52	54.74	54.80	*54.22*
FeO	0.08	0.82	0.16	0.08	0.53	0.58	0.35	0.29	0.35	0.12	*0.33*
MnO	0.00	0.01	0.00	0.01	0.00	0.03	0.01	0.01	0.01	0.02	*0.01*
SrO	0.00	0.01	0.00	0.01	0.03	0.02	0.00	0.03	0.00	0.04	*0.01*
Na_2O	0.01	0.03	0.01	0.01	0.01	0.00	0.00	0.01	0.01	0.00	*0.01*
F	3.28	3.58	3.47	3.64	3.67	3.79	3.68	3.61	3.70	3.72	*3.62*
Cl	0.06	0.05	0.04	0.04	0.03	0.02	0.02	0.02	0.02	0.02	*0.03*
H_2O	0.10	0.10	0.10	0.10	0.10	0.10	0.10	0.10	0.10	0.10	*0.02*
(F+Cl+OH)	0.00	0.00	0.00	0.00	0.00	0.00	0.00	0.00	0.00	0.00	*0.00*
sum	99.30	99.36	100.36	99.62	101.34	101.53	100.66	96.45	100.72	100.84	*100.02*
O=(F+Cl)	1.40	1.52	1.47	1.54	1.55	1.60	1.55	1.53	1.56	1.57	*1.53*
total	97.91	97.84	98.89	98.08	99.79	99.93	99.11	94.92	99.15	99.27	*98.49*
(Y+REE)	3.41	0.74	0.41	0.31	0.30	0.24	0.18	0.21	0.30	0.08	*0.62*
P	2.98	2.96	2.96	2.97	2.97	2.97	2.95	2.97	2.97	2.99	*2.97*
Si	0.01	0.01	0.01	0.01	0.01	0.01	0.01	0.01	0.01	0.01	*0.01*
Al	0.00	0.00	0.00	0.00	0.00	0.00	0.00	0.00	0.00	0.00	*0.00*
S	0.00	0.00	0.00	0.00	0.00	0.00	0.00	0.00	0.00	0.00	*0.00*
Y	0.00	0.00	0.00	0.00	0.00	0.00	0.00	0.00	0.00	0.00	*0.00*
La	0.02	0.00	0.00	0.00	0.00	0.00	0.00	0.00	0.00	0.00	*0.00*
Ce	0.04	0.01	0.00	0.00	0.00	0.00	0.00	0.00	0.00	0.00	*0.01*
Pr	0.00	0.00	0.00	0.00	0.00	0.00	0.00	0.00	0.00	0.00	*0.00*
Nd	0.03	0.01	0.00	0.00	0.00	0.00	0.00	0.00	0.00	0.00	*0.01*
Sm	0.00	0.00	0.00	0.00	0.00	0.00	0.00	0.00	0.00	0.00	*0.00*
Gd	0.01	0.00	0.00	0.00	0.00	0.00	0.00	0.00	0.00	0.00	*0.00*
Dy	0.00	0.00	0.00	0.00	0.00	0.00	0.00	0.00	0.00	0.00	*0.00*
Ca	4.88	4.94	5.05	5.00	4.98	4.96	5.04	4.99	4.98	4.96	*4.98*
Fe	0.01	0.12	0.02	0.01	0.07	0.08	0.05	0.04	0.05	0.02	*0.05*
Mn	0.00	0.00	0.00	0.00	0.00	0.00	0.00	0.00	0.00	0.00	*0.00*
Sr	0.00	0.00	0.00	0.00	0.00	0.00	0.00	0.00	0.00	0.00	*0.00*
Na	0.00	0.00	0.00	0.00	0.00	0.00	0.00	0.00	0.00	0.00	*0.00*
F	0.91	0.98	0.94	0.99	0.98	1.01	0.99	1.01	0.99	0.99	*0.98*
Cl	0.01	0.01	0.01	0.01	0.00	0.00	0.00	0.00	0.00	0.00	*0.00*
OH	0.06	0.06	0.06	0.06	0.06	0.06	0.06	0.06	0.06	0.06	*0.06*
Total	0.98	1.04	1.00	1.05	1.04	1.07	1.05	1.08	1.05	1.05	*1.04*
XF/XOH	15.57	16.96	16.43	17.24	17.42	17.99	17.45	17.13	17.56	17.65	*17.14*
XCl/XOH	0.14	0.12	0.10	0.09	0.06	0.06	0.06	0.05	0.05	0.04	*0.08*

Page 22																			
Charnockite sample	19																		*19*
No. on map	40																		*40*
Sample No.	NIL2-11																		NIL2-11
No of grains analysed	1	2	3	4	5	6	7	8	9	10	11	12	13	14	15	16	17	18	18
																			Mean
P_2O_5	40.88	40.52	41.55	40.83	40.01	41.18	40.93	41.05	40.21	40.40	39.87	41.19	40.74	39.11	39.92	40.08	40.37	39.69	*40.47*
SiO_2	0.58	0.52	0.57	0.53	0.00	0.57	0.55	0.53	0.00	0.53	0.00	0.55	0.53	0.00	0.00	0.00	0.00	0.49	*0.33*
Al_2O_3	0.00	0.00	0.00	0.00	0.00	0.00	0.00	0.00	0.00	0.00	0.00	0.00	0.00	0.00	0.00	0.00	0.00	0.00	*0.00*
SO_2	0.02	0.01	0.00	0.01	0.03	0.00	0.00	0.00	0.02	0.01	0.00	0.00	0.02	0.00	0.09	0.00	0.00	0.00	*0.01*
Y_2O_3	0.15	0.10	0.14	0.08	0.08	0.09	0.11	0.19	0.08	0.11	0.10	0.08	0.12	0.05	0.06	0.05	0.08	0.15	*0.10*
La_2O_3	0.01	0.00	0.00	0.00	0.04	0.02	0.07	0.02	0.00	0.03	0.00	0.00	0.00	0.00	0.00	0.00	0.00	0.01	*0.01*
Ce_2O_3	0.04	0.01	0.07	0.09	0.20	0.01	0.14	0.11	0.04	0.03	0.05	0.09	0.01	0.13	0.03	0.08	0.06	0.00	*0.07*
Pr_2O_3	0.00	0.00	0.05	0.00	0.00	0.01	0.03	0.00	0.00	0.00	0.07	0.01	0.01	0.09	0.23	0.16	0.00	0.03	*0.04*
Nd_2O_3	0.06	0.01	0.11	0.02	0.26	0.03	0.10	0.10	0.01	0.08	0.06	0.07	0.01	0.09	0.08	0.13	0.07	0.07	*0.08*
Sm_2O_3	0.00	0.04	0.04	0.01	0.00	0.02	0.00	0.07	0.00	0.00	0.07	0.00	0.00	0.02	0.00	0.00	0.00	0.07	*0.02*
Gd_2O_3	0.07	0.00	0.03	0.00	0.00	0.08	0.10	0.05	0.01	0.00	0.00	0.01	0.00	0.00	0.08	0.01	0.00	0.08	*0.03*
Dy_2O_3	0.09	0.00	0.00	0.04	0.00	0.06	0.05	0.00	0.10	0.00	0.00	0.00	0.00	0.05	0.09	0.00	0.04	0.05	*0.03*
CaO	54.12	54.23	54.33	54.06	53.12	54.14	54.07	53.88	53.21	54.29	52.91	53.92	54.14	51.84	53.14	53.04	53.48	53.61	*53.64*
FeO	0.07	0.08	0.12	0.07	0.60	0.09	0.42	0.09	0.40	0.08	0.36	0.36	0.27	0.07	0.47	0.08	0.13	0.43	*0.23*
MnO	0.04	0.04	0.02	0.02	0.00	0.04	0.00	0.02	0.01	0.04	0.03	0.02	0.02	0.01	0.01	0.01	0.01	0.00	*0.02*
SrO	0.00	0.01	0.03	0.00	0.00	0.03	0.03	0.00	0.00	0.00	0.00	0.01	0.00	0.00	0.00	0.00	0.00	0.00	*0.01*
Na_2O	0.01	0.01	0.01	0.00	0.04	0.01	0.00	0.00	0.00	0.00	0.00	0.00	0.00	0.00	0.00	0.00	0.00	0.00	*0.00*
F	3.78	3.77	3.64	3.78	3.77	3.68	3.70	3.66	3.77	3.71	3.75	3.45	3.72	3.74	3.71	3.57	3.72	3.63	*3.70*
Cl	0.07	0.07	0.07	0.07	0.07	0.07	0.07	0.06	0.06	0.06	0.06	0.06	0.06	0.06	0.05	0.05	0.05	0.04	*0.06*
H_2O	0.00	0.00	0.10	0.00	0.00	0.00	0.00	0.00	0.00	0.00	0.00	0.10	0.00	0.00	0.00	0.00	0.00	0.00	*0.01*
(F+Cl+OH)	0.00	0.00	0.00	0.00	0.00	0.00	0.00	0.00	0.00	0.00	0.00	0.00	0.00	0.00	0.00	0.00	0.00	0.00	*0.00*
sum	99.98	99.43	100.87	99.62	98.19	100.11	100.35	99.83	97.91	99.37	97.33	99.92	99.65	95.27	97.97	97.26	98.02	98.37	*98.86*
O=(F+Cl)	1.61	1.60	1.55	1.61	1.60	1.56	1.57	1.55	1.60	1.58	1.59	1.46	1.58	1.59	1.58	1.52	1.58	1.54	*1.57*
total	98.37	97.82	99.32	98.01	96.59	98.55	98.78	98.28	96.31	97.79	95.74	98.45	98.07	93.68	96.39	95.74	96.44	96.83	*97.29*
(Y+REE)	0.42	0.15	0.44	0.23	0.57	0.32	0.58	0.54	0.24	0.25	0.35	0.27	0.16	0.43	0.57	0.42	0.25	0.46	*0.37*
P	2.96	2.95	2.97	2.96	2.96	2.97	2.96	2.97	2.98	2.95	2.97	2.98	2.96	2.98	2.96	2.99	2.98	2.93	*2.97*
Si	0.05	0.04	0.05	0.05	0.00	0.05	0.05	0.05	0.00	0.05	0.00	0.05	0.05	0.00	0.00	0.00	0.00	0.04	*0.03*
Al	0.00	0.00	0.00	0.00	0.00	0.00	0.00	0.00	0.00	0.00	0.00	0.00	0.00	0.00	0.00	0.00	0.00	0.00	*0.00*
S	0.00	0.00	0.00	0.00	0.00	0.00	0.00	0.00	0.00	0.00	0.00	0.00	0.00	0.00	0.01	0.00	0.00	0.00	*0.00*
Y	0.00	0.00	0.00	0.00	0.00	0.00	0.00	0.00	0.00	0.00	0.00	0.00	0.00	0.00	0.00	0.00	0.00	0.00	*0.00*
La	0.00	0.00	0.00	0.00	0.00	0.00	0.00	0.00	0.00	0.00	0.00	0.00	0.00	0.00	0.00	0.00	0.00	0.00	*0.00*
Ce	0.00	0.00	0.00	0.00	0.01	0.00	0.00	0.00	0.00	0.00	0.00	0.00	0.00	0.00	0.00	0.00	0.00	0.00	*0.00*
Pr	0.00	0.00	0.00	0.00	0.00	0.00	0.00	0.00	0.00	0.00	0.00	0.00	0.00	0.00	0.00	0.00	0.00	0.00	*0.00*
Nd	0.00	0.00	0.00	0.00	0.01	0.00	0.00	0.00	0.00	0.00	0.00	0.00	0.00	0.00	0.00	0.00	0.00	0.00	*0.00*
Sm	0.00	0.00	0.00	0.00	0.00	0.00	0.00	0.00	0.00	0.00	0.00	0.00	0.00	0.00	0.00	0.00	0.00	0.00	*0.00*
Gd	0.00	0.00	0.00	0.00	0.00	0.00	0.00	0.00	0.00	0.00	0.00	0.00	0.00	0.00	0.00	0.00	0.00	0.00	*0.00*
Dy	0.00	0.00	0.00	0.00	0.00	0.00	0.00	0.00	0.00	0.00	0.00	0.00	0.00	0.00	0.00	0.00	0.00	0.00	*0.00*
Ca	4.96	5.00	4.92	4.96	4.98	4.95	4.95	4.94	4.99	5.01	4.99	4.93	4.97	4.99	4.99	5.00	5.00	5.02	*4.97*
Fe	0.01	0.01	0.02	0.01	0.09	0.01	0.06	0.01	0.06	0.01	0.05	0.05	0.04	0.01	0.07	0.01	0.02	0.06	*0.03*
Mn	0.01	0.01	0.00	0.00	0.00	0.01	0.00	0.00	0.00	0.01	0.00	0.00	0.00	0.00	0.00	0.00	0.00	0.00	*0.00*
Sr	0.00	0.00	0.00	0.00	0.00	0.00	0.00	0.00	0.00	0.00	0.00	0.00	0.00	0.00	0.00	0.00	0.00	0.00	*0.00*
Na	0.00	0.00	0.00	0.00	0.01	0.00	0.00	0.00	0.00	0.00	0.00	0.00	0.00	0.00	0.00	0.00	0.00	0.00	*0.00*
F	1.02	1.03	0.97	1.03	1.04	0.99	1.00	0.99	1.04	1.01	1.05	0.93	1.01	1.06	1.03	0.99	1.03	1.00	*1.01*
Cl	0.01	0.01	0.01	0.01	0.01	0.01	0.01	0.01	0.01	0.01	0.01	0.01	0.01	0.01	0.01	0.01	0.01	0.01	*0.01*
OH	0.00	0.00	0.06	0.00	0.00	0.00	0.00	0.00	0.00	0.00	0.00	0.06	0.00	0.00	0.00	0.00	0.00	0.00	*0.01*
Total	1.03	1.04	1.04	1.03	1.05	1.00	1.01	1.00	1.05	1.02	1.05	1.00	1.02	1.07	1.04	1.00	1.04	1.01	*1.03*
XF/XOH			17.27									16.34							*16.81*
XCl/XOH			0.17									0.14							*0.16*

Page 23																		
Charnockite sample	20																	
No. on map	41																	continued on next page
Sample No.	NIL2-12																	NIL2-12
No of grains analysed	1	2	3	4	5	6	7	8	9	10	11	12	13	14	15	16	17	18
P_2O_5	40.80	39.55	39.97	40.15	39.15	38.75	39.16	40.42	40.25	39.66	39.10	39.99	40.82	40.55	40.33	40.15	38.91	38.90
SiO_2	0.13	0.16	0.17	0.14	0.16	0.00	0.12	0.16	0.18	0.12	0.12	0.18	0.12	0.15	0.16	0.13	0.00	0.13
Al_2O_3	0.00	0.00	0.00	0.00	0.00	0.00	0.00	0.00	0.00	0.00	0.00	0.00	0.00	0.00	0.00	0.00	0.00	0.00
SO_2	0.01	0.00	0.04	0.00	0.01	0.00	0.00	0.01	0.01	0.00	0.00	0.01	0.01	0.01	0.02	0.00	0.00	0.00
Y_2O_3	0.00	0.04	0.00	0.00	0.00	0.01	0.00	0.05	0.05	0.01	0.01	0.03	0.02	0.00	0.01	0.04	0.02	0.00
La_2O_3	0.04	0.04	0.00	0.00	0.02	0.07	0.00	0.00	0.00	0.00	0.04	0.00	0.00	0.00	0.00	0.00	0.03	0.00
Ce_2O_3	0.10	0.10	0.11	0.08	0.18	0.22	0.10	0.25	0.04	0.13	0.23	0.09	0.06	0.04	0.18	0.25	0.19	0.11
Pr_2O_3	0.08	0.00	0.03	0.06	0.02	0.00	0.00	0.02	0.03	0.05	0.05	0.01	0.04	0.05	0.04	0.07	0.00	0.02
Nd_2O_3	0.10	0.09	0.13	0.12	0.24	0.22	0.07	0.23	0.12	0.14	0.25	0.11	0.11	0.09	0.23	0.25	0.24	0.12
Sm_2O_3	0.00	0.00	0.00	0.02	0.09	0.00	0.00	0.00	0.02	0.06	0.00	0.00	0.00	0.06	0.00	0.05	0.00	0.06
Gd_2O_3	0.00	0.02	0.05	0.00	0.05	0.00	0.00	0.13	0.01	0.00	0.00	0.03	0.00	0.01	0.00	0.12	0.00	0.00
Dy_2O_3	0.00	0.04	0.00	0.00	0.05	0.00	0.00	0.02	0.03	0.03	0.02	0.01	0.00	0.02	0.04	0.00	0.05	0.00
CaO	54.97	51.70	53.01	52.96	50.62	51.07	51.53	52.48	53.23	52.67	50.58	52.75	52.31	53.85	52.40	52.53	51.24	51.22
FeO	0.53	0.05	0.24	0.45	0.80	0.49	1.40	0.79	0.44	0.46	0.59	0.42	0.52	0.28	0.70	0.48	0.34	0.66
MnO	0.06	0.02	0.00	0.00	0.05	0.00	0.01	0.01	0.03	0.03	0.00	0.01	0.00	0.00	0.00	0.02	0.00	0.01
SrO	0.00	0.01	0.00	0.02	0.03	0.00	0.03	0.04	0.00	0.05	0.03	0.00	0.01	0.03	0.05	0.08	0.00	0.00
Na_2O	0.00	0.01	0.00	0.00	0.01	0.00	0.00	0.01	0.01	0.01	0.00	0.00	0.00	0.00	0.01	0.01	0.01	0.00
F	3.42	3.37	3.51	3.63	3.54	2.17	3.49	3.12	3.37	3.59	3.55	3.33	3.26	3.77	3.40	3.39	2.07	3.42
Cl	0.23	0.07	0.06	0.06	0.06	0.05	0.05	0.05	0.05	0.05	0.05	0.05	0.05	0.05	0.05	0.05	0.05	0.04
H_2O	0.10	0.10	0.10	0.10	0.10	0.10	0.10	0.30	0.20	0.10	0.10	0.20	0.20	0.10	0.10	0.10	0.10	0.10
(F+Cl+OH)	0.00	0.00	0.00	0.00	0.00	0.00	0.00	0.00	0.00	0.00	0.00	0.00	0.00	0.00	0.00	0.00	0.00	0.00
sum	100.57	95.37	97.42	97.80	95.17	93.14	96.07	98.08	98.04	97.17	94.72	97.21	97.54	99.06	97.69	97.69	93.25	94.78
O=(F+Cl)	1.49	1.43	1.49	1.54	1.50	0.92	1.48	1.33	1.43	1.52	1.50	1.41	1.38	1.60	1.44	1.44	0.88	1.45
total	99.08	93.93	95.93	96.25	93.66	92.22	94.59	96.75	96.61	95.65	93.21	95.80	96.15	97.46	96.25	96.25	92.37	93.33
(Y+REE)	0.32	0.33	0.33	0.28	0.65	0.52	0.18	0.69	0.28	0.43	0.60	0.27	0.23	0.27	0.49	0.77	0.54	0.30
P	2.95	2.99	2.97	2.97	2.98	3.02	2.96	2.98	2.97	2.96	2.99	2.97	3.01	2.96	2.99	2.98	3.03	2.97
Si	0.01	0.01	0.01	0.01	0.01	0.00	0.01	0.01	0.02	0.01	0.01	0.02	0.01	0.01	0.01	0.01	0.00	0.01
Al	0.00	0.00	0.00	0.00	0.00	0.00	0.00	0.00	0.00	0.00	0.00	0.00	0.00	0.00	0.00	0.00	0.00	0.00
S	0.00	0.00	0.01	0.00	0.00	0.00	0.00	0.00	0.00	0.00	0.00	0.00	0.00	0.00	0.00	0.00	0.00	0.00
Y	0.00	0.00	0.00	0.00	0.00	0.00	0.00	0.00	0.00	0.00	0.00	0.00	0.00	0.00	0.00	0.00	0.00	0.00
La	0.00	0.00	0.00	0.00	0.00	0.00	0.00	0.00	0.00	0.00	0.00	0.00	0.00	0.00	0.00	0.00	0.00	0.00
Ce	0.00	0.00	0.00	0.00	0.01	0.01	0.00	0.01	0.00	0.00	0.01	0.00	0.00	0.00	0.01	0.01	0.01	0.00
Pr	0.00	0.00	0.00	0.00	0.00	0.00	0.00	0.00	0.00	0.00	0.00	0.00	0.00	0.00	0.00	0.00	0.00	0.00
Nd	0.00	0.00	0.00	0.00	0.01	0.01	0.00	0.01	0.00	0.00	0.01	0.00	0.00	0.00	0.01	0.01	0.01	0.00
Sm	0.00	0.00	0.00	0.00	0.00	0.00	0.00	0.00	0.00	0.00	0.00	0.00	0.00	0.00	0.00	0.00	0.00	0.00
Gd	0.00	0.00	0.00	0.00	0.00	0.00	0.00	0.00	0.00	0.00	0.00	0.00	0.00	0.00	0.00	0.00	0.00	0.00
Dy	0.00	0.00	0.00	0.00	0.00	0.00	0.00	0.00	0.00	0.00	0.00	0.00	0.00	0.00	0.00	0.00	0.00	0.00
Ca	5.03	4.95	4.98	4.96	4.88	5.04	4.93	4.90	4.97	4.98	4.89	4.96	4.89	4.98	4.91	4.94	5.04	4.96
Fe	0.08	0.01	0.04	0.07	0.12	0.08	0.21	0.12	0.06	0.07	0.09	0.06	0.08	0.04	0.10	0.07	0.05	0.10
Mn	0.01	0.00	0.00	0.00	0.01	0.00	0.00	0.00	0.00	0.00	0.00	0.00	0.00	0.00	0.00	0.00	0.00	0.00
Sr	0.00	0.00	0.00	0.00	0.00	0.00	0.00	0.00	0.00	0.00	0.00	0.00	0.00	0.00	0.00	0.00	0.00	0.00
Na	0.00	0.00	0.00	0.00	0.00	0.00	0.00	0.00	0.00	0.00	0.00	0.00	0.00	0.00	0.00	0.00	0.00	0.00
F	0.92	0.95	0.97	1.00	1.01	0.63	0.98	0.86	0.93	1.00	1.01	0.93	0.90	1.03	0.94	0.94	0.60	0.98
Cl	0.03	0.01	0.01	0.01	0.01	0.01	0.01	0.01	0.01	0.01	0.01	0.01	0.01	0.01	0.01	0.01	0.01	0.01
OH	0.06	0.06	0.06	0.06	0.06	0.06	0.06	0.17	0.12	0.06	0.06	0.12	0.12	0.06	0.06	0.06	0.06	0.06
Total	1.01	1.02	1.04	1.07	1.08	0.70	1.05	1.04	1.05	1.07	1.08	1.05	1.02	1.09	1.01	1.01	0.67	1.04
XF/XOH	16.21	15.97	16.64	17.23	16.79	10.26	16.53	4.94	7.99	17.02	16.82	7.90	7.74	17.88	16.11	16.09	9.79	16.21
XCl/XOH	0.58	0.18	0.15	0.15	0.14	0.14	0.13	0.04	0.06	0.12	0.12	0.06	0.06	0.12	0.11	0.11	0.11	0.11

Page 24																		
Charnockite sample	20 cont'd																	20
No. on map	41																	41
Sample No.	NIL2-12																	NIL2-12
No of grains analysed	19	20	21	22	23	24	25	26	27	28	29	30	31	32	33	34	35	35
																		Mean
P_2O_5	39.53	40.06	39.75	41.20	40.74	40.76	39.77	40.93	41.21	41.77	41.11	41.73	41.82	39.59	40.26	40.28	40.98	40.23
SiO_2	0.14	0.13	0.14	0.14	0.03	0.14	0.14	0.14	0.24	0.05	0.06	0.15	0.10	0.00	0.00	0.13	0.16	0.12
Al_2O_3	0.00	0.00	0.00	0.00	0.00	0.00	0.00	0.00	0.00	0.00	0.00	0.00	0.00	0.00	0.00	0.00	0.00	0.00
SO_2	0.02	0.00	0.00	0.00	0.01	0.01	0.02	0.01	0.02	0.00	0.01	0.00	0.00	0.02	0.01	0.00	0.00	0.01
Y_2O_3	0.00	0.00	0.01	0.00	0.00	0.01	0.01	0.02	0.00	0.00	0.00	0.00	0.00	0.00	0.02	0.00	0.00	0.01
La_2O_3	0.01	0.03	0.07	0.02	0.01	0.00	0.00	0.00	0.07	0.07	0.00	0.02	0.02	0.01	0.00	0.04	0.06	0.02
Ce_2O_3	0.18	0.10	0.12	0.19	0.02	0.02	0.01	0.07	0.08	0.05	0.08	0.04	0.15	0.07	0.07	0.04	0.13	0.11
Pr_2O_3	0.04	0.03	0.00	0.00	0.00	0.03	0.00	0.00	0.05	0.06	0.01	0.00	0.01	0.07	0.13	0.03	0.00	0.03
Nd_2O_3	0.19	0.10	0.12	0.23	0.07	0.04	0.09	0.10	0.08	0.01	0.06	0.13	0.23	0.16	0.14	0.06	0.06	0.14
Sm_2O_3	0.03	0.12	0.00	0.00	0.00	0.00	0.12	0.04	0.00	0.00	0.00	0.00	0.00	0.00	0.04	0.00	0.00	0.02
Gd_2O_3	0.00	0.07	0.02	0.00	0.00	0.09	0.05	0.00	0.00	0.00	0.00	0.00	0.00	0.00	0.00	0.00	0.00	0.02
Dy_2O_3	0.00	0.03	0.00	0.00	0.00	0.00	0.03	0.00	0.00	0.00	0.00	0.00	0.00	0.00	0.00	0.00	0.00	0.01
CaO	52.10	52.47	52.48	54.43	55.06	53.76	53.37	52.85	54.21	55.23	55.15	54.89	55.16	52.47	52.41	54.57	54.89	53.05
FeO	0.53	0.29	0.23	0.21	0.13	0.17	0.04	0.04	0.37	0.15	0.44	0.40	0.31	0.17	0.17	0.27	0.22	0.39
MnO	0.02	0.01	0.02	0.09	0.00	0.01	0.00	0.01	0.00	0.04	0.04	0.00	0.00	0.01	0.00	0.00	0.00	0.01
SrO	0.04	0.01	0.03	0.00	0.00	0.01	0.00	0.03	0.00	0.00	0.00	0.00	0.00	0.00	0.00	0.00	0.00	0.01
Na_2O	0.01	0.00	0.00	0.00	0.00	0.00	0.00	0.00	0.01	0.00	0.00	0.00	0.00	0.00	0.00	0.00	0.00	0.00
F	3.49	3.53	3.64	3.53	3.31	3.63	3.61	3.73	3.52	3.47	3.45	3.36	3.11	2.09	2.03	3.26	3.75	3.31
Cl	0.04	0.04	0.04	0.04	0.04	0.04	0.03	0.03	0.03	0.03	0.03	0.03	0.03	0.03	0.02	0.02	0.02	0.05
H_2O	0.10	0.10	0.10	0.10	0.30	0.10	0.10	0.10	0.10	0.30	0.30	0.30	0.30	0.10	0.10	0.30	0.10	0.13
(F+Cl+OH)	0.00	0.00	0.00	0.00	0.00	0.00	0.00	0.00	0.00	0.00	0.00	0.00	0.00	0.00	0.00	0.00	0.00	0.00
sum	96.46	97.12	96.77	100.18	99.72	98.81	97.40	98.09	99.99	101.23	100.74	101.05	101.24	94.77	95.40	99.00	100.37	97.69
O=(F+Cl)	1.48	1.50	1.54	1.50	1.40	1.54	1.53	1.58	1.49	1.47	1.46	1.42	1.32	0.88	0.86	1.38	1.58	1.41
total	94.97	95.63	95.22	98.68	98.32	97.27	95.87	96.51	98.50	99.76	99.28	99.63	99.92	93.89	94.54	97.62	98.79	96.28
(Y+REE)	0.45	0.47	0.34	0.44	0.10	0.18	0.30	0.23	0.28	0.19	0.15	0.19	0.41	0.30	0.40	0.17	0.25	0.35
P	2.97	2.98	2.97	2.98	2.96	2.98	2.96	3.00	2.98	2.98	2.95	2.98	2.98	3.03	3.05	2.95	2.96	2.98
Si	0.01	0.01	0.01	0.01	0.00	0.01	0.01	0.01	0.02	0.00	0.01	0.01	0.01	0.00	0.00	0.01	0.01	0.01
Al	0.00	0.00	0.00	0.00	0.00	0.00	0.00	0.00	0.00	0.00	0.00	0.00	0.00	0.00	0.00	0.00	0.00	0.00
S	0.00	0.00	0.00	0.00	0.00	0.00	0.00	0.00	0.00	0.00	0.00	0.00	0.00	0.00	0.00	0.00	0.00	0.00
Y	0.00	0.00	0.00	0.00	0.00	0.00	0.00	0.00	0.00	0.00	0.00	0.00	0.00	0.00	0.00	0.00	0.00	0.00
La	0.00	0.00	0.00	0.00	0.00	0.00	0.00	0.00	0.00	0.00	0.00	0.00	0.00	0.00	0.00	0.00	0.00	0.00
Ce	0.01	0.00	0.00	0.01	0.00	0.00	0.00	0.00	0.00	0.00	0.00	0.00	0.00	0.00	0.00	0.00	0.00	0.00
Pr	0.00	0.00	0.00	0.00	0.00	0.00	0.00	0.00	0.00	0.00	0.00	0.00	0.00	0.00	0.00	0.00	0.00	0.00
Nd	0.01	0.00	0.00	0.01	0.00	0.00	0.00	0.00	0.00	0.00	0.00	0.00	0.01	0.01	0.00	0.00	0.00	0.00
Sm	0.00	0.00	0.00	0.00	0.00	0.00	0.00	0.00	0.00	0.00	0.00	0.00	0.00	0.00	0.00	0.00	0.00	0.00
Gd	0.00	0.00	0.00	0.00	0.00	0.00	0.00	0.00	0.00	0.00	0.00	0.00	0.00	0.00	0.00	0.00	0.00	0.00
Dy	0.00	0.00	0.00	0.00	0.00	0.00	0.00	0.00	0.00	0.00	0.00	0.00	0.00	0.00	0.00	0.00	0.00	0.00
Ca	4.96	4.95	4.97	4.98	5.05	4.97	5.02	4.91	4.96	4.98	5.01	4.96	4.98	5.07	5.02	5.05	5.01	4.98
Fe	0.08	0.04	0.03	0.03	0.02	0.02	0.01	0.01	0.05	0.02	0.06	0.06	0.04	0.03	0.02	0.04	0.03	0.06
Mn	0.00	0.00	0.00	0.01	0.00	0.00	0.00	0.00	0.00	0.01	0.01	0.00	0.00	0.00	0.00	0.00	0.00	0.00
Sr	0.00	0.00	0.00	0.00	0.00	0.00	0.00	0.00	0.00	0.00	0.00	0.00	0.00	0.00	0.00	0.00	0.00	0.00
Na	0.00	0.00	0.00	0.00	0.00	0.00	0.00	0.00	0.00	0.00	0.00	0.00	0.00	0.00	0.00	0.00	0.00	0.00
F	0.98	0.98	1.02	0.95	0.90	0.99	1.00	1.02	0.95	0.92	0.93	0.90	0.83	0.60	0.57	0.89	1.01	0.92
Cl	0.01	0.01	0.01	0.01	0.01	0.01	0.01	0.00	0.00	0.00	0.00	0.00	0.00	0.00	0.00	0.00	0.00	0.01
OH	0.06	0.06	0.06	0.06	0.17	0.06	0.06	0.06	0.06	0.17	0.17	0.17	0.17	0.06	0.06	0.17	0.06	0.08
Total	1.05	1.05	1.08	1.02	1.07	1.06	1.07	1.08	1.01	1.10	1.10	1.07	1.00	0.66	0.64	1.07	1.07	1.00
XF/XOH	16.56	16.75	17.27	16.73	5.23	17.22	17.11	17.66	16.69					9.89	9.62		17.78	14.88
XCl/XOH	0.10	0.10	0.10	0.10	0.03	0.10	0.09	0.09	0.08	0.03	0.03	0.03	0.03	0.07	0.06	0.02	0.05	0.09

Extended Data Table 6: Apatite compositions (wt%) in hornblende-biotite samples.

Page 1				
Hb-Bt Gneiss sample	1			
Sample No.	Nil 27-1			*NIL27-1*
Grain		1	2	*2*
				Mean
P_2O_5		40.37	40.13	*40.25*
SiO_2		0.00	0.00	*0.00*
Al_2O_3		0.00	0.00	*0.00*
SO_2		0.11	0.20	*0.15*
Y_2O_3		0.00	0.06	*0.03*
La_2O_3		0.00	0.00	*0.00*
Ce_2O_3		0.00	0.05	*0.02*
Pr_2O_3		0.00	0.00	*0.00*
Nd_2O_3		0.00	0.04	*0.02*
Sm_2O_3		0.01	0.04	*0.02*
Gd_2O_3		0.04	0.00	*0.02*
Dy_2O_3		0.00	0.00	*0.00*
CaO		55.42	54.86	*55.14*
FeO		0.03	0.05	*0.04*
MnO		0.01	0.05	*0.03*
SrO		0.00	0.00	*0.00*
Na_2O		0.00	0.01	*0.00*
F		2.90	2.49	*2.69*
Cl		0.01	0.01	*0.01*
H_2O		0.40	0.60	*0.50*
(F+Cl+OH)		0.00	0.00	*0.00*
sum		99.29	98.57	*98.93*
O=(F+Cl)		1.22	1.05	*1.14*
total		98.07	97.52	*97.79*
(Y+REE)		0.05	0.19	*0.12*
P		2.94	2.94	*2.94*
Si		0.00	0.00	*0.00*
Al		0.00	0.00	*0.00*
S		0.01	0.03	*0.02*
Y		0.00	0.00	*0.00*
La		0.00	0.00	*0.00*
Ce		0.00	0.00	*0.00*
Pr		0.00	0.00	*0.00*
Nd		0.00	0.00	*0.00*
Sm		0.00	0.00	*0.00*
Gd		0.00	0.00	*0.00*
Dy		0.00	0.00	*0.00*
Ca		5.11	5.08	*5.10*
Fe		0.00	0.01	*0.01*
Mn		0.00	0.01	*0.00*
Sr		0.00	0.00	*0.00*
Na		0.00	0.00	*0.00*
F		0.79	0.68	*0.73*
Cl		0.00	0.00	*0.00*
OH		0.23	0.35	*0.29*
Total		1.02	1.03	*1.02*
XF/XOH		3.43	1.96	*2.70*
XCl/XOH		0.01	0.01	*0.01*

Page 2						
Hb-Bt Gneiss sample	2					
Sample No.	NIL 274					*NIL27-4*
Grain	1	2	3	4	5	*5*
						Mean
P_2O_5	40.25	40.62	40.53	40.93	40.77	*40.62*
SiO_2	0.00	0.00	0.00	0.00	0.00	*0.00*
Al_2O_3	0.00	0.00	0.00	0.00	0.00	*0.00*
SO_2	0.14	0.09	0.14	0.15	0.12	*0.13*
Y_2O_3	0.03	0.02	0.00	0.00	0.00	*0.01*
La_2O_3	0.04	0.03	0.00	0.00	0.00	*0.01*
Ce_2O_3	0.04	0.03	0.03	0.02	0.04	*0.03*
Pr_2O_3	0.01	0.00	0.00	0.19	0.00	*0.04*
Nd_2O_3	0.00	0.04	0.07	0.00	0.00	*0.02*
Sm_2O_3	0.00	0.00	0.00	0.06	0.00	*0.01*
Gd_2O_3	0.05	0.00	0.00	0.03	0.00	*0.02*
Dy_2O_3	0.06	0.00	0.07	0.00	0.00	*0.02*
CaO	54.60	54.81	54.68	55.04	55.12	*54.85*
FeO	0.04	0.04	0.07	0.00	0.07	*0.04*
MnO	0.02	0.05	0.00	0.00	0.00	*0.02*
SrO	0.00	0.00	0.00	0.00	0.00	*0.00*
Na_2O	0.02	0.00	0.00	0.01	0.02	*0.01*
F	3.35	3.70	3.23	3.82	3.82	*3.58*
Cl	0.00	0.00	0.00	0.01	0.00	*0.00*
H_2O	0.30	0.10	0.30	0.10	0.10	*0.18*
(F+Cl+OH)	0.00	0.00	0.00	0.00	0.00	*0.00*
sum	98.94	99.54	99.11	100.35	100.06	*99.60*
O=(F+Cl)	1.41	1.56	1.36	1.61	1.61	*1.51*
total	97.52	97.98	97.75	98.74	98.45	*98.09*
(Y+REE)	0.21	0.13	0.17	0.31	0.05	*0.17*
P	2.94	2.96	2.96	2.95	2.95	*2.95*
Si	0.00	0.00	0.00	0.00	0.00	*0.00*
Al	0.00	0.00	0.00	0.00	0.00	*0.00*
S	0.02	0.01	0.02	0.02	0.02	*0.02*
Y	0.00	0.00	0.00	0.00	0.00	*0.00*
La	0.00	0.00	0.00	0.00	0.00	*0.00*
Ce	0.00	0.00	0.00	0.00	0.00	*0.00*
Pr	0.00	0.00	0.00	0.00	0.00	*0.00*
Nd	0.00	0.00	0.00	0.00	0.00	*0.00*
Sm	0.00	0.00	0.00	0.00	0.00	*0.00*
Gd	0.00	0.00	0.00	0.00	0.00	*0.00*
Dy	0.00	0.00	0.00	0.00	0.00	*0.00*
Ca	5.05	5.05	5.05	5.03	5.05	*5.04*
Fe	0.01	0.01	0.01	0.00	0.01	*0.01*
Mn	0.00	0.01	0.00	0.00	0.00	*0.00*
Sr	0.00	0.00	0.00	0.00	0.00	*0.00*
Na	0.00	0.00	0.00	0.00	0.00	*0.00*
F	0.92	1.01	0.88	1.03	1.03	*0.97*
Cl	0.00	0.00	0.00	0.00	0.00	*0.00*
OH	0.17	0.06	0.17	0.06	0.06	*0.10*
Total	1.09	1.06	1.05	1.09	1.09	*1.08*
XF/XOH	5.29	17.54	5.10	18.11	18.12	*12.83*
XCl/XOH	0.00	0.01	0.00	0.01	0.00	*0.00*

Page 3					
Hb-Bt Gneiss sample	3				
Sample No.	2714	2714	2714	2714	*NIL27-14*
Grain	1	2	3	4	*4*
					Mean
P_2O_5	41.17	34.43	41.01	41.56	*39.54*
SiO_2	0.00	3.58	0.00	0.00	*0.90*
Al_2O_3	0.00	0.00	0.00	0.00	*0.00*
SO_2	0.05	0.00	0.02	0.15	*0.05*
Y_2O_3	0.10	0.09	0.06	0.07	*0.08*
La_2O_3	0.00	0.15	0.00	0.00	*0.04*
Ce_2O_3	0.03	0.49	0.05	0.00	*0.14*
Pr_2O_3	0.01	0.00	0.00	0.04	*0.01*
Nd_2O_3	0.00	0.40	0.00	0.01	*0.10*
Sm_2O_3	0.00	0.00	0.00	0.00	*0.00*
Gd_2O_3	0.02	0.00	0.03	0.00	*0.01*
Dy_2O_3	0.01	0.01	0.02	0.03	*0.02*
CaO	56.29	50.37	55.54	54.46	*54.16*
FeO	0.10	2.68	0.10	0.31	*0.80*
MnO	0.06	0.05	0.01	0.03	*0.04*
SrO	0.00	0.00	0.00	0.00	*0.00*
Na_2O	0.00	0.00	0.00	0.00	*0.00*
F	1.92	1.81	2.07	1.32	*1.78*
Cl	0.11	0.06	0.08	0.06	*0.08*
H_2O	1.00	0.90	0.90	1.20	*1.00*
(F+Cl+OH)	0.00	0.00	0.00	0.00	*0.00*
sum	100.88	95.03	99.89	99.23	*98.76*
O=(F+Cl)	0.83	0.77	0.89	0.57	*0.77*
total	100.05	94.25	99.00	98.66	*97.99*
(Y+REE)	0.17	1.15	0.16	0.15	*0.41*
P	2.94	2.65	2.95	2.99	*2.88*
Si	0.00	0.33	0.00	0.00	*0.08*
Al	0.00	0.00	0.00	0.00	*0.00*
S	0.01	0.00	0.00	0.02	*0.01*
Y	0.00	0.00	0.00	0.00	*0.00*
La	0.00	0.01	0.00	0.00	*0.00*
Ce	0.00	0.02	0.00	0.00	*0.00*
Pr	0.00	0.00	0.00	0.00	*0.00*
Nd	0.00	0.01	0.00	0.00	*0.00*
Sm	0.00	0.00	0.00	0.00	*0.00*
Gd	0.00	0.00	0.00	0.00	*0.00*
Dy	0.00	0.00	0.00	0.00	*0.00*
Ca	5.08	4.91	5.06	4.95	*5.00*
Fe	0.01	0.41	0.01	0.04	*0.12*
Mn	0.01	0.01	0.00	0.00	*0.01*
Sr	0.00	0.00	0.00	0.00	*0.00*
Na	0.00	0.00	0.00	0.00	*0.00*
F	0.51	0.52	0.56	0.35	*0.49*
Cl	0.02	0.01	0.01	0.01	*0.01*
OH	0.56	0.55	0.51	0.68	*0.57*
Total	1.09	1.08	1.08	1.04	*1.07*
XF/XOH	0.91	0.95	1.09	0.52	*0.87*
XCl/XOH	0.03	0.02	0.02	0.01	*0.02*
